# Measurement of three-jet production cross-sections in $pp$ collisions at 7 $\mathrm {\ Te\,V}$ centre-of-mass energy using the ATLAS detector

**DOI:** 10.1140/epjc/s10052-015-3363-3

**Published:** 2015-05-27

**Authors:** ATLAS Collaboration, G. Aad, B. Abbott, J. Abdallah, S. Abdel Khalek, O. Abdinov, R. Aben, B. Abi, M. Abolins, O. S. AbouZeid, H. Abramowicz, H. Abreu, R. Abreu, Y. Abulaiti, B. S. Acharya, L. Adamczyk, D. L. Adams, J. Adelman, S. Adomeit, T. Adye, T. Agatonovic-Jovin, J. A. Aguilar-Saavedra, M. Agustoni, S. P. Ahlen, F. Ahmadov, G. Aielli, H. Akerstedt, T. P. A. Åkesson, G. Akimoto, A. V. Akimov, G. L. Alberghi, J. Albert, S. Albrand, M. J. Alconada Verzini, M. Aleksa, I. N. Aleksandrov, C. Alexa, G. Alexander, G. Alexandre, T. Alexopoulos, M. Alhroob, G. Alimonti, L. Alio, J. Alison, B. M. M. Allbrooke, L. J. Allison, P. P. Allport, J. Almond, A. Aloisio, A. Alonso, F. Alonso, C. Alpigiani, A. Altheimer, B. Alvarez Gonzalez, M. G. Alviggi, K. Amako, Y. Amaral Coutinho, C. Amelung, D. Amidei, S. P. Amor Dos Santos, A. Amorim, S. Amoroso, N. Amram, G. Amundsen, C. Anastopoulos, L. S. Ancu, N. Andari, T. Andeen, C. F. Anders, G. Anders, K. J. Anderson, A. Andreazza, V. Andrei, X. S. Anduaga, S. Angelidakis, I. Angelozzi, P. Anger, A. Angerami, F. Anghinolfi, A. V. Anisenkov, N. Anjos, A. Annovi, A. Antonaki, M. Antonelli, A. Antonov, J. Antos, F. Anulli, M. Aoki, L. Aperio Bella, R. Apolle, G. Arabidze, I. Aracena, Y. Arai, J. P. Araque, A. T. H. Arce, J-F. Arguin, S. Argyropoulos, M. Arik, A. J. Armbruster, O. Arnaez, V. Arnal, H. Arnold, M. Arratia, O. Arslan, A. Artamonov, G. Artoni, S. Asai, N. Asbah, A. Ashkenazi, B. Åsman, L. Asquith, K. Assamagan, R. Astalos, M. Atkinson, N. B. Atlay, B. Auerbach, K. Augsten, M. Aurousseau, G. Avolio, G. Azuelos, Y. Azuma, M. A. Baak, A. Baas, C. Bacci, H. Bachacou, K. Bachas, M. Backes, M. Backhaus, J. Backus Mayes, E. Badescu, P. Bagiacchi, P. Bagnaia, Y. Bai, T. Bain, J. T. Baines, O. K. Baker, P. Balek, F. Balli, E. Banas, Sw. Banerjee, A. A. E. Bannoura, V. Bansal, H. S. Bansil, L. Barak, S. P. Baranov, E. L. Barberio, D. Barberis, M. Barbero, T. Barillari, M. Barisonzi, T. Barklow, N. Barlow, B. M. Barnett, R. M. Barnett, Z. Barnovska, A. Baroncelli, G. Barone, A. J. Barr, F. Barreiro, J. Barreiro Guimarães da Costa, R. Bartoldus, A. E. Barton, P. Bartos, V. Bartsch, A. Bassalat, A. Basye, R. L. Bates, J. R. Batley, M. Battaglia, M. Battistin, F. Bauer, H. S. Bawa, M. D. Beattie, T. Beau, P. H. Beauchemin, R. Beccherle, P. Bechtle, H. P. Beck, K. Becker, S. Becker, M. Beckingham, C. Becot, A. J. Beddall, A. Beddall, S. Bedikian, V. A. Bednyakov, C. P. Bee, L. J. Beemster, T. A. Beermann, M. Begel, K. Behr, C. Belanger-Champagne, P. J. Bell, W. H. Bell, G. Bella, L. Bellagamba, A. Bellerive, M. Bellomo, K. Belotskiy, O. Beltramello, O. Benary, D. Benchekroun, K. Bendtz, N. Benekos, Y. Benhammou, E. Benhar Noccioli, J. A. Benitez Garcia, D. P. Benjamin, J. R. Bensinger, K. Benslama, S. Bentvelsen, D. Berge, E. Bergeaas Kuutmann, N. Berger, F. Berghaus, J. Beringer, C. Bernard, P. Bernat, C. Bernius, F. U. Bernlochner, T. Berry, P. Berta, C. Bertella, G. Bertoli, F. Bertolucci, C. Bertsche, D. Bertsche, M. I. Besana, G. J. Besjes, O. Bessidskaia Bylund, M. Bessner, N. Besson, C. Betancourt, S. Bethke, W. Bhimji, R. M. Bianchi, L. Bianchini, M. Bianco, O. Biebel, S. P. Bieniek, K. Bierwagen, J. Biesiada, M. Biglietti, J. Bilbao De Mendizabal, H. Bilokon, M. Bindi, S. Binet, A. Bingul, C. Bini, C. W. Black, J. E. Black, K. M. Black, D. Blackburn, R. E. Blair, J.-B. Blanchard, T. Blazek, I. Bloch, C. Blocker, W. Blum, U. Blumenschein, G. J. Bobbink, V. S. Bobrovnikov, S. S. Bocchetta, A. Bocci, C. Bock, C. R. Boddy, M. Boehler, T. T. Boek, J. A. Bogaerts, A. G. Bogdanchikov, A. Bogouch, C. Bohm, J. Bohm, V. Boisvert, T. Bold, V. Boldea, A. S. Boldyrev, M. Bomben, M. Bona, M. Boonekamp, A. Borisov, G. Borissov, M. Borri, S. Borroni, J. Bortfeldt, V. Bortolotto, K. Bos, D. Boscherini, M. Bosman, H. Boterenbrood, J. Boudreau, J. Bouffard, E. V. Bouhova-Thacker, D. Boumediene, C. Bourdarios, N. Bousson, S. Boutouil, A. Boveia, J. Boyd, I. R. Boyko, J. Bracinik, A. Brandt, G. Brandt, O. Brandt, U. Bratzler, B. Brau, J. E. Brau, H. M. Braun, S. F. Brazzale, B. Brelier, K. Brendlinger, A. J. Brennan, R. Brenner, S. Bressler, K. Bristow, T. M. Bristow, D. Britton, F. M. Brochu, I. Brock, R. Brock, C. Bromberg, J. Bronner, G. Brooijmans, T. Brooks, W. K. Brooks, J. Brosamer, E. Brost, J. Brown, P. A. Bruckman de Renstrom, D. Bruncko, R. Bruneliere, S. Brunet, A. Bruni, G. Bruni, M. Bruschi, L. Bryngemark, T. Buanes, Q. Buat, F. Bucci, P. Buchholz, R. M. Buckingham, A. G. Buckley, S. I. Buda, I. A. Budagov, F. Buehrer, L. Bugge, M. K. Bugge, O. Bulekov, A. C. Bundock, H. Burckhart, S. Burdin, B. Burghgrave, S. Burke, I. Burmeister, E. Busato, D. Büscher, V. Büscher, P. Bussey, C. P. Buszello, B. Butler, J. M. Butler, A. I. Butt, C. M. Buttar, J. M. Butterworth, P. Butti, W. Buttinger, A. Buzatu, M. Byszewski, S. Cabrera Urbán, D. Caforio, O. Cakir, P. Calafiura, A. Calandri, G. Calderini, P. Calfayan, R. Calkins, L. P. Caloba, D. Calvet, S. Calvet, R. Camacho Toro, S. Camarda, D. Cameron, L. M. Caminada, R. Caminal Armadans, S. Campana, M. Campanelli, A. Campoverde, V. Canale, A. Canepa, M. Cano Bret, J. Cantero, R. Cantrill, T. Cao, M. D. M. Capeans Garrido, I. Caprini, M. Caprini, M. Capua, R. Caputo, R. Cardarelli, T. Carli, G. Carlino, L. Carminati, S. Caron, E. Carquin, G. D. Carrillo-Montoya, J. R. Carter, J. Carvalho, D. Casadei, M. P. Casado, M. Casolino, E. Castaneda-Miranda, A. Castelli, V. Castillo Gimenez, N. F. Castro, P. Catastini, A. Catinaccio, J. R. Catmore, A. Cattai, G. Cattani, V. Cavaliere, D. Cavalli, M. Cavalli-Sforza, V. Cavasinni, F. Ceradini, B. Cerio, K. Cerny, A. S. Cerqueira, A. Cerri, L. Cerrito, F. Cerutti, M. Cerv, A. Cervelli, S. A. Cetin, A. Chafaq, D. Chakraborty, I. Chalupkova, P. Chang, B. Chapleau, J. D. Chapman, D. Charfeddine, D. G. Charlton, C. C. Chau, C. A. Chavez Barajas, S. Cheatham, A. Chegwidden, S. Chekanov, S. V. Chekulaev, G. A. Chelkov, M. A. Chelstowska, C. Chen, H. Chen, K. Chen, L. Chen, S. Chen, X. Chen, Y. Chen, Y. Chen, H. C. Cheng, Y. Cheng, A. Cheplakov, R. Cherkaoui El Moursli, V. Chernyatin, E. Cheu, L. Chevalier, V. Chiarella, G. Chiefari, J. T. Childers, A. Chilingarov, G. Chiodini, A. S. Chisholm, R. T. Chislett, A. Chitan, M. V. Chizhov, S. Chouridou, B. K. B. Chow, D. Chromek-Burckhart, M. L. Chu, J. Chudoba, J. J. Chwastowski, L. Chytka, G. Ciapetti, A. K. Ciftci, R. Ciftci, D. Cinca, V. Cindro, A. Ciocio, P. Cirkovic, Z. H. Citron, M. Citterio, M. Ciubancan, A. Clark, P. J. Clark, R. N. Clarke, W. Cleland, J. C. Clemens, C. Clement, Y. Coadou, M. Cobal, A. Coccaro, J. Cochran, L. Coffey, J. G. Cogan, J. Coggeshall, B. Cole, S. Cole, A. P. Colijn, J. Collot, T. Colombo, G. Colon, G. Compostella, P. Conde Muiño, E. Coniavitis, M. C. Conidi, S. H. Connell, I. A. Connelly, S. M. Consonni, V. Consorti, S. Constantinescu, C. Conta, G. Conti, F. Conventi, M. Cooke, B. D. Cooper, A. M. Cooper-Sarkar, N. J. Cooper-Smith, K. Copic, T. Cornelissen, M. Corradi, F. Corriveau, A. Corso-Radu, A. Cortes-Gonzalez, G. Cortiana, G. Costa, M. J. Costa, D. Costanzo, D. Côté, G. Cottin, G. Cowan, B. E. Cox, K. Cranmer, G. Cree, S. Crépé-Renaudin, F. Crescioli, W. A. Cribbs, M. Crispin Ortuzar, M. Cristinziani, V. Croft, G. Crosetti, C.-M. Cuciuc, T. Cuhadar Donszelmann, J. Cummings, M. Curatolo, C. Cuthbert, H. Czirr, P. Czodrowski, Z. Czyczula, S. D’Auria, M. D’Onofrio, M. J. Da Cunha Sargedas De Sousa, C. Da Via, W. Dabrowski, A. Dafinca, T. Dai, O. Dale, F. Dallaire, C. Dallapiccola, M. Dam, A. C. Daniells, M. Dano Hoffmann, V. Dao, G. Darbo, S. Darmora, J. A. Dassoulas, A. Dattagupta, W. Davey, C. David, T. Davidek, E. Davies, M. Davies, O. Davignon, A. R. Davison, P. Davison, Y. Davygora, E. Dawe, I. Dawson, R. K. Daya-Ishmukhametova, K. De, R. de Asmundis, S. De Castro, S. De Cecco, N. De Groot, P. de Jong, H. De la Torre, F. De Lorenzi, L. De Nooij, D. De Pedis, A. De Salvo, U. De Sanctis, A. De Santo, J. B. De Vivie De Regie, W. J. Dearnaley, R. Debbe, C. Debenedetti, B. Dechenaux, D. V. Dedovich, I. Deigaard, J. Del Peso, T. Del Prete, F. Deliot, C. M. Delitzsch, M. Deliyergiyev, A. Dell’Acqua, L. Dell’Asta, M. Dell’Orso, M. Della Pietra, D. della Volpe, M. Delmastro, P. A. Delsart, C. Deluca, S. Demers, M. Demichev, A. Demilly, S. P. Denisov, D. Derendarz, J. E. Derkaoui, F. Derue, P. Dervan, K. Desch, C. Deterre, P. O. Deviveiros, A. Dewhurst, S. Dhaliwal, A. Di Ciaccio, L. Di Ciaccio, A. Di Domenico, C. Di Donato, A. Di Girolamo, B. Di Girolamo, A. Di Mattia, B. Di Micco, R. Di Nardo, A. Di Simone, R. Di Sipio, D. Di Valentino, F. A. Dias, M. A. Diaz, E. B. Diehl, J. Dietrich, T. A. Dietzsch, S. Diglio, A. Dimitrievska, J. Dingfelder, C. Dionisi, P. Dita, S. Dita, F. Dittus, F. Djama, T. Djobava, M. A. B. do Vale, A. Do Valle Wemans, D. Dobos, C. Doglioni, T. Doherty, T. Dohmae, J. Dolejsi, Z. Dolezal, B. A. Dolgoshein, M. Donadelli, S. Donati, P. Dondero, J. Donini, J. Dopke, A. Doria, M. T. Dova, A. T. Doyle, M. Dris, J. Dubbert, S. Dube, E. Dubreuil, E. Duchovni, G. Duckeck, O. A. Ducu, D. Duda, A. Dudarev, F. Dudziak, L. Duflot, L. Duguid, M. Dührssen, M. Dunford, H. Duran Yildiz, M. Düren, A. Durglishvili, M. Dwuznik, M. Dyndal, J. Ebke, W. Edson, N. C. Edwards, W. Ehrenfeld, T. Eifert, G. Eigen, K. Einsweiler, T. Ekelof, M. El Kacimi, M. Ellert, S. Elles, F. Ellinghaus, N. Ellis, J. Elmsheuser, M. Elsing, D. Emeliyanov, Y. Enari, O. C. Endner, M. Endo, R. Engelmann, J. Erdmann, A. Ereditato, D. Eriksson, G. Ernis, J. Ernst, M. Ernst, J. Ernwein, D. Errede, S. Errede, E. Ertel, M. Escalier, H. Esch, C. Escobar, B. Esposito, A. I. Etienvre, E. Etzion, H. Evans, A. Ezhilov, L. Fabbri, G. Facini, R. M. Fakhrutdinov, S. Falciano, R. J. Falla, J. Faltova, Y. Fang, M. Fanti, A. Farbin, A. Farilla, T. Farooque, S. Farrell, S. M. Farrington, P. Farthouat, F. Fassi, P. Fassnacht, D. Fassouliotis, A. Favareto, L. Fayard, P. Federic, O. L. Fedin, W. Fedorko, M. Fehling-Kaschek, S. Feigl, L. Feligioni, C. Feng, E. J. Feng, H. Feng, A. B. Fenyuk, S. Fernandez Perez, S. Ferrag, J. Ferrando, A. Ferrari, P. Ferrari, R. Ferrari, D. E. Ferreira de Lima, A. Ferrer, D. Ferrere, C. Ferretti, A. Ferretto Parodi, M. Fiascaris, F. Fiedler, A. Filipčič, M. Filipuzzi, F. Filthaut, M. Fincke-Keeler, K. D. Finelli, M. C. N. Fiolhais, L. Fiorini, A. Firan, A. Fischer, J. Fischer, W. C. Fisher, E. A. Fitzgerald, M. Flechl, I. Fleck, P. Fleischmann, S. Fleischmann, G. T. Fletcher, G. Fletcher, T. Flick, A. Floderus, L. R. Flores Castillo, A. C. Florez Bustos, M. J. Flowerdew, A. Formica, A. Forti, D. Fortin, D. Fournier, H. Fox, S. Fracchia, P. Francavilla, M. Franchini, S. Franchino, D. Francis, L. Franconi, M. Franklin, S. Franz, M. Fraternali, S. T. French, C. Friedrich, F. Friedrich, D. Froidevaux, J. A. Frost, C. Fukunaga, E. Fullana Torregrosa, B. G. Fulsom, J. Fuster, C. Gabaldon, O. Gabizon, A. Gabrielli, A. Gabrielli, S. Gadatsch, S. Gadomski, G. Gagliardi, P. Gagnon, C. Galea, B. Galhardo, E. J. Gallas, V. Gallo, B. J. Gallop, P. Gallus, G. Galster, K. K. Gan, J. Gao, Y. S. Gao, F. M. Garay Walls, F. Garberson, C. García, J. E. García Navarro, M. Garcia-Sciveres, R. W. Gardner, N. Garelli, V. Garonne, C. Gatti, G. Gaudio, B. Gaur, L. Gauthier, P. Gauzzi, I. L. Gavrilenko, C. Gay, G. Gaycken, E. N. Gazis, P. Ge, Z. Gecse, C. N. P. Gee, D. A. A. Geerts, Ch. Geich-Gimbel, K. Gellerstedt, C. Gemme, A. Gemmell, M. H. Genest, S. Gentile, M. George, S. George, D. Gerbaudo, A. Gershon, H. Ghazlane, N. Ghodbane, B. Giacobbe, S. Giagu, V. Giangiobbe, P. Giannetti, F. Gianotti, B. Gibbard, S. M. Gibson, M. Gilchriese, T. P. S. Gillam, D. Gillberg, G. Gilles, D. M. Gingrich, N. Giokaris, M. P. Giordani, R. Giordano, F. M. Giorgi, F. M. Giorgi, P. F. Giraud, D. Giugni, C. Giuliani, M. Giulini, B. K. Gjelsten, S. Gkaitatzis, I. Gkialas, L. K. Gladilin, C. Glasman, J. Glatzer, P. C. F. Glaysher, A. Glazov, G. L. Glonti, M. Goblirsch-Kolb, J. R. Goddard, J. Godlewski, C. Goeringer, S. Goldfarb, T. Golling, D. Golubkov, A. Gomes, L. S. Gomez Fajardo, R. Gonçalo, J. Goncalves Pinto Firmino Da Costa, L. Gonella, S. González de la Hoz, G. Gonzalez Parra, S. Gonzalez-Sevilla, L. Goossens, P. A. Gorbounov, H. A. Gordon, I. Gorelov, B. Gorini, E. Gorini, A. Gorišek, E. Gornicki, A. T. Goshaw, C. Gössling, M. I. Gostkin, M. Gouighri, D. Goujdami, M. P. Goulette, A. G. Goussiou, C. Goy, S. Gozpinar, H. M. X. Grabas, L. Graber, I. Grabowska-Bold, P. Grafström, K-J. Grahn, J. Gramling, E. Gramstad, S. Grancagnolo, V. Grassi, V. Gratchev, H. M. Gray, E. Graziani, O. G. Grebenyuk, Z. D. Greenwood, K. Gregersen, I. M. Gregor, P. Grenier, J. Griffiths, A. A. Grillo, K. Grimm, S. Grinstein, Ph. Gris, Y. V. Grishkevich, J.-F. Grivaz, J. P. Grohs, A. Grohsjean, E. Gross, J. Grosse-Knetter, G. C. Grossi, J. Groth-Jensen, Z. J. Grout, L. Guan, F. Guescini, D. Guest, O. Gueta, C. Guicheney, E. Guido, T. Guillemin, S. Guindon, U. Gul, C. Gumpert, J. Gunther, J. Guo, S. Gupta, P. Gutierrez, N. G. Gutierrez Ortiz, C. Gutschow, N. Guttman, C. Guyot, C. Gwenlan, C. B. Gwilliam, A. Haas, C. Haber, H. K. Hadavand, N. Haddad, P. Haefner, S. Hageböck, Z. Hajduk, H. Hakobyan, M. Haleem, D. Hall, G. Halladjian, K. Hamacher, P. Hamal, K. Hamano, M. Hamer, A. Hamilton, S. Hamilton, G. N. Hamity, P. G. Hamnett, L. Han, K. Hanagaki, K. Hanawa, M. Hance, P. Hanke, R. Hanna, J. B. Hansen, J. D. Hansen, P. H. Hansen, K. Hara, A. S. Hard, T. Harenberg, F. Hariri, S. Harkusha, D. Harper, R. D. Harrington, O. M. Harris, P. F. Harrison, F. Hartjes, M. Hasegawa, S. Hasegawa, Y. Hasegawa, A. Hasib, S. Hassani, S. Haug, M. Hauschild, R. Hauser, M. Havranek, C. M. Hawkes, R. J. Hawkings, A. D. Hawkins, T. Hayashi, D. Hayden, C. P. Hays, H. S. Hayward, S. J. Haywood, S. J. Head, T. Heck, V. Hedberg, L. Heelan, S. Heim, T. Heim, B. Heinemann, L. Heinrich, J. Hejbal, L. Helary, C. Heller, M. Heller, S. Hellman, D. Hellmich, C. Helsens, J. Henderson, R. C. W. Henderson, Y. Heng, C. Hengler, A. Henrichs, A. M. Henriques Correia, S. Henrot-Versille, C. Hensel, G. H. Herbert, Y. Hernández Jiménez, R. Herrberg-Schubert, G. Herten, R. Hertenberger, L. Hervas, G. G. Hesketh, N. P. Hessey, R. Hickling, E. Higón-Rodriguez, E. Hill, J. C. Hill, K. H. Hiller, S. Hillert, S. J. Hillier, I. Hinchliffe, E. Hines, M. Hirose, D. Hirschbuehl, J. Hobbs, N. Hod, M. C. Hodgkinson, P. Hodgson, A. Hoecker, M. R. Hoeferkamp, F. Hoenig, J. Hoffman, D. Hoffmann, J. I. Hofmann, M. Hohlfeld, T. R. Holmes, T. M. Hong, L. Hooft van Huysduynen, W. H. Hopkins, Y. Horii, J-Y. Hostachy, S. Hou, A. Hoummada, J. Howard, J. Howarth, M. Hrabovsky, I. Hristova, J. Hrivnac, T. Hryn’ova, A. Hrynevich, C. Hsu, P. J. Hsu, S.-C. Hsu, D. Hu, X. Hu, Y. Huang, Z. Hubacek, F. Hubaut, F. Huegging, T. B. Huffman, E. W. Hughes, G. Hughes, M. Huhtinen, T. A. Hülsing, M. Hurwitz, N. Huseynov, J. Huston, J. Huth, G. Iacobucci, G. Iakovidis, I. Ibragimov, L. Iconomidou-Fayard, E. Ideal, P. Iengo, O. Igonkina, T. Iizawa, Y. Ikegami, K. Ikematsu, M. Ikeno, Y. Ilchenko, D. Iliadis, N. Ilic, Y. Inamaru, T. Ince, P. Ioannou, M. Iodice, K. Iordanidou, V. Ippolito, A. Irles Quiles, C. Isaksson, M. Ishino, M. Ishitsuka, R. Ishmukhametov, C. Issever, S. Istin, J. M. Iturbe Ponce, R. Iuppa, J. Ivarsson, W. Iwanski, H. Iwasaki, J. M. Izen, V. Izzo, B. Jackson, M. Jackson, P. Jackson, M. R. Jaekel, V. Jain, K. Jakobs, S. Jakobsen, T. Jakoubek, J. Jakubek, D. O. Jamin, D. K. Jana, E. Jansen, H. Jansen, J. Janssen, M. Janus, G. Jarlskog, N. Javadov, T. Javůrek, L. Jeanty, J. Jejelava, G.-Y. Jeng, D. Jennens, P. Jenni, J. Jentzsch, C. Jeske, S. Jézéquel, H. Ji, J. Jia, Y. Jiang, M. Jimenez Belenguer, S. Jin, A. Jinaru, O. Jinnouchi, M. D. Joergensen, K. E. Johansson, P. Johansson, K. A. Johns, K. Jon-And, G. Jones, R. W. L. Jones, T. J. Jones, J. Jongmanns, P. M. Jorge, K. D. Joshi, J. Jovicevic, X. Ju, C. A. Jung, R. M. Jungst, P. Jussel, A. Juste Rozas, M. Kaci, A. Kaczmarska, M. Kado, H. Kagan, M. Kagan, E. Kajomovitz, C. W. Kalderon, S. Kama, A. Kamenshchikov, N. Kanaya, M. Kaneda, S. Kaneti, V. A. Kantserov, J. Kanzaki, B. Kaplan, A. Kapliy, D. Kar, K. Karakostas, N. Karastathis, M. J. Kareem, M. Karnevskiy, S. N. Karpov, Z. M. Karpova, K. Karthik, V. Kartvelishvili, A. N. Karyukhin, L. Kashif, G. Kasieczka, R. D. Kass, A. Kastanas, Y. Kataoka, A. Katre, J. Katzy, V. Kaushik, K. Kawagoe, T. Kawamoto, G. Kawamura, S. Kazama, V. F. Kazanin, M. Y. Kazarinov, R. Keeler, R. Kehoe, M. Keil, J. S. Keller, J. J. Kempster, H. Keoshkerian, O. Kepka, B. P. Kerševan, S. Kersten, K. Kessoku, J. Keung, F. Khalil-zada, H. Khandanyan, A. Khanov, A. Khodinov, A. Khomich, T. J. Khoo, G. Khoriauli, A. Khoroshilov, V. Khovanskiy, E. Khramov, J. Khubua, H. Y. Kim, H. Kim, S. H. Kim, N. Kimura, O. Kind, B. T. King, M. King, R. S. B. King, S. B. King, J. Kirk, A. E. Kiryunin, T. Kishimoto, D. Kisielewska, F. Kiss, T. Kittelmann, K. Kiuchi, E. Kladiva, M. Klein, U. Klein, K. Kleinknecht, P. Klimek, A. Klimentov, R. Klingenberg, J. A. Klinger, T. Klioutchnikova, P. F. Klok, E.-E. Kluge, P. Kluit, S. Kluth, E. Kneringer, E. B. F. G. Knoops, A. Knue, D. Kobayashi, T. Kobayashi, M. Kobel, M. Kocian, P. Kodys, P. Koevesarki, T. Koffas, E. Koffeman, L. A. Kogan, S. Kohlmann, Z. Kohout, T. Kohriki, T. Koi, H. Kolanoski, I. Koletsou, J. Koll, A. A. Komar, Y. Komori, T. Kondo, N. Kondrashova, K. Köneke, A. C. König, S. König, T. Kono, R. Konoplich, N. Konstantinidis, R. Kopeliansky, S. Koperny, L. Köpke, A. K. Kopp, K. Korcyl, K. Kordas, A. Korn, A. A. Korol, I. Korolkov, E. V. Korolkova, V. A. Korotkov, O. Kortner, S. Kortner, V. V. Kostyukhin, V. M. Kotov, A. Kotwal, C. Kourkoumelis, V. Kouskoura, A. Koutsman, R. Kowalewski, T. Z. Kowalski, W. Kozanecki, A. S. Kozhin, V. Kral, V. A. Kramarenko, G. Kramberger, D. Krasnopevtsev, M. W. Krasny, A. Krasznahorkay, J. K. Kraus, A. Kravchenko, S. Kreiss, M. Kretz, J. Kretzschmar, K. Kreutzfeldt, P. Krieger, K. Kroeninger, H. Kroha, J. Kroll, J. Kroseberg, J. Krstic, U. Kruchonak, H. Krüger, T. Kruker, N. Krumnack, Z. V. Krumshteyn, A. Kruse, M. C. Kruse, M. Kruskal, T. Kubota, S. Kuday, S. Kuehn, A. Kugel, A. Kuhl, T. Kuhl, V. Kukhtin, Y. Kulchitsky, S. Kuleshov, M. Kuna, J. Kunkle, A. Kupco, H. Kurashige, Y. A. Kurochkin, R. Kurumida, V. Kus, E. S. Kuwertz, M. Kuze, J. Kvita, A. La Rosa, L. La Rotonda, C. Lacasta, F. Lacava, J. Lacey, H. Lacker, D. Lacour, V. R. Lacuesta, E. Ladygin, R. Lafaye, B. Laforge, T. Lagouri, S. Lai, H. Laier, L. Lambourne, S. Lammers, C. L. Lampen, W. Lampl, E. Lançon, U. Landgraf, M. P. J. Landon, V. S. Lang, A. J. Lankford, F. Lanni, K. Lantzsch, S. Laplace, C. Lapoire, J. F. Laporte, T. Lari, F. Lasagni Manghi, M. Lassnig, P. Laurelli, W. Lavrijsen, A. T. Law, P. Laycock, O. Le Dortz, E. Le Guirriec, E. Le Menedeu, T. LeCompte, F. Ledroit-Guillon, C. A. Lee, H. Lee, J. S. H. Lee, S. C. Lee, L. Lee, G. Lefebvre, M. Lefebvre, F. Legger, C. Leggett, A. Lehan, M. Lehmacher, G. Lehmann Miotto, X. Lei, W. A. Leight, A. Leisos, A. G. Leister, M. A. L. Leite, R. Leitner, D. Lellouch, B. Lemmer, K. J. C. Leney, T. Lenz, G. Lenzen, B. Lenzi, R. Leone, S. Leone, C. Leonidopoulos, S. Leontsinis, C. Leroy, C. G. Lester, C. M. Lester, M. Levchenko, J. Levêque, D. Levin, L. J. Levinson, M. Levy, A. Lewis, G. H. Lewis, A. M. Leyko, M. Leyton, B. Li, B. Li, H. Li, H. L. Li, L. Li, L. Li, S. Li, Y. Li, Z. Liang, H. Liao, B. Liberti, P. Lichard, K. Lie, J. Liebal, W. Liebig, C. Limbach, A. Limosani, S. C. Lin, T. H. Lin, F. Linde, B. E. Lindquist, J. T. Linnemann, E. Lipeles, A. Lipniacka, M. Lisovyi, T. M. Liss, D. Lissauer, A. Lister, A. M. Litke, B. Liu, D. Liu, J. B. Liu, K. Liu, L. Liu, M. Liu, M. Liu, Y. Liu, M. Livan, S. S. A. Livermore, A. Lleres, J. Llorente Merino, S. L. Lloyd, F. Lo Sterzo, E. Lobodzinska, P. Loch, W. S. Lockman, T. Loddenkoetter, F. K. Loebinger, A. E. Loevschall-Jensen, A. Loginov, T. Lohse, K. Lohwasser, M. Lokajicek, V. P. Lombardo, B. A. Long, J. D. Long, R. E. Long, L. Lopes, D. Lopez Mateos, B. Lopez Paredes, I. Lopez Paz, J. Lorenz, N. Lorenzo Martinez, M. Losada, P. Loscutoff, X. Lou, A. Lounis, J. Love, P. A. Love, A. J. Lowe, F. Lu, N. Lu, H. J. Lubatti, C. Luci, A. Lucotte, F. Luehring, W. Lukas, L. Luminari, O. Lundberg, B. Lund-Jensen, M. Lungwitz, D. Lynn, R. Lysak, E. Lytken, H. Ma, L. L. Ma, G. Maccarrone, A. Macchiolo, J. Machado Miguens, D. Macina, D. Madaffari, R. Madar, H. J. Maddocks, W. F. Mader, A. Madsen, M. Maeno, T. Maeno, A. Maevskiy, E. Magradze, K. Mahboubi, J. Mahlstedt, S. Mahmoud, C. Maiani, C. Maidantchik, A. A. Maier, A. Maio, S. Majewski, Y. Makida, N. Makovec, P. Mal, B. Malaescu, Pa. Malecki, V. P. Maleev, F. Malek, U. Mallik, D. Malon, C. Malone, S. Maltezos, V. M. Malyshev, S. Malyukov, J. Mamuzic, B. Mandelli, L. Mandelli, I. Mandić, R. Mandrysch, J. Maneira, A. Manfredini, L. Manhaes de Andrade Filho, J. A. Manjarres Ramos, A. Mann, P. M. Manning, A. Manousakis-Katsikakis, B. Mansoulie, R. Mantifel, L. Mapelli, L. March, J. F. Marchand, G. Marchiori, M. Marcisovsky, C. P. Marino, M. Marjanovic, C. N. Marques, F. Marroquim, S. P. Marsden, Z. Marshall, L. F. Marti, S. Marti-Garcia, B. Martin, B. Martin, T. A. Martin, V. J. Martin, B. Martin dit Latour, H. Martinez, M. Martinez, S. Martin-Haugh, A. C. Martyniuk, M. Marx, F. Marzano, A. Marzin, L. Masetti, T. Mashimo, R. Mashinistov, J. Masik, A. L. Maslennikov, I. Massa, L. Massa, N. Massol, P. Mastrandrea, A. Mastroberardino, T. Masubuchi, P. Mättig, J. Mattmann, J. Maurer, S. J. Maxfield, D. A. Maximov, R. Mazini, L. Mazzaferro, G. Mc Goldrick, S. P. Mc Kee, A. McCarn, R. L. McCarthy, T. G. McCarthy, N. A. McCubbin, K. W. McFarlane, J. A. Mcfayden, G. Mchedlidze, S. J. McMahon, R. A. McPherson, J. Mechnich, M. Medinnis, S. Meehan, S. Mehlhase, A. Mehta, K. Meier, C. Meineck, B. Meirose, C. Melachrinos, B. R. Mellado Garcia, F. Meloni, A. Mengarelli, S. Menke, E. Meoni, K. M. Mercurio, S. Mergelmeyer, N. Meric, P. Mermod, L. Merola, C. Meroni, F. S. Merritt, H. Merritt, A. Messina, J. Metcalfe, A. S. Mete, C. Meyer, C. Meyer, J-P. Meyer, J. Meyer, R. P. Middleton, S. Migas, L. Mijović, G. Mikenberg, M. Mikestikova, M. Mikuž, A. Milic, D. W. Miller, C. Mills, A. Milov, D. A. Milstead, D. Milstein, A. A. Minaenko, I. A. Minashvili, A. I. Mincer, B. Mindur, M. Mineev, Y. Ming, L. M. Mir, G. Mirabelli, T. Mitani, J. Mitrevski, V. A. Mitsou, S. Mitsui, A. Miucci, P. S. Miyagawa, J. U. Mjörnmark, T. Moa, K. Mochizuki, S. Mohapatra, W. Mohr, S. Molander, R. Moles-Valls, K. Mönig, C. Monini, J. Monk, E. Monnier, J. Montejo Berlingen, F. Monticelli, S. Monzani, R. W. Moore, N. Morange, D. Moreno, M. Moreno Llácer, P. Morettini, M. Morgenstern, M. Morii, S. Moritz, A. K. Morley, G. Mornacchi, J. D. Morris, L. Morvaj, H. G. Moser, M. Mosidze, J. Moss, K. Motohashi, R. Mount, E. Mountricha, S. V. Mouraviev, E. J. W. Moyse, S. Muanza, R. D. Mudd, F. Mueller, J. Mueller, K. Mueller, T. Mueller, T. Mueller, D. Muenstermann, Y. Munwes, J. A. Murillo Quijada, W. J. Murray, H. Musheghyan, E. Musto, A. G. Myagkov, M. Myska, O. Nackenhorst, J. Nadal, K. Nagai, R. Nagai, Y. Nagai, K. Nagano, A. Nagarkar, Y. Nagasaka, M. Nagel, A. M. Nairz, Y. Nakahama, K. Nakamura, T. Nakamura, I. Nakano, H. Namasivayam, G. Nanava, R. Narayan, T. Nattermann, T. Naumann, G. Navarro, R. Nayyar, H. A. Neal, P. Yu. Nechaeva, T. J. Neep, P. D. Nef, A. Negri, G. Negri, M. Negrini, S. Nektarijevic, A. Nelson, T. K. Nelson, S. Nemecek, P. Nemethy, A. A. Nepomuceno, M. Nessi, M. S. Neubauer, M. Neumann, R. M. Neves, P. Nevski, P. R. Newman, D. H. Nguyen, R. B. Nickerson, R. Nicolaidou, B. Nicquevert, J. Nielsen, N. Nikiforou, A. Nikiforov, V. Nikolaenko, I. Nikolic-Audit, K. Nikolics, K. Nikolopoulos, P. Nilsson, Y. Ninomiya, A. Nisati, R. Nisius, T. Nobe, L. Nodulman, M. Nomachi, I. Nomidis, S. Norberg, M. Nordberg, O. Novgorodova, S. Nowak, M. Nozaki, L. Nozka, K. Ntekas, G. Nunes Hanninger, T. Nunnemann, E. Nurse, F. Nuti, B. J. O’Brien, F. O’grady, D. C. O’Neil, V. O’Shea, F. G. Oakham, H. Oberlack, T. Obermann, J. Ocariz, A. Ochi, M. I. Ochoa, S. Oda, S. Odaka, H. Ogren, A. Oh, S. H. Oh, C. C. Ohm, H. Ohman, W. Okamura, H. Okawa, Y. Okumura, T. Okuyama, A. Olariu, A. G. Olchevski, S. A. Olivares Pino, D. Oliveira Damazio, E. Oliver Garcia, A. Olszewski, J. Olszowska, A. Onofre, P. U. E. Onyisi, C. J. Oram, M. J. Oreglia, Y. Oren, D. Orestano, N. Orlando, C. Oropeza Barrera, R. S. Orr, B. Osculati, R. Ospanov, G. Otero y Garzon, H. Otono, M. Ouchrif, E. A. Ouellette, F. Ould-Saada, A. Ouraou, K. P. Oussoren, Q. Ouyang, A. Ovcharova, M. Owen, V. E. Ozcan, N. Ozturk, K. Pachal, A. Pacheco Pages, C. Padilla Aranda, M. Pagáčová, S. Pagan Griso, E. Paganis, C. Pahl, F. Paige, P. Pais, K. Pajchel, G. Palacino, S. Palestini, M. Palka, D. Pallin, A. Palma, J. D. Palmer, Y. B. Pan, E. Panagiotopoulou, J. G. Panduro Vazquez, P. Pani, N. Panikashvili, S. Panitkin, D. Pantea, L. Paolozzi, Th. D. Papadopoulou, K. Papageorgiou, A. Paramonov, D. Paredes Hernandez, M. A. Parker, F. Parodi, J. A. Parsons, U. Parzefall, E. Pasqualucci, S. Passaggio, A. Passeri, F. Pastore, Fr. Pastore, G. Pásztor, S. Pataraia, N. D. Patel, J. R. Pater, S. Patricelli, T. Pauly, J. Pearce, L. E. Pedersen, M. Pedersen, S. Pedraza Lopez, R. Pedro, S. V. Peleganchuk, D. Pelikan, H. Peng, B. Penning, J. Penwell, D. V. Perepelitsa, E. Perez Codina, M. T. Pérez García-Estañ, V. Perez Reale, L. Perini, H. Pernegger, S. Perrella, R. Perrino, R. Peschke, V. D. Peshekhonov, K. Peters, R. F. Y. Peters, B. A. Petersen, T. C. Petersen, E. Petit, A. Petridis, C. Petridou, E. Petrolo, F. Petrucci, N. E. Pettersson, R. Pezoa, P. W. Phillips, G. Piacquadio, E. Pianori, A. Picazio, E. Piccaro, M. Piccinini, R. Piegaia, D. T. Pignotti, J. E. Pilcher, A. D. Pilkington, J. Pina, M. Pinamonti, A. Pinder, J. L. Pinfold, A. Pingel, B. Pinto, S. Pires, M. Pitt, C. Pizio, L. Plazak, M.-A. Pleier, V. Pleskot, E. Plotnikova, P. Plucinski, S. Poddar, F. Podlyski, R. Poettgen, L. Poggioli, D. Pohl, M. Pohl, G. Polesello, A. Policicchio, R. Polifka, A. Polini, C. S. Pollard, V. Polychronakos, K. Pommès, L. Pontecorvo, B. G. Pope, G. A. Popeneciu, D. S. Popovic, A. Poppleton, X. Portell Bueso, S. Pospisil, K. Potamianos, I. N. Potrap, C. J. Potter, C. T. Potter, G. Poulard, J. Poveda, V. Pozdnyakov, P. Pralavorio, A. Pranko, S. Prasad, R. Pravahan, S. Prell, D. Price, J. Price, L. E. Price, D. Prieur, M. Primavera, M. Proissl, K. Prokofiev, F. Prokoshin, E. Protopapadaki, S. Protopopescu, J. Proudfoot, M. Przybycien, H. Przysiezniak, E. Ptacek, D. Puddu, E. Pueschel, D. Puldon, M. Purohit, P. Puzo, J. Qian, G. Qin, Y. Qin, A. Quadt, D. R. Quarrie, W. B. Quayle, M. Queitsch-Maitland, D. Quilty, A. Qureshi, V. Radeka, V. Radescu, S. K. Radhakrishnan, P. Radloff, P. Rados, F. Ragusa, G. Rahal, S. Rajagopalan, M. Rammensee, A. S. Randle-Conde, C. Rangel-Smith, K. Rao, F. Rauscher, T. C. Rave, T. Ravenscroft, M. Raymond, A. L. Read, N. P. Readioff, D. M. Rebuzzi, A. Redelbach, G. Redlinger, R. Reece, K. Reeves, L. Rehnisch, H. Reisin, M. Relich, C. Rembser, H. Ren, Z. L. Ren, A. Renaud, M. Rescigno, S. Resconi, O. L. Rezanova, P. Reznicek, R. Rezvani, R. Richter, M. Ridel, P. Rieck, J. Rieger, M. Rijssenbeek, A. Rimoldi, L. Rinaldi, E. Ritsch, I. Riu, F. Rizatdinova, E. Rizvi, S. H. Robertson, A. Robichaud-Veronneau, D. Robinson, J. E. M. Robinson, A. Robson, C. Roda, L. Rodrigues, S. Roe, O. Røhne, S. Rolli, A. Romaniouk, M. Romano, E. Romero Adam, N. Rompotis, M. Ronzani, L. Roos, E. Ros, S. Rosati, K. Rosbach, M. Rose, P. Rose, P. L. Rosendahl, O. Rosenthal, V. Rossetti, E. Rossi, L. P. Rossi, R. Rosten, M. Rotaru, I. Roth, J. Rothberg, D. Rousseau, C. R. Royon, A. Rozanov, Y. Rozen, X. Ruan, F. Rubbo, I. Rubinskiy, V. I. Rud, C. Rudolph, M. S. Rudolph, F. Rühr, A. Ruiz-Martinez, Z. Rurikova, N. A. Rusakovich, A. Ruschke, J. P. Rutherfoord, N. Ruthmann, Y. F. Ryabov, M. Rybar, G. Rybkin, N. C. Ryder, A. F. Saavedra, S. Sacerdoti, A. Saddique, I. Sadeh, H. F-W. Sadrozinski, R. Sadykov, F. Safai Tehrani, H. Sakamoto, Y. Sakurai, G. Salamanna, A. Salamon, M. Saleem, D. Salek, P. H. Sales De Bruin, D. Salihagic, A. Salnikov, J. Salt, D. Salvatore, F. Salvatore, A. Salvucci, A. Salzburger, D. Sampsonidis, A. Sanchez, J. Sánchez, V. Sanchez Martinez, H. Sandaker, R. L. Sandbach, H. G. Sander, M. P. Sanders, M. Sandhoff, T. Sandoval, C. Sandoval, R. Sandstroem, D. P. C. Sankey, A. Sansoni, C. Santoni, R. Santonico, H. Santos, I. Santoyo Castillo, K. Sapp, A. Sapronov, J. G. Saraiva, B. Sarrazin, G. Sartisohn, O. Sasaki, Y. Sasaki, G. Sauvage, E. Sauvan, P. Savard, D. O. Savu, C. Sawyer, L. Sawyer, D. H. Saxon, J. Saxon, C. Sbarra, A. Sbrizzi, T. Scanlon, D. A. Scannicchio, M. Scarcella, V. Scarfone, J. Schaarschmidt, P. Schacht, D. Schaefer, R. Schaefer, S. Schaepe, S. Schaetzel, U. Schäfer, A. C. Schaffer, D. Schaile, R. D. Schamberger, V. Scharf, V. A. Schegelsky, D. Scheirich, M. Schernau, M. I. Scherzer, C. Schiavi, J. Schieck, C. Schillo, M. Schioppa, S. Schlenker, E. Schmidt, K. Schmieden, C. Schmitt, S. Schmitt, B. Schneider, Y. J. Schnellbach, U. Schnoor, L. Schoeffel, A. Schoening, B. D. Schoenrock, A. L. S. Schorlemmer, M. Schott, D. Schouten, J. Schovancova, S. Schramm, M. Schreyer, C. Schroeder, N. Schuh, M. J. Schultens, H.-C. Schultz-Coulon, H. Schulz, M. Schumacher, B. A. Schumm, Ph. Schune, C. Schwanenberger, A. Schwartzman, T. A. Schwarz, Ph. Schwegler, Ph. Schwemling, R. Schwienhorst, J. Schwindling, T. Schwindt, M. Schwoerer, F. G. Sciacca, E. Scifo, G. Sciolla, W. G. Scott, F. Scuri, F. Scutti, J. Searcy, G. Sedov, E. Sedykh, S. C. Seidel, A. Seiden, F. Seifert, J. M. Seixas, G. Sekhniaidze, S. J. Sekula, K. E. Selbach, D. M. Seliverstov, G. Sellers, N. Semprini-Cesari, C. Serfon, L. Serin, L. Serkin, T. Serre, R. Seuster, H. Severini, T. Sfiligoj, F. Sforza, A. Sfyrla, E. Shabalina, M. Shamim, L. Y. Shan, R. Shang, J. T. Shank, M. Shapiro, P. B. Shatalov, K. Shaw, C. Y. Shehu, P. Sherwood, L. Shi, S. Shimizu, C. O. Shimmin, M. Shimojima, M. Shiyakova, A. Shmeleva, M. J. Shochet, D. Short, S. Shrestha, E. Shulga, M. A. Shupe, S. Shushkevich, P. Sicho, O. Sidiropoulou, D. Sidorov, A. Sidoti, F. Siegert, Dj. Sijacki, J. Silva, Y. Silver, D. Silverstein, S. B. Silverstein, V. Simak, O. Simard, Lj. Simic, S. Simion, E. Simioni, B. Simmons, R. Simoniello, M. Simonyan, P. Sinervo, N. B. Sinev, V. Sipica, G. Siragusa, A. Sircar, A. N. Sisakyan, S. Yu. Sivoklokov, J. Sjölin, T. B. Sjursen, H. P. Skottowe, K. Yu. Skovpen, P. Skubic, M. Slater, T. Slavicek, K. Sliwa, V. Smakhtin, B. H. Smart, L. Smestad, S. Yu. Smirnov, Y. Smirnov, L. N. Smirnova, O. Smirnova, K. M. Smith, M. Smizanska, K. Smolek, A. A. Snesarev, G. Snidero, S. Snyder, R. Sobie, F. Socher, A. Soffer, D. A. Soh, C. A. Solans, M. Solar, J. Solc, E. Yu. Soldatov, U. Soldevila, A. A. Solodkov, A. Soloshenko, O. V. Solovyanov, V. Solovyev, P. Sommer, H. Y. Song, N. Soni, A. Sood, A. Sopczak, B. Sopko, V. Sopko, V. Sorin, M. Sosebee, R. Soualah, P. Soueid, A. M. Soukharev, D. South, S. Spagnolo, F. Spanò, W. R. Spearman, F. Spettel, R. Spighi, G. Spigo, L. A. Spiller, M. Spousta, T. Spreitzer, B. Spurlock, R. D. St. Denis, S. Staerz, J. Stahlman, R. Stamen, S. Stamm, E. Stanecka, R. W. Stanek, C. Stanescu, M. Stanescu-Bellu, M. M. Stanitzki, S. Stapnes, E. A. Starchenko, J. Stark, P. Staroba, P. Starovoitov, R. Staszewski, P. Stavina, P. Steinberg, B. Stelzer, H. J. Stelzer, O. Stelzer-Chilton, H. Stenzel, S. Stern, G. A. Stewart, J. A. Stillings, M. C. Stockton, M. Stoebe, G. Stoicea, P. Stolte, S. Stonjek, A. R. Stradling, A. Straessner, M. E. Stramaglia, J. Strandberg, S. Strandberg, A. Strandlie, E. Strauss, M. Strauss, P. Strizenec, R. Ströhmer, D. M. Strom, R. Stroynowski, A. Struebig, S. A. Stucci, B. Stugu, N. A. Styles, D. Su, J. Su, R. Subramaniam, A. Succurro, Y. Sugaya, C. Suhr, M. Suk, V. V. Sulin, S. Sultansoy, T. Sumida, S. Sun, X. Sun, J. E. Sundermann, K. Suruliz, G. Susinno, M. R. Sutton, Y. Suzuki, M. Svatos, S. Swedish, M. Swiatlowski, I. Sykora, T. Sykora, D. Ta, C. Taccini, K. Tackmann, J. Taenzer, A. Taffard, R. Tafirout, N. Taiblum, H. Takai, R. Takashima, H. Takeda, T. Takeshita, Y. Takubo, M. Talby, A. A. Talyshev, J. Y. C. Tam, K. G. Tan, J. Tanaka, R. Tanaka, S. Tanaka, S. Tanaka, A. J. Tanasijczuk, B. B. Tannenwald, N. Tannoury, S. Tapprogge, S. Tarem, F. Tarrade, G. F. Tartarelli, P. Tas, M. Tasevsky, T. Tashiro, E. Tassi, A. Tavares Delgado, Y. Tayalati, F. E. Taylor, G. N. Taylor, W. Taylor, F. A. Teischinger, M. Teixeira Dias Castanheira, P. Teixeira-Dias, K. K. Temming, H. Ten Kate, P. K. Teng, J. J. Teoh, S. Terada, K. Terashi, J. Terron, S. Terzo, M. Testa, R. J. Teuscher, J. Therhaag, T. Theveneaux-Pelzer, J. P. Thomas, J. Thomas-Wilsker, E. N. Thompson, P. D. Thompson, P. D. Thompson, R. J. Thompson, A. S. Thompson, L. A. Thomsen, E. Thomson, M. Thomson, W. M. Thong, R. P. Thun, F. Tian, M. J. Tibbetts, V. O. Tikhomirov, Yu. A. Tikhonov, S. Timoshenko, E. Tiouchichine, P. Tipton, S. Tisserant, T. Todorov, S. Todorova-Nova, B. Toggerson, J. Tojo, S. Tokár, K. Tokushuku, K. Tollefson, E. Tolley, L. Tomlinson, M. Tomoto, L. Tompkins, K. Toms, N. D. Topilin, E. Torrence, H. Torres, E. Torró Pastor, J. Toth, F. Touchard, D. R. Tovey, H. L. Tran, T. Trefzger, L. Tremblet, A. Tricoli, I. M. Trigger, S. Trincaz-Duvoid, M. F. Tripiana, W. Trischuk, B. Trocmé, C. Troncon, M. Trottier-McDonald, M. Trovatelli, P. True, M. Trzebinski, A. Trzupek, C. Tsarouchas, J. C-L. Tseng, P. V. Tsiareshka, D. Tsionou, G. Tsipolitis, N. Tsirintanis, S. Tsiskaridze, V. Tsiskaridze, E. G. Tskhadadze, I. I. Tsukerman, V. Tsulaia, S. Tsuno, D. Tsybychev, A. Tudorache, V. Tudorache, A. N. Tuna, S. A. Tupputi, S. Turchikhin, D. Turecek, I. Turk Cakir, R. Turra, P. M. Tuts, A. Tykhonov, M. Tylmad, M. Tyndel, K. Uchida, I. Ueda, R. Ueno, M. Ughetto, M. Ugland, M. Uhlenbrock, F. Ukegawa, G. Unal, A. Undrus, G. Unel, F. C. Ungaro, Y. Unno, C. Unverdorben, D. Urbaniec, P. Urquijo, G. Usai, A. Usanova, L. Vacavant, V. Vacek, B. Vachon, N. Valencic, S. Valentinetti, A. Valero, L. Valery, S. Valkar, E. Valladolid Gallego, S. Vallecorsa, J. A. Valls Ferrer, W. Van Den Wollenberg, P. C. Van Der Deijl, R. van der Geer, H. van der Graaf, R. Van Der Leeuw, D. van der Ster, N. van Eldik, P. van Gemmeren, J. Van Nieuwkoop, I. van Vulpen, M. C. van Woerden, M. Vanadia, W. Vandelli, R. Vanguri, A. Vaniachine, P. Vankov, F. Vannucci, G. Vardanyan, R. Vari, E. W. Varnes, T. Varol, D. Varouchas, A. Vartapetian, K. E. Varvell, F. Vazeille, T. Vazquez Schroeder, J. Veatch, F. Veloso, S. Veneziano, A. Ventura, D. Ventura, M. Venturi, N. Venturi, A. Venturini, V. Vercesi, M. Verducci, W. Verkerke, J. C. Vermeulen, A. Vest, M. C. Vetterli, O. Viazlo, I. Vichou, T. Vickey, O. E. Vickey Boeriu, G. H. A. Viehhauser, S. Viel, R. Vigne, M. Villa, M. Villaplana Perez, E. Vilucchi, M. G. Vincter, V. B. Vinogradov, J. Virzi, I. Vivarelli, F. Vives Vaque, S. Vlachos, D. Vladoiu, M. Vlasak, A. Vogel, M. Vogel, P. Vokac, G. Volpi, M. Volpi, H. von der Schmitt, H. von Radziewski, E. von Toerne, V. Vorobel, K. Vorobev, M. Vos, R. Voss, J. H. Vossebeld, N. Vranjes, M. Vranjes Milosavljevic, V. Vrba, M. Vreeswijk, T. Vu Anh, R. Vuillermet, I. Vukotic, Z. Vykydal, P. Wagner, W. Wagner, H. Wahlberg, S. Wahrmund, J. Wakabayashi, J. Walder, R. Walker, W. Walkowiak, R. Wall, P. Waller, B. Walsh, C. Wang, C. Wang, F. Wang, H. Wang, H. Wang, J. Wang, J. Wang, K. Wang, R. Wang, S. M. Wang, T. Wang, X. Wang, C. Wanotayaroj, A. Warburton, C. P. Ward, D. R. Wardrope, M. Warsinsky, A. Washbrook, C. Wasicki, P. M. Watkins, A. T. Watson, I. J. Watson, M. F. Watson, G. Watts, S. Watts, B. M. Waugh, S. Webb, M. S. Weber, S. W. Weber, J. S. Webster, A. R. Weidberg, P. Weigell, B. Weinert, J. Weingarten, C. Weiser, H. Weits, P. S. Wells, T. Wenaus, D. Wendland, Z. Weng, T. Wengler, S. Wenig, N. Wermes, M. Werner, P. Werner, M. Wessels, J. Wetter, K. Whalen, A. White, M. J. White, R. White, S. White, D. Whiteson, D. Wicke, F. J. Wickens, W. Wiedenmann, M. Wielers, P. Wienemann, C. Wiglesworth, L. A. M. Wiik-Fuchs, P. A. Wijeratne, A. Wildauer, M. A. Wildt, H. G. Wilkens, J. Z. Will, H. H. Williams, S. Williams, C. Willis, S. Willocq, A. Wilson, J. A. Wilson, I. Wingerter-Seez, F. Winklmeier, B. T. Winter, M. Wittgen, T. Wittig, J. Wittkowski, S. J. Wollstadt, M. W. Wolter, H. Wolters, B. K. Wosiek, J. Wotschack, M. J. Woudstra, K. W. Wozniak, M. Wright, M. Wu, S. L. Wu, X. Wu, Y. Wu, E. Wulf, T. R. Wyatt, B. M. Wynne, S. Xella, M. Xiao, D. Xu, L. Xu, B. Yabsley, S. Yacoob, R. Yakabe, M. Yamada, H. Yamaguchi, Y. Yamaguchi, A. Yamamoto, K. Yamamoto, S. Yamamoto, T. Yamamura, T. Yamanaka, K. Yamauchi, Y. Yamazaki, Z. Yan, H. Yang, H. Yang, U. K. Yang, Y. Yang, S. Yanush, L. Yao, W-M. Yao, Y. Yasu, E. Yatsenko, K. H. Yau Wong, J. Ye, S. Ye, I. Yeletskikh, A. L. Yen, E. Yildirim, M. Yilmaz, R. Yoosoofmiya, K. Yorita, R. Yoshida, K. Yoshihara, C. Young, C. J. S. Young, S. Youssef, D. R. Yu, J. Yu, J. M. Yu, J. Yu, L. Yuan, A. Yurkewicz, I. Yusuff, B. Zabinski, R. Zaidan, A. M. Zaitsev, A. Zaman, S. Zambito, L. Zanello, D. Zanzi, C. Zeitnitz, M. Zeman, A. Zemla, K. Zengel, O. Zenin, T. Ženiš, D. Zerwas, G. Zevi della Porta, D. Zhang, F. Zhang, H. Zhang, J. Zhang, L. Zhang, X. Zhang, Z. Zhang, Z. Zhao, A. Zhemchugov, J. Zhong, B. Zhou, L. Zhou, N. Zhou, C. G. Zhu, H. Zhu, J. Zhu, Y. Zhu, X. Zhuang, K. Zhukov, A. Zibell, D. Zieminska, N. I. Zimine, C. Zimmermann, R. Zimmermann, S. Zimmermann, S. Zimmermann, Z. Zinonos, M. Ziolkowski, G. Zobernig, A. Zoccoli, M. zur Nedden, G. Zurzolo, V. Zutshi, L. Zwalinski

**Affiliations:** Department of Physics, University of Adelaide, Adelaide, Australia; Physics Department, SUNY Albany, Albany, NY USA; Department of Physics, University of Alberta, Edmonton, AB Canada; Department of Physics, Ankara University, Ankara, Turkey; LAPP, CNRS/IN2P3 and Université de Savoie, Annecy-le-Vieux, France; High Energy Physics Division, Argonne National Laboratory, Argonne, IL USA; Department of Physics, University of Arizona, Tucson, AZ USA; Department of Physics, The University of Texas at Arlington, Arlington, TX USA; Physics Department, University of Athens, Athens, Greece; Physics Department, National Technical University of Athens, Zografou, Greece; Institute of Physics, Azerbaijan Academy of Sciences, Baku, Azerbaijan; Institut de Física d’Altes Energies and Departament de Física de la Universitat Autònoma de Barcelona, Barcelona, Spain; Institute of Physics, University of Belgrade, Belgrade, Serbia; Department for Physics and Technology, University of Bergen, Bergen, Norway; Physics Division, Lawrence Berkeley National Laboratory and University of California, Berkeley, CA USA; Department of Physics, Humboldt University, Berlin, Germany; Albert Einstein Center for Fundamental Physics and Laboratory for High Energy Physics, University of Bern, Bern, Switzerland; School of Physics and Astronomy, University of Birmingham, Birmingham, UK; Department of Physics, Bogazici University, Istanbul, Turkey; INFN Sezione di Bologna, Bologna, Italy; Physikalisches Institut, University of Bonn, Bonn, Germany; Department of Physics, Boston University, Boston, MA USA; Department of Physics, Brandeis University, Waltham, MA USA; Universidade Federal do Rio De Janeiro COPPE/EE/IF, Rio de Janeiro, Brazil; Physics Department, Brookhaven National Laboratory, Upton, NY USA; National Institute of Physics and Nuclear Engineering, Bucharest, Romania; Departamento de Física, Universidad de Buenos Aires, Buenos Aires, Argentina; Cavendish Laboratory, University of Cambridge, Cambridge, UK; Department of Physics, Carleton University, Ottawa, ON Canada; CERN, Geneva, Switzerland; Enrico Fermi Institute, University of Chicago, Chicago, IL USA; Departamento de Física, Pontificia Universidad Católica de Chile, Santiago, Chile; Institute of High Energy Physics, Chinese Academy of Sciences, Beijing, China; Laboratoire de Physique Corpusculaire, Clermont Université and Université Blaise Pascal and CNRS/IN2P3, Clermont-Ferrand, France; Nevis Laboratory, Columbia University, Irvington, NY USA; Niels Bohr Institute, University of Copenhagen, Kobenhavn, Denmark; INFN Gruppo Collegato di Cosenza, Laboratori Nazionali di Frascati, Frascati, Italy; Faculty of Physics and Applied Computer Science, AGH University of Science and Technology, Kraków, Poland; The Henryk Niewodniczanski Institute of Nuclear Physics, Polish Academy of Sciences, Kraków, Poland; Physics Department, Southern Methodist University, Dallas, TX USA; Physics Department, University of Texas at Dallas, Richardson, TX USA; DESY, Hamburg and Zeuthen, Germany; Institut für Experimentelle Physik IV, Technische Universität Dortmund, Dortmund, Germany; Institut für Kern- und Teilchenphysik, Technische Universität Dresden, Dresden, Germany; Department of Physics, Duke University, Durham, NC USA; SUPA-School of Physics and Astronomy, University of Edinburgh, Edinburgh, UK; INFN Laboratori Nazionali di Frascati, Frascati, Italy; Fakultät für Mathematik und Physik, Albert-Ludwigs-Universität, Freiburg, Germany; Section de Physique, Université de Genève, Geneva, Switzerland; INFN Sezione di Genova, Genoa, Italy; E. Andronikashvili Institute of Physics, Iv. Javakhishvili Tbilisi State University, Tbilisi, Georgia; II Physikalisches Institut, Justus-Liebig-Universität Giessen, Giessen, Germany; SUPA-School of Physics and Astronomy, University of Glasgow, Glasgow, UK; II Physikalisches Institut, Georg-August-Universität, Göttingen, Germany; Laboratoire de Physique Subatomique et de Cosmologie, Université Grenoble-Alpes, CNRS/IN2P3, Grenoble, France; Department of Physics, Hampton University, Hampton, VA USA; Laboratory for Particle Physics and Cosmology, Harvard University, Cambridge, MA USA; Kirchhoff-Institut für Physik, Ruprecht-Karls-Universität Heidelberg, Heidelberg, Germany; Faculty of Applied Information Science, Hiroshima Institute of Technology, Hiroshima, Japan; Department of Physics, Indiana University, Bloomington, IN USA; Institut für Astro- und Teilchenphysik, Leopold-Franzens-Universität, Innsbruck, Austria; University of Iowa, Iowa City, IA USA; Department of Physics and Astronomy, Iowa State University, Ames, IA USA; Joint Institute for Nuclear Research, JINR Dubna, Dubna, Russia; KEK, High Energy Accelerator Research Organization, Tsukuba, Japan; Graduate School of Science, Kobe University, Kobe, Japan; Faculty of Science, Kyoto University, Kyoto, Japan; Kyoto University of Education, Kyoto, Japan; Department of Physics, Kyushu University, Fukuoka, Japan; Instituto de Física La Plata, Universidad Nacional de La Plata and CONICET, La Plata, Argentina; Physics Department, Lancaster University, Lancaster, UK; INFN Sezione di Lecce, Lecce, Italy; Oliver Lodge Laboratory, University of Liverpool, Liverpool, UK; Department of Physics, Jožef Stefan Institute and University of Ljubljana, Ljubljana, Slovenia; School of Physics and Astronomy, Queen Mary University of London, London, UK; Department of Physics, Royal Holloway University of London, Surrey, UK; Department of Physics and Astronomy, University College London, London, UK; Louisiana Tech University, Ruston, LA USA; Laboratoire de Physique Nucléaire et de Hautes Energies, UPMC and Université Paris-Diderot and CNRS/IN2P3, Paris, France; Fysiska institutionen, Lunds universitet, Lund, Sweden; Departamento de Fisica Teorica C-15, Universidad Autonoma de Madrid, Madrid, Spain; Institut für Physik, Universität Mainz, Mainz, Germany; School of Physics and Astronomy, University of Manchester, Manchester, UK; CPPM, Aix-Marseille Université and CNRS/IN2P3, Marseille, France; Department of Physics, University of Massachusetts, Amherst, MA USA; Department of Physics, McGill University, Montreal, QC Canada; School of Physics, University of Melbourne, Melbourne, VIC Australia; Department of Physics, The University of Michigan, Ann Arbor, MI USA; Department of Physics and Astronomy, Michigan State University, East Lansing, MI USA; INFN Sezione di Milano, Milan, Italy; B.I. Stepanov Institute of Physics, National Academy of Sciences of Belarus, Minsk, Republic of Belarus; National Scientific and Educational Centre for Particle and High Energy Physics, Minsk, Republic of Belarus; Department of Physics, Massachusetts Institute of Technology, Cambridge, MA USA; Group of Particle Physics, University of Montreal, Montreal, QC Canada; P.N. Lebedev Institute of Physics, Academy of Sciences, Moscow, Russia; Institute for Theoretical and Experimental Physics (ITEP), Moscow, Russia; Moscow Engineering and Physics Institute (MEPhI), Moscow, Russia; D.V. Skobeltsyn Institute of Nuclear Physics, M.V. Lomonosov Moscow State University, Moscow, Russia; Fakultät für Physik, Ludwig-Maximilians-Universität München, Munich, Germany; Max-Planck-Institut für Physik (Werner-Heisenberg-Institut), Munich, Germany; Nagasaki Institute of Applied Science, Nagasaki, Japan; Graduate School of Science and Kobayashi-Maskawa Institute, Nagoya University, Nagoya, Japan; INFN Sezione di Napoli, Naples, Italy; Department of Physics and Astronomy, University of New Mexico, Albuquerque, NM USA; Institute for Mathematics, Astrophysics and Particle Physics, Radboud University Nijmegen/Nikhef, Nijmegen, The Netherlands; Nikhef National Institute for Subatomic Physics and University of Amsterdam, Amsterdam, The Netherlands; Department of Physics, Northern Illinois University, DeKalb, IL USA; Budker Institute of Nuclear Physics, SB RAS, Novosibirsk, Russia; Department of Physics, New York University, New York, NY USA; Ohio State University, Columbus, OH USA; Faculty of Science, Okayama University, Okayama, Japan; Homer L. Dodge Department of Physics and Astronomy, University of Oklahoma, Norman, OK USA; Department of Physics, Oklahoma State University, Stillwater, OK USA; Palacký University, RCPTM, Olomouc, Czech Republic; Center for High Energy Physics, University of Oregon, Eugene, OR USA; LAL, Université Paris-Sud and CNRS/IN2P3, Orsay, France; Graduate School of Science, Osaka University, Osaka, Japan; Department of Physics, University of Oslo, Oslo, Norway; Department of Physics, Oxford University, Oxford, UK; INFN Sezione di Pavia, Pavia, Italy; Department of Physics, University of Pennsylvania, Philadelphia, PA USA; Petersburg Nuclear Physics Institute, Gatchina, Russia; INFN Sezione di Pisa, Pisa, Italy; Department of Physics and Astronomy, University of Pittsburgh, Pittsburgh, PA USA; Laboratorio de Instrumentacao e Fisica Experimental de Particulas-LIP, Lisbon, Portugal; Institute of Physics, Academy of Sciences of the Czech Republic, Praha, Czech Republic; Czech Technical University in Prague, Praha, Czech Republic; Faculty of Mathematics and Physics, Charles University in Prague, Prague, Czech Republic; State Research Center Institute for High Energy Physics, Protvino, Russia; Particle Physics Department, Rutherford Appleton Laboratory, Didcot, UK; Physics Department, University of Regina, Regina, SK Canada; Ritsumeikan University, Kusatsu, Shiga Japan; INFN Sezione di Roma, Rome, Italy; INFN Sezione di Roma Tor Vergata, Rome, Italy; INFN Sezione di Roma Tre, Rome, Italy; Faculté des Sciences Ain Chock, Réseau Universitaire de Physique des Hautes Energies-Université Hassan II, Casablanca, Morocco; DSM/IRFU (Institut de Recherches sur les Lois Fondamentales de l’Univers), CEA Saclay (Commissariat à l’Energie Atomique et aux Energies Alternatives), Gif-sur-Yvette, France; Santa Cruz Institute for Particle Physics, University of California Santa Cruz, Santa Cruz, CA USA; Department of Physics, University of Washington, Seattle, WA USA; Department of Physics and Astronomy, University of Sheffield, Sheffield, UK; Department of Physics, Shinshu University, Nagano, Japan; Fachbereich Physik, Universität Siegen, Siegen, Germany; Department of Physics, Simon Fraser University, Burnaby, BC Canada; SLAC National Accelerator Laboratory, Stanford, CA USA; Faculty of Mathematics, Physics and Informatics, Comenius University, Bratislava, Slovak Republic; Department of Physics, University of Cape Town, Cape Town, South Africa; Department of Physics, Stockholm University, Stockholm, Sweden; Physics Department, Royal Institute of Technology, Stockholm, Sweden; Departments of Physics and Astronomy and Chemistry, Stony Brook University, Stony Brook, NY USA; Department of Physics and Astronomy, University of Sussex, Brighton, UK; School of Physics, University of Sydney, Sydney, Australia; Institute of Physics, Academia Sinica, Taipei, Taiwan; Department of Physics, Technion: Israel Institute of Technology, Haifa, Israel; Raymond and Beverly Sackler School of Physics and Astronomy, Tel Aviv University, Tel Aviv, Israel; Department of Physics, Aristotle University of Thessaloniki, Thessaloniki, Greece; International Center for Elementary Particle Physics and Department of Physics, The University of Tokyo, Tokyo, Japan; Graduate School of Science and Technology, Tokyo Metropolitan University, Tokyo, Japan; Department of Physics, Tokyo Institute of Technology, Tokyo, Japan; Department of Physics, University of Toronto, Toronto, ON Canada; TRIUMF, Vancouver, BC Canada; Faculty of Pure and Applied Sciences, University of Tsukuba, Tsukuba, Japan; Department of Physics and Astronomy, Tufts University, Medford, MA USA; Centro de Investigaciones, Universidad Antonio Narino, Bogota, Colombia; Department of Physics and Astronomy, University of California Irvine, Irvine, CA USA; INFN Gruppo Collegato di Udine, Sezione di Trieste, Udine, Italy; ICTP, Trieste, Italy; Dipartimento di Chimica, Fisica e Ambiente, Università di Udine, Udine, Italy; Department of Physics, University of Illinois, Urbana, IL USA; Department of Physics and Astronomy, University of Uppsala, Uppsala, Sweden; Instituto de Física Corpuscular (IFIC) and Departamento de Física Atómica, Molecular y Nuclear and Departamento de Ingeniería Electrónica and Instituto de Microelectrónica de Barcelona (IMB-CNM), University of Valencia and CSIC, Valencia, Spain; Department of Physics, University of British Columbia, Vancouver, BC Canada; Department of Physics and Astronomy, University of Victoria, Victoria, BC Canada; Department of Physics, University of Warwick, Coventry, UK; Waseda University, Tokyo, Japan; Department of Particle Physics, The Weizmann Institute of Science, Rehovot, Israel; Department of Physics, University of Wisconsin, Madison, WI USA; Fakultät für Physik und Astronomie, Julius-Maximilians-Universität, Würzburg, Germany; Fachbereich C Physik, Bergische Universität Wuppertal, Wuppertal, Germany; Department of Physics, Yale University, New Haven, CT USA; Yerevan Physics Institute, Yerevan, Armenia; Centre de Calcul de l’Institut National de Physique Nucléaire et de Physique des Particules (IN2P3), Villeurbanne, France; CERN, 1211 Geneva 23, Switzerland; Department of Physics, Gazi University, Ankara, Turkey; Division of Physics, TOBB University of Economics and Technology, Ankara, Turkey; Turkish Atomic Energy Authority, Ankara, Turkey; Vinca Institute of Nuclear Sciences, University of Belgrade, Belgrade, Serbia; Department of Physics, Dogus University, Istanbul, Turkey; Department of Physics Engineering, Gaziantep University, Gaziantep, Turkey; Dipartimento di Fisica e Astronomia, Università di Bologna, Bologna, Italy; Federal University of Juiz de Fora (UFJF), Juiz de Fora, Brazil; Federal University of Sao Joao del Rei (UFSJ), Sao Joao del, Brazil; Instituto de Fisica, Universidade de Sao Paulo, São Paulo, Brazil; Physics Department, National Institute for Research and Development of Isotopic and Molecular Technologies, Cluj Napoca, Romania; University Politehnica Bucharest, Bucharest, Romania; West University in Timisoara, Timisoara, Romania; Departamento de Física, Universidad Técnica Federico Santa María, Valparaiso, Chile; Department of Modern Physics, University of Science and Technology of China, Anhui, China; Department of Physics, Nanjing University, Jiangsu, China; School of Physics, Shandong University, Shandong, China; Physics Department, Shanghai Jiao Tong University, Shanghai, China; Dipartimento di Fisica, Università della Calabria, Rende, Italy; Marian Smoluchowski Institute of Physics, Jagiellonian University, Kraków, Poland; Dipartimento di Fisica, Università di Genova, Genoa, Italy; High Energy Physics Institute, Tbilisi State University, Tbilisi, Georgia; Physikalisches Institut, Ruprecht-Karls-Universität Heidelberg, Heidelberg, Germany; ZITI Institut für technische Informatik, Ruprecht-Karls-Universität Heidelberg, Mannheim, Germany; Dipartimento di Matematica e Fisica, Università del Salento, Lecce, Italy; Dipartimento di Fisica, Università di Milano, Milan, Italy; Dipartimento di Fisica, Università di Napoli, Naples, Italy; Dipartimento di Fisica, Università di Pavia, Pavia, Italy; Dipartimento di Fisica E. Fermi, Università di Pisa, Pisa, Italy; Faculdade de Ciências, Universidade de Lisboa, Lisbon, Portugal; Department of Physics, University of Coimbra, Coimbra, Portugal; Centro de Física Nuclear da Universidade de Lisboa, Lisbon, Portugal; Departamento de Fisica, Universidade do Minho, Braga, Portugal; Departamento de Fisica Teorica y del Cosmos and CAFPE, Universidad de Granada, Granada, Spain; Departamento de Fisica and CEFITEC of Faculdade de Ciencias e Tecnologia, Universidad de Granada, Granada, Spain; Dipartimento di Fisica, Sapienza Università di Roma, Rome, Italy; Dipartimento di Fisica, Università di Roma Tor Vergata, Rome, Italy; Dipartimento di Matematica e Fisica, Università Roma Tre, Rome, Italy; Centre National de l’Energie des Sciences Techniques Nucleaires, Rabat, Morocco; Faculté des Sciences Semlalia, Université Cadi Ayyad, LPHEA-Marrakech, Marrakech, Morocco; Faculté des Sciences, Université Mohamed Premier and LPTPM, Oujda, Morocco; Faculté des Sciences, Université Mohammed V-Agdal, Rabat, Morocco; Department of Subnuclear Physics, Institute of Experimental Physics of the Slovak Academy of Sciences, Kosice, Slovak Republic; Department of Physics, University of Johannesburg, Johannesburg, South Africa; School of Physics, University of the Witwatersrand, Johannesburg, South Africa; The Oskar Klein Centre, Stockholm, Sweden; Department of Physics and Astronomy, York University, Toronto, ON Canada; ICTP, Trieste, Italy; Dipartimento di Chimica, Fisica e Ambiente, Università di Udine, Trieste, Italy

## Abstract

Double-differential three-jet production cross-sections are measured in proton–proton collisions at a centre-of-mass energy of $\sqrt{s} = 7\mathrm \,TeV{}$ using the ATLAS detector at the large hadron collider. The measurements are presented as a function of the three-jet mass $(m_{jjj})$, in bins of the sum of the absolute rapidity separations between the three leading jets $(\left| Y^{*}\right| )$. Invariant masses extending up to 5  TeV are reached for $8< \left| Y^{*}\right| < 10$. These measurements use a sample of data recorded using the ATLAS detector in 2011, which corresponds to an integrated luminosity of $4.51~\text{ fb }^{-1}$. Jets are identified using the anti-$k_{t}$ algorithm with two different jet radius parameters, $R=0.4$ and $R=0.6$. The dominant uncertainty in these measurements comes from the jet energy scale. Next-to-leading-order QCD calculations corrected to account for non-perturbative effects are compared to the measurements. Good agreement is found between the data and the theoretical predictions based on most of the available sets of parton distribution functions, over the full kinematic range, covering almost seven orders of magnitude in the measured cross-section values.

## Introduction

Collimated jets of hadrons are a characteristic feature of high-energy particle interactions. In the theory of strong interactions, quantum chromodynamics (QCD), jets can be interpreted as the result of fragmentation of partons produced in a scattering process. In high-energy particle collisions two main phases can be distinguished. In the perturbative phase, partons with high-transverse momentum ($p_{\mathrm {T}}$) are produced in a hard-scattering process at a scale $Q$. This phase is described by a perturbative expansion in QCD. In the transition to the second (non-perturbative) phase, these partons emit additional gluons and produce quark–antiquark pairs. The non-perturbative jet evolution is an interplay between the hadronisation process and the underlying event. The hadronisation process governs the transition from partons to hadrons and the underlying event represents initial-state radiation, multiple parton interactions and colour-reconnection effects [1]. In spite of these phenomena, the highly collimated sprays of particles, collectively identified as hadron jets, are observed in the final state. The effects of both hadronisation and the underlying event vary strongly with the jet radius parameter and are most pronounced at low $p_{\mathrm {T}}$. They are accounted for using phenomenological models that are tuned to the data.

The ATLAS Collaboration has measured the inclusive jet cross-sections at $$7$$  TeV [2] and at $$2.76$$  TeV [3] centre-of-mass energies in $$pp$$ collisions for jets defined by the anti-$$k_{t}$$ algorithm [4] with two jet radius parameters, $$R=0.4$$ and $$R=0.6$$. Recent inclusive jet [5] and dijet [6] cross-section measurements at $$7$$  TeV centre-of-mass energy in $$pp$$ collisions have exploited improved jet energy calibration procedures [7] leading to smaller systematic uncertainties compared to those achieved in Refs. [2, 3]. Similar measurements at $$7$$  TeV centre-of-mass energy in $$pp$$ collisions [8, 9] have been carried out by the CMS Collaboration. These measurements test perturbative QCD (pQCD) at very short distances and have provided constraints on the gluon momentum distribution within protons at large momentum fraction. The impact of higher order effects on the inclusive jet cross-section ratios of anti-$$k_{t}$$$$R=0.5$$ and $$R=0.7$$ jets has been studied in [10]. The inclusive three-jet to two-jet ratio [11] is used to determine the strong coupling constant. Theoretical predictions of the multi-jet cross-sections in $$pp$$ collisions at $$7$$  TeV centre-of-mass energy have been tested in Refs. [12, 13].

Previous measurements of three-jet cross-sections in $p\bar{p}$ collisions were performed by the $\text {D}{\emptyset }$ collaboration [14]. The measurements were compared to predictions, and agreement between data and theory was found within the uncertainties.

In this paper, measurements of double-differential three-jet production cross-sections are presented as a function of the three-jet mass ($m_{jjj}$) and the sum of absolute rapidity separation between the three leading jets ($\left| Y^{*}\right|$). The measurements are corrected for experimental effects and reported at the particle level. The three-jet mass distributions test the dynamics of the underlying $2\rightarrow 3$ scattering process. The distributions are sensitive to both the transverse momentum ($p_{\mathrm {T}}$) spectra of the three leading jets and their angular correlations, since a massive three-jet system can be built either from high-$p_{\mathrm {T}}$ jets or from jets with large rapidity separation. Binning in $\left| Y^{*}\right|$ allows events with $m_{jjj}$ originating from these different regions of phase space to be separated.

The analysis presented in this paper tests the description of multi-jet events in next-to-leading-order

(NLO) QCD and uses two different values of jet radius parameter, $R=0.4$ and $R=0.6$, since three-jet cross-sections depend on the jet radius even at leading order (LO) in the perturbative expansion. The NLO QCD calculations corrected to account for non-perturbative effects are compared to the measured cross-sections. The measurements also provide constraints on the proton’s parton distribution functions (PDFs) beyond those from inclusive and dijet cross-sections, since they probe a different region of phase space in proton momentum fraction and squared momentum transfer $(x,Q^2)$ and different combinations of initial-state partons.

The content of this paper is structured as follows. The ATLAS detector is briefly described in Sect. 2, followed by the definition of observables and description of Monte Carlo (MC) samples in Sects. 3 and 4, respectively. The trigger, data selection and jet calibration are presented in Sect. 5. Data unfolding and experimental uncertainties are described in Sects. 6 and 7. Section 8 describes the theoretical predictions for the measurements in this paper. The cross-section results are presented in Sect. 9 and the conclusions are given in Sect. 10.

## The ATLAS experiment

The ATLAS detector is described in detail in Ref. [15]. ATLAS uses a right-handed coordinate system with its origin at the nominal interaction point (IP) in the centre of the detector and the $z$-axis pointing along the beam axis. The $x$-axis points from the IP to the centre of the LHC ring, and the $y$-axis points upward. Cylindrical coordinates ($r$, $\phi$) are used in the transverse plane, $\phi$ being the azimuthal angle around the beam pipe. The pseudorapidity is defined in terms of the polar angle $\theta$ as $\eta =-\ln \tan (\theta /2)$. The rapidity is defined in terms of the energy $E$ and longitudinal to the beam pipe momentum $p_z$ as $y=1/2\ln {\left( (E+p_z)/(E-p_z)\right) }$. The transverse momentum $p_{\mathrm {T}}$ is defined as the component of the momentum transverse to the beam pipe.

The inner detector (ID) is used to measure the momenta and trajectories of charged particles. The ID has full coverage in the azimuthal angle $\phi$ and over the pseudorapidity range $|\eta |<2.5$. The ID is immersed in a 2 T magnetic field provided by a superconducting solenoid magnet.

The main detector system used for this analysis is the calorimeter. The electromagnetic calorimeters use liquid argon (LAr) as the active detector medium. They employ accordion-shaped electrodes and lead absorbers, and are divided into one barrel ($|\eta |<1.475$) and two end-cap components ($1.375<|\eta |<3.2$). The technology used for the hadronic calorimeters depends on $\eta$. In the barrel region ($|\eta |<1.7$), the detector is made of scintillator tiles with steel absorbers. In the end-cap region ($1.5<|\eta |<3.2$), the detector uses LAr and copper. A forward calorimeter consisting of LAr and tungsten/copper absorbers has both electromagnetic and hadronic sections, and extends the coverage to $|\eta | = 4.9$.

The muon spectrometer has one barrel and two end-cap air-core toroid magnets. Three layers of precision tracking stations provide muon momentum measurements over the range $|\eta |<2.7$.

The ATLAS trigger system consists of three levels of event selection: a first level implemented using custom-made electronics, which selects events at a design rate of at most 75 kHz, followed by two successive software-based levels. The level-2 trigger uses fast online algorithms, and the final trigger stage, event filter (EF), uses reconstruction software with algorithms similar to the offline versions.

## Cross-section definition

Jets are defined using the anti-$k_{t}$ algorithm as implemented in the FastJet [16] package, with two different values of the radius parameter: $R=0.4$ and $R=0.6$.

Events containing at least three jets within the rapidity range $|y|<3.0$ with $p_{\mathrm {T}}>50$  GeV are considered. The leading, subleading and sub-subleading jets are required to have $p_{\mathrm {T}}> 150$  GeV, $p_{\mathrm {T}}> 100$  GeV and $p_{\mathrm {T}}> 50$  GeV, respectively.

Three-jet double-differential cross-sections are measured as a function of the three-jet mass

$$\begin{aligned} m_{jjj}{}=\sqrt{\left( p_1+p_2+p_3\right) ^2} \end{aligned}$$

and the summed absolute rapidity separation of the three leading jets

$$\begin{aligned} \left| Y^{*}\right| = \left| y_1 - y_2\right| + \left| y_2 - y_3\right| + \left| y_1 - y_3\right| , \end{aligned}$$

where $p_i(y_i)$ are the four-momenta (rapidities) of the three leading jets. The measurements are made in five ranges of $\left| Y^{*}\right| < 10$, in equal steps of two. In each range of $\left| Y^{*}\right|$, a lower limit on the three-jet mass is imposed to avoid the region of phase space affected by the jet $p_{\mathrm {T}}$ cuts. The measurement starts at $m_{jjj}=380$  GeV in the $\left| Y^{*}\right| {}<2$ bin, increasing to $1180$  GeV for the $8<\left| Y^{*}\right| <10$ bin.

The three-jet mass distributions are corrected for detector effects, and the measured cross-sections are defined at the particle level. Here particle level refers to jets built using produced particles with a proper lifetime longer than $10~\text{ ps }$, including muons and neutrinos from decaying hadrons [17].

## Monte Carlo samples

The default MC generator used to simulate events is Pythia 6 [18] with the Perugia 2011 tune [19] and the CTEQ5L PDFs [20]. Usually, “tune“ refers to a set of model parameters, which provide an optimal description of high-energy particle collisions. Data from previous colliders (LEP, TEVATRON, etc), as well as early LHC data are included in the process of tuning the model parameters [19, 21, 22]. The Pythia 6 is a generator with LO $$2 \rightarrow 2$$ matrix element calculations, supplemented by leading-logarithmic calculations of parton showers ordered in $$p_{\mathrm {T}}$$. A simulation of the underlying event, including multiple parton interactions, is also included. The Lund string model [23, 24] is used to simulate the fragmentation process. The signal reconstruction is affected by multiple proton–proton interactions occurring during the same bunch crossing and by remnants of electronic signals from previous bunch crossings in the detectors (pileup). To simulate pileup, inelastic $$pp$$ events are generated using Pythia 8 [25] with the 4C tune [26] and MRST LO$$^{**}$$ proton PDF set [27]. The number of minimum-bias events overlaid on each signal event is chosen to reproduce the distribution of the average number of simultaneous $$pp$$ collisions $$\langle \mu \rangle $$ in an event. During the 2011 data-taking period $$\langle \mu \rangle $$ changed from 5 to 18 with increasing instantaneous luminosity.

To estimate the uncertainties in the modelling of the hard scattering, hadronisation, the underlying event and of parton showers, events are also simulated using Alpgen [28], a multi-leg LO MC simulation, with up to six final-state partons in the matrix element calculations, interfaced to Herwig 6.5.10 [29–31] using the AUET2 tune [21] with the CTEQ6L1 PDF set [32] for parton showers and Jimmy 4.31 [33] for the underlying event.

The outputs from these event generators are passed to the detector simulation [34], based on Geant4 [35]. Simulated events are digitised [36, 37] to model the detector responses, and then reconstructed using the same software as used to process the data.

## Data selection and jet calibration

This analysis is based on data collected with the ATLAS detector in the year 2011 during periods with stable $pp$ collisions at $\sqrt{s}=7~\mathrm \,TeV$ in which all relevant detector components were operational. The resulting data sample corresponds to an integrated luminosity of $4.51\pm 0.08~\text{ fb }^{-1}$ [38].

The presence of at least one primary vertex (compatible with the position of the beam spot), reconstructed using two or more tracks with $p_{\mathrm {T}}>500$  MeV, is required to reject cosmic ray events and beam-related backgrounds. The primary vertex with the largest sum of squared transverse momenta of associated tracks is used as the interaction point for the analysis.

Due to the high instantaneous luminosity and a limited detector readout bandwidth, a set of single-jet triggers with increasing transverse energy ($E_{\mathrm {T}}$) thresholds is used to collect data events with jets. Only a fraction of the events that fired the trigger are actually recorded. The reciprocal of this fraction is the prescale factor of the trigger considered. The triggers with lower $E_{\mathrm {T}}$ thresholds were prescaled with higher factors and only the trigger with the highest $E_{\mathrm {T}}$ threshold remained unprescaled during the whole data-taking period. The prescale factors are adjusted to keep the jet yield approximately constant as a function of $E_{\mathrm {T}}$.

An event must pass all three levels of the jet trigger system. The trigger is based on the $E_{\mathrm {T}}$ of jet-like objects. Level-1 provides a fast hardware decision based on the summed $E_{\mathrm {T}}$ of calorimeter towers using a sliding-window algorithm. Level-2 performs a simple jet reconstruction in a geometric region around the object that fired the Level-1 trigger. Finally, a full jet reconstruction using the anti-$k_{t}$ algorithm with $R=0.4$ is performed over the entire detector by the third level trigger.

The trigger efficiencies are determined as a function of $m_{jjj}$ in each bin of $\left| Y^{*}\right|$ separately for $R=0.4$ and $R=0.6$ jet radius parameters. They are evaluated using an unbiased sample of events that fired the jet trigger with a $p_{\mathrm {T}}=30$  GeV threshold at the EF level. This trigger is fully efficient in events with a leading jet passing the three-jet analysis requirements. For every $\left| Y^{*}\right|$ bin, the full range of three-jet mass is divided into subranges, each filled by only one of the several single-jet triggers. Triggers are used only where the trigger efficiency is above $99\,\%$. Moreover, the lower $m_{jjj}$ bound for each trigger is shifted up by $15\,\%$ from the $99\,\%$ efficiency point to avoid any possible biases from the trigger strategy chosen for this measurement. This shift leads to a negligible increase in the statistical error on the measured cross-sections, compared to the total uncertainty.

Since the EF reconstructs jets with a radius parameter $$R=0.4$$, the $$p_{\mathrm {T}}$$ threshold at which the trigger for jets defined with $$R=0.6$$ becomes fully efficient is significantly higher than for $$R=0.4$$ jets. Using the same trigger subranges for both jet sizes would reduce the number of events with anti-$$k_{t}$$$$R=0.4$$ jets. To take advantage of the lower $$p_{\mathrm {T}}$$ at which triggers are fully efficient for $$R=0.4$$ jets, different assignments between triggers and $$m_{jjj}$$ ranges are considered for these jets and jets reconstructed with $$R=0.6$$.

After events are selected by the trigger system, they are fully reconstructed offline. The input objects to the jet algorithm are three-dimensional topo-clusters [39]. Each topo-cluster is constructed from a seed calorimeter cell with energy $|E_\mathrm{cell}| > 4\sigma$, where $\sigma$ is the width of the total noise distribution of the cell from both the electronics and pileup sources. Neighbouring cells are added to the topo-cluster if they have $|E_\mathrm{cell}| > 2\sigma$. At the last step, all neighbouring cells are added. A local hadronic calibration (LC) that accounts for inactive material, out-of-cluster losses for pions, and calorimeter response is applied to clusters identified as hadronic by their energy density distribution [40]. The LC improves the topo-cluster energy resolution, and the jet clustering algorithm propagates this improvement to the jet level. The LC is validated using single pions in the combined test-beam [40].

Each topo-cluster is considered as a massless particle with an energy $E=\sum E_\mathrm{cell}$, and a direction given by the energy-weighted barycentre of the cells in the cluster with respect to the geometrical centre of the ATLAS detector. The four-momentum of an uncalibrated jet is defined as the sum of the four-momenta of the clusters making up the jet. The jet is then calibrated in four steps:

1. An estimated mean additional energy due to pileup is subtracted using a correction derived from MC simulation and validated in situ using track-jets in dijet events and photons in $\gamma$-jet events as a function of the average number of $pp$ collisions in the same bunch crossing, $\langle \mu \rangle$, the number of primary vertices, $N_{\mathrm{PV}}$, and jet $\eta$ [41]. Here, track-jets are reconstructed from all tracks associated to the primary vertex using the anti-$k_{t}$ jet algorithm.
2. The direction of the jet is corrected such that the jet originates from the selected hard-scatter vertex of the event instead of the geometrical centre of ATLAS.
3. The energy and the position of the jet are corrected for instrumental effects (calorimeter non-compensation, additional inactive material, effects due to the magnetic field) using correction factors obtained from MC simulation. The jet energy scale is restored on average to that of the particle-level jet. For the calibration, the particle-level jet does not include muons and non-interacting particles.
4. An additional in situ calibration is applied to correct for residual differences between the MC simulation and data, derived by combining the results of dijet, $\gamma$-jet, $Z$-jet, and multi-jet momentum balance techniques.

The full calibration procedure is described in detail in Ref. [7].

Data-taking in the year 2011 was affected by a read-out problem in a region of the LAr calorimeter, causing jets in this region to be poorly reconstructed. In order to avoid a bias in the spectra, events with any of the three leading jets falling in the region $-0.88 < \phi < -0.5$ were rejected. Approximately $15\,\%$ of events are removed by this requirement. This inefficiency is corrected for using MC simulation (cf. Sect. 6).

The three leading jets are required to satisfy the “medium” quality criteria as described in Ref. [42], designed to reject cosmic-rays, beam-halo particles, and detector noise. More than $$5.3(2.5)\times 10^6$$ three-jet events are selected with radius parameter $$R=0.4$$$$(0.6)$$.

## Data unfolding

The three-jet cross-sections as a function of $m_{jjj}$ are obtained by unfolding the data distributions, and correcting for detector resolutions and inefficiencies. This procedure includes a correction for the undetected presence of muons and neutrinos from hadron decays in jets. The unfolding procedure is based on the iterative, dynamically stabilised (IDS) unfolding method [43]. Further details can be found in Ref. [2]. To account for bin-to-bin migrations, a transfer matrix is built from the MC simulation, relating the particle-level and reconstruction-level three-jet masses. The reconstruction-level to particle-level event association is done in the $m_{jjj}$–$\left| Y^{*}\right|$ plane, such that only a requirement on the presence of a three-jet system is made. Since bin-to-bin migrations are usually due to jet energy smearing of the three-jet mass, and less often due to jet angular resolution, the migrations across $\left| Y^{*}\right|$ bins are negligible and the unfolding is performed separately in each $\left| Y^{*}\right|$ bin.

The data are unfolded to the particle level using a three-step procedure

1 $$\begin{aligned} N_i^\mathcal{P} = \frac{1}{\epsilon ^\mathcal{P}_i} \sum _{(j)} N_j^\mathcal{R} \cdot \epsilon _j^\mathcal{R}A_{ij}, \end{aligned}$$

where $i$ ($j$) is the particle-level (reconstruction-level) bin index, and $N_i^\mathcal{P}$ ($N_i^\mathcal{R}$) is the number of particle-level (reconstruction-level) events in bin $i$. The quantities $\epsilon _i^\mathcal{R}$ ($\epsilon _i^\mathcal{P}$) are the fractions of reconstruction-level (particle-level) events matching (associated with) particle-level (reconstruction-level) events in each bin $i$. These efficiencies are used to correct for the matching inefficiency at the reconstruction and particle level, respectively. The element $A_{ij}$ of the transfer matrix is the probability for a reconstruction-level event in bin $j$ to be associated with a particle-level event in bin $i$. It is used to unfold the reconstruction-level spectrum for detector effects.

A data-driven closure test is used to evaluate the bias in the unfolded data spectrum shape due to mis-modelling of the reconstruction-level spectrum shape in the MC simulation. The transfer matrix is improved through a series of iterations, where the particle-level distribution from simulation is re-weighted such that the reconstruction-level distribution from simulation matches the data distribution. The modified reconstruction-level MC simulation is unfolded using the original transfer matrix, and the result is compared with the modified particle-level spectrum. The resulting bias is considered as a systematic uncertainty. For the analyses in this paper, one iteration is used, which leads to a bias in closure tests of less than one percent.

The statistical uncertainties in the unfolded results are estimated using pseudo-experiments. Each event in the data and in the MC simulation is counted $n$ times, where $n$ is sampled from a Poisson distribution with a mean of one. A fluctuated transfer matrix and efficiency corrections are calculated as the average over these pseudo-experiments in MC simulation. Then, each resulting pseudo-experiment of the data spectrum is unfolded using the fluctuated transfer matrix and efficiency corrections. Finally, the covariance matrix between bins of measured $m_{jjj}$ cross-section is calculated using the set of unfolded pseudo-experiments of the data. The random numbers for the pseudo-experiments are generated using unique seeds. The dijet [6] and inclusive jet [5] cross-section measurements use the same unique seeds to evaluate the statistical uncertainties. In this way, the statistical uncertainty and bin-to-bin correlations in both the data and the MC simulation are encoded in the covariance matrix and the statistical correlation between different measurements can be taken into account in combined fits.

## Experimental uncertainties

The uncertainty in the jet energy scale (JES) calibration is the dominant uncertainty in this measurement. The uncertainties in the central region are determined using a combination of the transverse momentum balance techniques, such as $Z$-jet, $\gamma$-jet and multi-jet balance measurements performed in situ. In each of the methods, the uncertainties in the energy of the well-measured objects, e.g. $Z$/photon or system of low-$p_{\mathrm {T}}$ jets, are propagated to the energy of the balancing jet. The JES uncertainty in the central region is propagated to the forward region using transverse momentum balance between a central and a forward jet in events with two jets. The difference in the balance observed between MC simulation samples generated with Pythia and Herwig is treated as an additional uncertainty in the forward region. The JES uncertainty in the high-$p_{\mathrm {T}}$ range is evaluated using the in situ measurement of the single isolated hadron response [44]. The total JES uncertainty is described by the set of fully correlated in $p_{\mathrm {T}}$ independent uncertainty sources. Complete details of the JES derivation and its uncertainties can be found in Ref. [7].

The uncertainty in the $p_{\mathrm {T}}$ of each individual jet due to the JES calibration is between $1$ and $4\,\%$ in the central region $(|\eta |<1.8)$, and increases to $5\,\%$ in the forward region $(1.8<|\eta |<4.5)$.

The uncertainties due to the JES calibration are propagated to the measured cross-sections using the MC simulation. The energy and $p_{\mathrm {T}}$ of each jet in the three-jet sample are scaled up or down by one standard deviation of a given uncertainty component, after which the luminosity-normalised three-jet event yield is measured from the resulting sample. The yields from the nominal sample and the samples where all jets were scaled up and down are unfolded, and the difference between each of these variations and the nominal result is taken as the uncertainty due to that JES uncertainty component. For example, the uncertainty in the three-jet cross-section in the $8<\left| Y^{*}\right| <10(\left| Y^{*}\right| <2)$ bin due to the LAr electromagnetic energy scale uncertainty increases from $2(3)$ to $10(8)\,\%$ with the $m_{jjj}$ increasing from $1(0.4)$  TeV to $4(3)$  TeV. In the same $\left| Y^{*}\right|$ bins, the uncertainty in the three-jet cross-section due to the uncertainty in the jet energy measurements in the forward region varies from $15(4)$ to $30(0.5)\,\%$, as a function of $m_{jjj}$. Since the sources of JES calibration uncertainty are uncorrelated with each other by construction, the corresponding uncertainty components in the cross-section are also taken as uncorrelated.

Each jet is affected by the additional energy deposited in the calorimeters due to pileup effects. Additional energy due to pileup is subtracted during the jet energy calibration procedure [7]. To check for any residual pileup effects in the measured cross-sections, the luminosity-normalised three-jet yields in all three-jet mass and rapidity-separation bins are split into bins of different pileup conditions under which the data were collected. No statistically significant deviation from the nominal result is observed.

The jet energy resolution (JER) is measured in the data using the bisector method in dijet events [45], where good agreement with the MC simulation is observed. The uncertainty in the JER is affected by selection parameters for jets, such as the amount of nearby jet activity, and depends on both jet $p_{\mathrm {T}}$ and jet $\eta$.

Jet angular resolution (JAR) is studied by matching particle-level jets to reconstruction-level jets in simulation. Jets are matched by requiring that the angular distance $\Delta R= \sqrt{\left( \Delta \phi \right) ^2+\left( \Delta y\right) ^2}$ between the particle-level and reconstruction-level jet is less than the jet radius parameter. The angular resolution is obtained from a Gaussian fit to the distribution of the difference of reconstruction-level and particle-level jet rapidity.

**Fig. 1:**
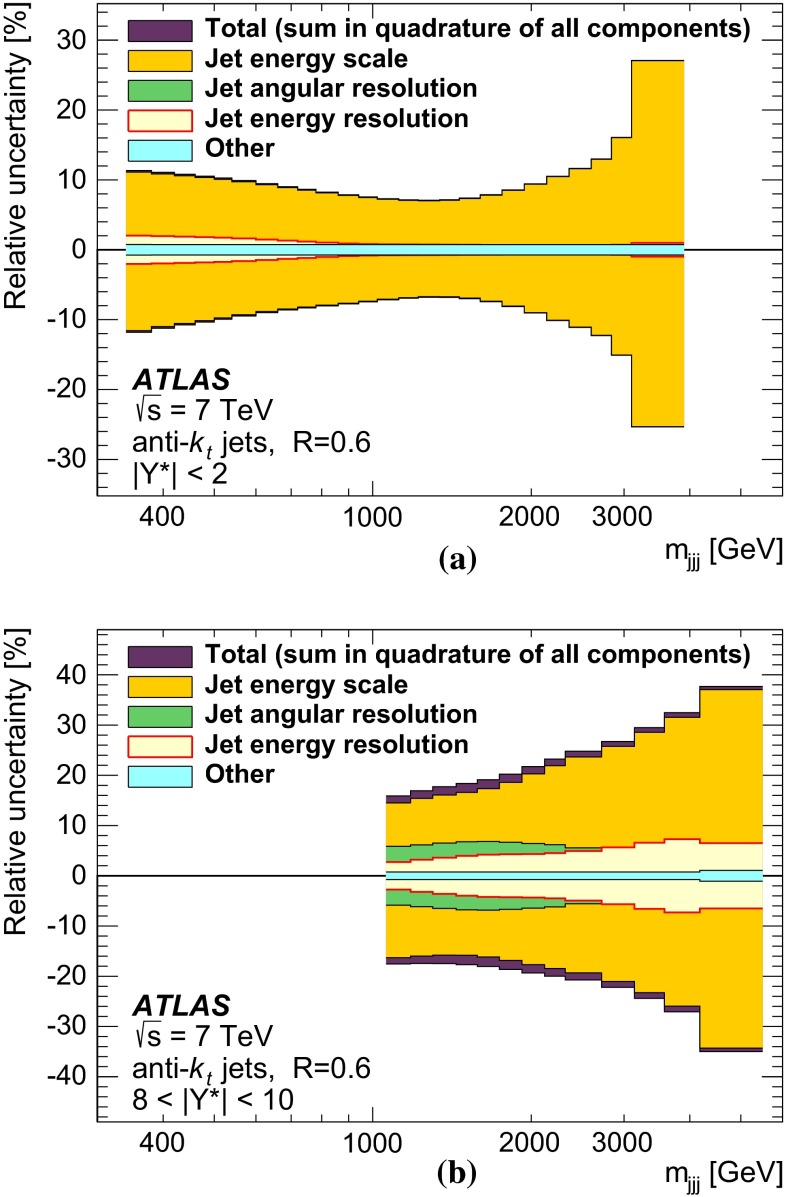
Total systematic uncertainty in the three-jet cross-section for anti-$k_{t}$ $R=0.6$ jets as a function of $m_{jjj}$ (a) in $\left| Y^{*}\right| <2$ and (b) $8<\left| Y^{*}\right| <10$ bins. The *bands* shows the uncertainties due to jet energy scale, jet angular resolution, jet energy resolution and the combined uncertainty due to jet quality selection and unfolding. The *outer band* represents the total experimental uncertainty

The difference between the JAR determined from the nominal MC simulation and that from the Alpgen sample is taken as a systematic uncertainty. The resolution varies between $0.005$ radians and $0.03$ radians depending on the jet $\eta$ and $p_{\mathrm {T}}$ values. The JAR uncertainty is about $10\text{-- }15\,\%$ for $p_{\mathrm {T}}{}<150$  GeV and decreases to $\sim 1\,\%$ for $p_{\mathrm {T}}>400$  GeV. The jet angular bias is found to be negligible.

The JER and JAR uncertainties are propagated to the measured cross-section through the unfolding transfer matrix. The energy and direction of each jet in the MC sample are smeared according to their uncertainties. To avoid being limited by statistical fluctuations this procedure is repeated 1000 times in each event. The average transfer matrix derived from these pseudo-experiments is used to unfold the three-jet yields, and the deviation from the three-jet yield unfolded using the nominal transfer matrix is taken as a symmetrised systematic uncertainty.

The uncertainty due to the jet reconstruction inefficiency as a function of jet $p_{\mathrm {T}}$ is estimated by comparing the efficiency for reconstructing a calorimeter jet, given the presence of an independently measured track-jet of the same radius, in data and in MC simulation [7, 46]. Since this method relies on tracking, its application is restricted to jets with $|\eta | < 1.9$ to ensure that both the $R=0.4$ and $R=0.6$ jets are fully within the tracker acceptance. For jets with $p_{\mathrm {T}}> 50$  GeV, relevant for this analysis, the reconstruction efficiency in both the data and the MC simulation is found to be $100\,\%$ for this rapidity region, leading to no additional uncertainty. The same efficiency is assumed for the forward region, where jets of a given $p_{\mathrm {T}}$ are more energetic and, therefore, their reconstruction efficiency is expected to be at least as good as that of jets in the central region.

The efficiencies for single-jet selection using the “medium” criteria agree within $0.25\,\%$ in data and MC simulation [42]. Because three jets are considered for each event selected for the analysis, a $0.75\,\%$ systematic uncertainty in the cross-section is assigned.

The impact of a possible mis-modelling of the shape of $m_{jjj}$ spectra in MC simulation, introduced through the unfolding as described in Sect. 6, is also included. The luminosity uncertainty is $1.8\,\%$ [38] and is fully correlated between all data points.

The total experimental uncertainty in the three-jet cross-section is summarised in Fig. 1. The total uncertainty ranges from $8\text{-- }10\,\%$ at low three-jet mass to $28\,\%$ at high three-jet mass for the range $\left| Y^{*}\right| < 6$ (see Appendix), and increases slightly for larger $\left| Y^{*}\right|$ bins. In the $8<\left| Y^{*}\right| <10$ bin the total uncertainty ranges from $18$ to $38\,\%$, where it is dominated by the jet energy scale uncertainty component for forward jets.

## Theoretical predictions and uncertainties

The NLO QCD predictions by the parton-level MC cross-section calculator NLOJET++ [47], corrected for hadronisation effects and underlying-event activity using Monte Carlo simulation with Perugia 2011 tune [19] of Pythia 6, are compared to the measured three-jet cross-sections.

### Fixed-order predictions

The fixed-order QCD calculations are performed with the NLOJET++ program interfaced to APPLgrid [48] for fast convolution with various PDF sets. The renormalisation ($Q_\mathrm {R}$) and factorisation ($Q_\mathrm {F}$) scales are set to the mass of the three-jet system, $Q = Q_\mathrm {R} = Q_\mathrm {F} = m_{jjj}.$ The following proton PDF sets are considered for the theoretical predictions: CT 10 [49], GJR 08 [50], MSTW 2008 [51], NNPDF 2.3 [52], HERAPDF 1.5 [53], and ABM 11 [54].

To estimate the uncertainty due to missing higher-order terms in the fixed-order perturbative expansion, the renormalisation scale is varied up and down by a factor of two. The uncertainty due to the dependence of the theoretical predictions on the factorisation scale, which specifies the separation between the short-distance hard scattering and long-distance non-perturbative dynamics, is estimated by varying the factorisation scale up and down by a factor of two. All permutations of these two scale choices are considered, except the cases where the scales are shifted in opposite directions. The maximum deviations from the nominal prediction are taken as the scale uncertainty. The scale uncertainty is generally $10{-}20\,\%$ depending on the $m_{jjj}$.

The multiple uncorrelated uncertainty components of each PDF set, as provided by the various PDF analyses, are also propagated through the theoretical calculations. The PDF groups generally derive these from the experimental uncertainties in the data used in the fits. For the results shown in Sect. 9, the standard Hessian sum in quadrature [55] of the various independent components is calculated taking into account asymmetries of the uncertainty components. The NNPDF 2.3 PDF set is an exception, where uncertainties are expressed in terms of *replicas* instead of independent components. These replicas represent a collection of equally likely PDF sets, where the data used in the PDF fit were fluctuated within their experimental uncertainties. For the plots shown in Sect. 9, the uncertainties in the NNPDF 2.3 PDF set are evaluated as the RMS of the replicas in each bin of $m_{jjj}$, producing equivalent PDF uncertainties in the theoretical predictions. These uncertainties are symmetric by construction. Where needed, the uncertainties of PDF sets are rescaled to the $68\,\%$ confidence level (CL). HERAPDF provides three types of uncertainties: experimental, model and parameterisation. The three uncertainty sources are added in quadrature to get a total PDF uncertainty.

The uncertainties in the cross-sections due to the strong coupling, $\alpha _{\mathrm {s}}$, are estimated using two additional proton PDF sets, for which different values of $\alpha _{\mathrm {s}}$ are assumed in the fits, such that the effect of the strong coupling value on the PDFs is included. This follows Ref. [56]. The resulting uncertainty is approximately $3\,\%$ across all three-jet mass and $\left| Y^{*}\right|$ ranges considered.

The scale uncertainties are dominant in low and intermediate three-jet mass regions, while the PDF uncertainties become dominant at high $m_{jjj}$. The uncertainties in the theoretical predictions due to those on the PDFs range from $5\,\%$ at low $m_{jjj}$ to $30\,\%$ at high three-jet mass for the range of $\left| Y^{*}\right|$ values up to four. For the values of $\left| Y^{*}\right|$ between four and ten, the PDF uncertainties reach $40\text{-- }80\,\%$ at high three-jet mass, depending on the PDF set and the $\left| Y^{*}\right|$ value.

### Non-perturbative effects

**Fig. 2:**
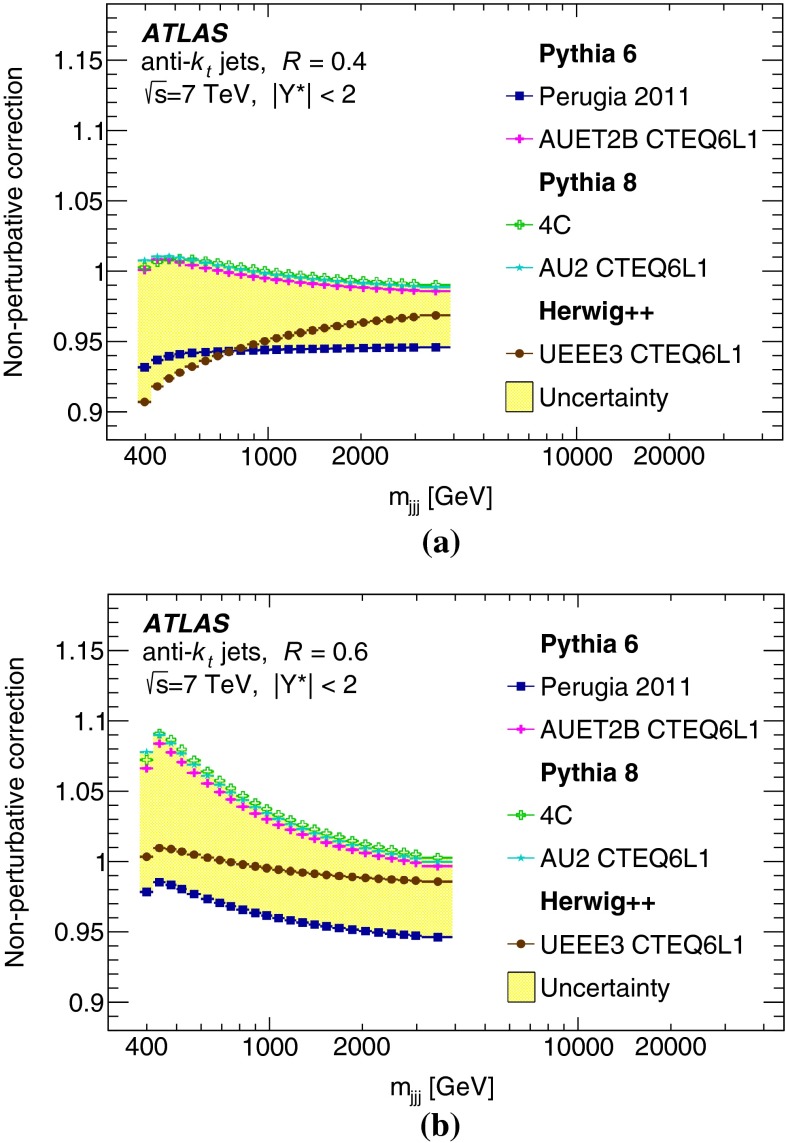
Non-perturbative corrections obtained using various MC generators and tunes for the differential three-jet cross-section as a function of three-jet mass in the range $\left| Y^{*}\right| <2$ for anti-$k_{t}$ jet **a** $R=0.4$ and **b** $R=0.6$

Non-perturbative corrections (NPC) are evaluated using leading-logarithmic parton-shower generators, separately for each value of the jet radius parameter. The corrections are calculated as bin-by-bin ratios of the three-jet differential cross-section at the particle level, including hadronisation and underlying-event effects, to that at parton-level after the parton shower (before the hadronisation process starts) with the underlying-event simulation switched off. The nominal corrections are calculated using Pythia 6 with the Perugia 2011 tune. The non-perturbative corrections as a function of three-jet mass are shown in Fig. 2 for the range $\left| Y^{*}\right| <2$ for $R=0.4$ and $R=0.6$ jets. The NPC are smaller than $10\,\%$ in all $m_{jjj}$ and $\left| Y^{*}\right|$ bins.

The uncertainties in the non-perturbative corrections, arising from the modelling of the hadronisation process and the underlying event, are estimated as the maximum deviations of the corrections from the nominal ones, using the following configurations: Pythia 8 with the 4C [26] and AU2 [21] tunes using the CTEQ6L1 PDF set [32]; Pythia 6 with the AUET2B [22] tune with CTEQ6L1; and Herwig++ 2.6.3 [57, 58] with the UE-EE-3 tune [59] using the CTEQ6L1 set. The uncertainty in the non-perturbative corrections ranges up to $\sim 10\,\%$ depending on the three-jet mass in all $\left| Y^{*}\right|$ bins.

The total theoretical uncertainty is calculated as a sum in quadrature of PDF, scale, $\alpha _{\mathrm {s}}$ and NPC uncertainties.

## Cross-section results

**Fig. 3:**
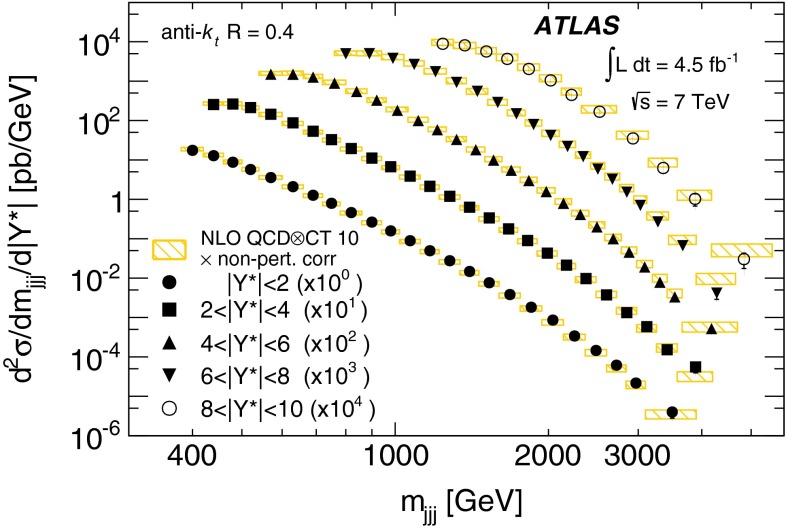
The three-jet double-differential cross-section as a function of $m_{jjj}$ in bins $\left| Y^{*}\right|$, as denoted in the legend. The jets are identified using the anti-$k_{t}$ algorithm with $R=0.4$. For convenience, the cross-sections are multiplied by the factors indicated in the legend. Also shown is the comparison with the NLOJET++ prediction with the CT 10 PDF set corrected for non-perturbative effects. The statistical uncertainties are smaller than the size of the symbols. Where visible, the sum in quadrature of the statistical and experimental systematic uncertainties is plotted

**Fig. 4:**
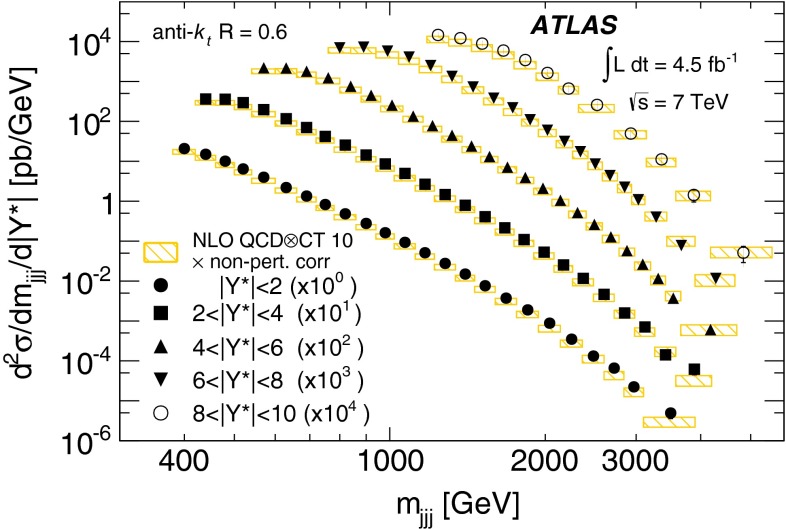
The three-jet double-differential cross-section as a function of $m_{jjj}$ in bins $\left| Y^{*}\right|$, as denoted in the legend. The jets are identified using the anti-$k_{t}$ algorithm with $R=0.6$. For convenience, the cross-sections are multiplied by the factors indicated in the legend. Also shown is the comparison with the NLOJET++ prediction with the CT 10 PDF set corrected for non-perturbative effects. The statistical uncertainties are smaller than the size of the symbols. Where visible, the sum in quadrature of the statistical and experimental systematic uncertainties is plotted

Measurements of the double-differential three-jet cross-sections as a function of the three-jet mass in various ranges of $\left| Y^{*}\right|$ are shown in Figs. 3 and 4 for anti-$k_{t}$ jets with values of the radius parameter $R=0.4$ and $R=0.6$, respectively. The cross-section decreases rapidly as a function of the three-jet mass. The NLO QCD calculations using NLOJET++ with the CT 10 PDF set corrected for non-perturbative effects are compared to the measured cross-sections. Good agreement between the data and the theoretical predictions is found over the full kinematic range, covering almost seven orders of magnitude in the measured cross-section values.

**Fig. 5:**
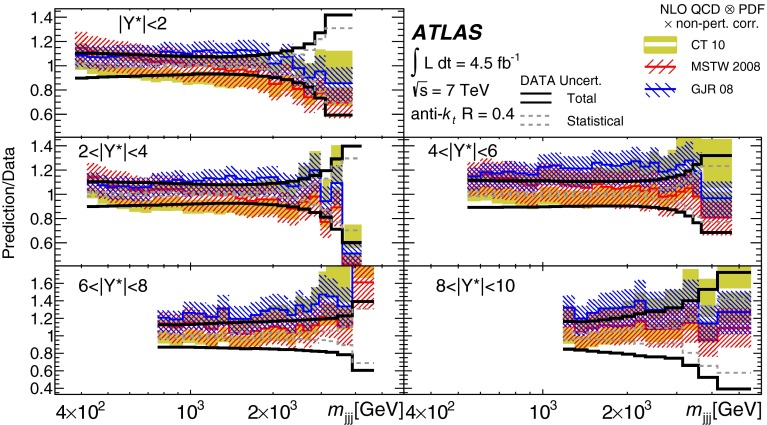
The ratio of NLO QCD predictions, obtained by using NLOJET++ with different PDF sets (CT 10, MSTW 2008, GJR 08) and corrected for non-perturbative effects, to data as a function of $m_{jjj}$ in bins of $\left| Y^{*}\right|$, as denoted in the legend. The ratios are for jets identified using the anti-$k_{t}$ algorithm with $R=0.4$. The experimental error bands are centered at one and designate the relative statistical (*thin dashed line*) and total (statistical and systematic uncertainties added in quadrature) experimental uncertainties (*thick solid line*). The theoretical predictions are represented by *thick lines* with the *hatched* or *filled band* around it. The *line* show the central values and the *band* represent the total theory uncertainty

**Fig. 6:**
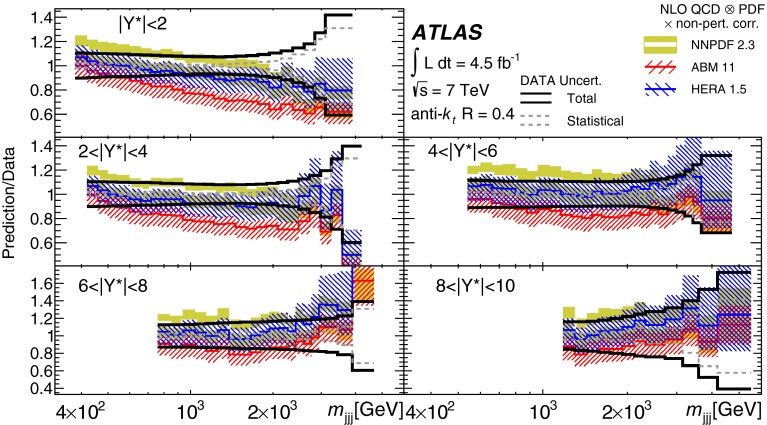
The ratio of NLO QCD predictions, obtained by using NLOJET++ with different PDF sets (NNPDF 2.3, ABM 11, HERAPDF 1.5) and corrected for non-perturbative effects, to data as a function of $m_{jjj}$ in bins of $\left| Y^{*}\right|$, as denoted in the legend. The ratios are for jets identified using the anti-$k_{t}$ algorithm with $R=0.4$. The experimental error bands are centered at one and designate the relative statistical (*thin dashed line*) and total (statistical and systematic uncertainties added in quadrature) experimental uncertainties (*thick solid line*). The theoretical predictions are represented by *thick lines* with the *hatched* or *filled band* around it. The *line* show the central values and the *band* represent the total theory uncertainty

**Fig. 7:**
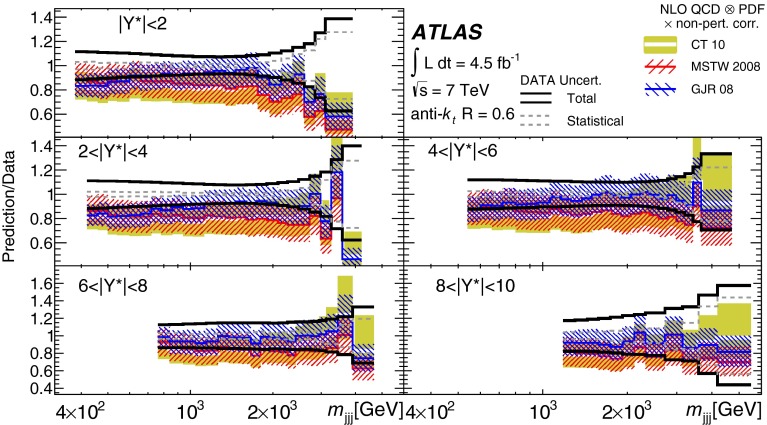
The ratio of NLO QCD predictions, obtained by using NLOJET++ with different PDF sets (CT 10, MSTW 2008, GJR 08) and corrected for non-perturbative effects, to data as a function of $m_{jjj}$ in bins of $\left| Y^{*}\right|$, as denoted in the legend. The ratios are for jets identified using the anti-$k_{t}$ algorithm with $R=0.6$. The experimental error bands are centered at one and designate the relative statistical (*thin dashed line*) and total (statistical and systematic uncertainties added in quadrature) experimental uncertainties (*thick solid line*). The theoretical predictions are represented by *thick lines* with the *hatched* or *filled band* around it. The *line* show the central values and the *band* represent the total theory uncertainty

**Fig. 8:**
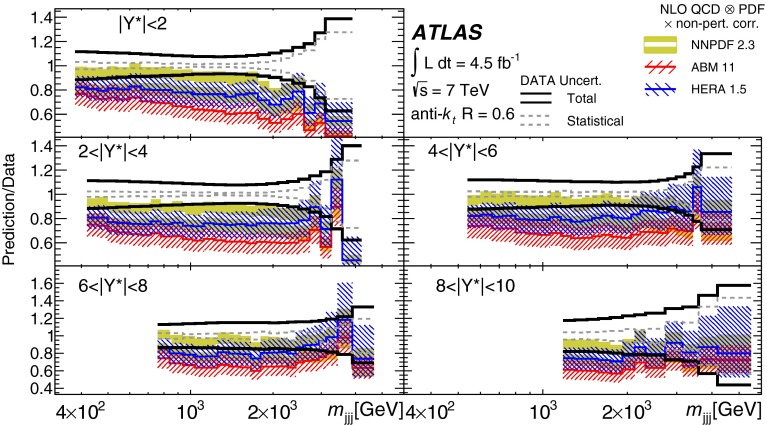
The ratio of NLO QCD predictions, obtained by using NLOJET++ with different PDF sets (NNPDF 2.3, ABM 11, HERAPDF 1.5) and corrected for non-perturbative effects, to data as a function of $m_{jjj}$ in bins of $\left| Y^{*}\right|$, as denoted in the legend. The ratios are for jets identified using the anti-$k_{t}$ algorithm with $R=0.6$. The experimental error bands are centered at one and designate the relative statistical (*thin dashed line*) and total (statistical and systematic uncertainties added in quadrature) experimental uncertainties (*thick solid line*). The theoretical predictions are represented by *thick lines* with the *hatched* or *filled band* around it. The *line* show the central values and the *band* represent the total theory uncertainty

The ratios of the theoretical predictions calculated with various PDF sets to the measured cross-sections are presented in Figs. 5 and 6 for $R=0.4$ jets and in Figs. 7 and 8 for $R=0.6$ jets. Theoretical calculations that use CT 10, MSTW 2008 and GJR 08 PDFs are compared to data in Figs. 5 and 7 and comparisons to other global PDFs, namely NNPDF 2.3, ABM 11 and HERAPDF 1.5. are presented in Figs. 6 and 8.

The three-jet cross-sections are well described by the calculations that use CT 10, NNPDF 2.3, GJR 08, MSTW 2008 and HERAPDF 1.5 PDFs. Disagreement between data and the predictions using ABM 11 PDFs is observed for most of the cross-sections measured with both jet radius parameters.

For all PDF sets, the predictions for anti-$$k_{t}$$$$R=0.4$$ jets agree well with measured cross-sections, while the calculations that use the ABM 11 PDF set are systematically below all other theory curves. Theory predictions for anti-$$k_{t}$$$$R=0.6$$ jets underestimate the data across the full $$m_{jjj}$$–$$\left| Y^{*}\right| $$ plane. This shift is within the experimental and theoretical uncertainties. The jet radius dependence of theory-to-data ratios is similar for all PDF sets considered, demonstrating that this tendency is independent of the assumptions made in different PDF determinations.

## Conclusions

Cross-section measurements of three-jet production in $pp$ collisions at 7  TeV centre-of-mass energy as a function of the three-jet mass, in bins of the sum of the absolute rapidity separations between the three leading jets are presented. Jets are reconstructed with the anti-$k_{t}$ algorithm using two values of the radius parameter, $R=0.4$ and $R=0.6$. The measurements are based on the full data set collected with the ATLAS detector during 2011 data-taking at the LHC, corresponding to an integrated luminosity of $4.51~\text{ fb }^{-1}$. The measurements are corrected for detector effects and reported at the particle level. The total experimental uncertainty in these measurements is dominated by the jet energy scale calibration uncertainty. The measurement uncertainties are smaller than, or similar to, those in the theoretical predictions.

The measurements probe three-jet masses up to

$\sim 5$  TeV and are well described by perturbative QCD at NLO accuracy across the full $m_{jjj}$–$\left| Y^{*}\right|$ plane. The comparison of NLO QCD predictions corrected for non-perturbative effects to the measured cross-sections is performed using several modern PDF sets. The data are well described by the theoretical predictions when using CT 10, NNPDF 2.3, HERAPDF 1.5, GJR 08 and MSTW 2008 PDFs. The theoretical calculations based on the ABM 11 PDFs are systematically below all the other predictions.

Comparison of measured cross-sections to theoretical predictions for two different jet radius parameters shows good agreement for $R=0.4$ jets but shifted theory-to-data ratios for $R=0.6$ jets. This shift is covered by the experimental and theoretical uncertainty bands and it has only a minor dependence on the PDF set used.

## Appendix

Tables 1, 2, 3, 4, 5,6, 7, 8, 9 and 10 of measured cross-sections

**Table 1:** Measured double-differential three-jet cross-section, $\sigma$, for $R=0.4{}$ jets and $\left| Y^{*}\right| {}<2$, along with uncertainties in the measurement. All uncertainties are given in %, where $\delta _\text {stat}^\text {data}$ ($\delta _\text {stat}^\text {MC}$) are the statistical uncertainties in the data (MC simulation). The $\gamma$ components are the uncertainty in the jet energy calibration from the in situ, the pileup, the close-by jet, and flavour components. The $u$ components show the uncertainty for the jet energy and angular resolution, the unfolding, the quality selection, and the luminosity. While all columns are uncorrelated with each other, the in situ, pileup, and flavour uncertainties shown here are the sum in quadrature of multiple uncorrelated components

$m_{jjj}$ (bin #)	$m_{jjj}$-range (TeV)	$\sigma$ (pb/GeV)	$\delta _{\text {stat}}^{\text {data}}$ (%)	$\delta _{\text {stat}}^{\text {MC}}$ (%)	$\gamma _{\text {in-situ}}$ (%)	$\gamma _{\text {pileup}}$ (%)	$\gamma _{\text {close-by}}$ (%)	$\gamma _{\text {flavour}}$ (%)	$u_{\text {JER}}$ (%)	$u_{\text {JAR}}$ (%)	$u_{\text {unfold}}$ (%)	$u_{\text {qual.}}$ (%)	$u_{\text {lumi}}$ (%)
$$1$$	$$ 0.38{-}0.42$$	17.5	1.8	0.73	$$^{+6.4}_{-6.3}$$	$$^{+0.4}_{-2.7}$$	$$^{+3.3}_{-3.4}$$	$$^{+7.0}_{-6.6}$$	0.7	0.0	0.0	0.75	1.8
$$2$$	$$ 0.42{-}0.46$$	12.8	2.0	0.62	$$^{+6.2}_{-6.1}$$	$$^{+0.3}_{-1.9}$$	$$^{+3.2}_{-3.2}$$	$$^{+6.7}_{-6.3}$$	0.8	0.0	0.0	0.75	1.8
$$3$$	$$ 0.46{-}0.50$$	8.75	1.3	0.50	$$^{+6.1}_{-6.0}$$	$$^{+0.2}_{-1.4}$$	$$^{+3.2}_{-3.2}$$	$$^{+6.5}_{-6.2}$$	0.8	0.0	0.0	0.75	1.8
$$4$$	$$ 0.50{-}0.54$$	5.72	1.5	0.55	$$^{+6.0}_{-5.9}$$	$$^{+0.1}_{-1.2}$$	$$^{+3.2}_{-3.2}$$	$$^{+6.3}_{-6.0}$$	0.8	0.0	0.0	0.75	1.8
$$5$$	$$ 0.54{-}0.60$$	3.57	1.8	0.49	$$^{+5.9}_{-5.7}$$	$$^{+0.1}_{-1.1}$$	$$^{+3.2}_{-3.2}$$	$$^{+6.0}_{-5.7}$$	0.8	0.0	0.0	0.75	1.8
$$6$$	$$ 0.60{-}0.66$$	2.09	1.6	0.49	$$^{+5.7}_{-5.6}$$	$$^{+0.3}_{-1.1}$$	$$^{+3.3}_{-3.2}$$	$$^{+5.7}_{-5.4}$$	0.7	0.0	0.0	0.75	1.8
$$7$$	$$ 0.66{-}0.72$$	1.27	1.0	0.55	$$^{+5.5}_{-5.4}$$	$$^{+0.4}_{-1.1}$$	$$^{+3.3}_{-3.3}$$	$$^{+5.4}_{-5.2}$$	0.7	0.0	0.0	0.75	1.8
$$8$$	$$ 0.72{-}0.78$$	$$ 7.93\times 10^{-1}$$	1.1	0.53	$$^{+5.4}_{-5.3}$$	$$^{+0.4}_{-1.1}$$	$$^{+3.3}_{-3.2}$$	$$^{+5.1}_{-4.9}$$	0.6	0.0	0.0	0.75	1.8
$$9$$	$$ 0.78{-}0.86$$	$$ 4.61\times 10^{-1}$$	0.91	0.42	$$^{+5.3}_{-5.2}$$	$$^{+0.5}_{-1.0}$$	$$^{+3.2}_{-3.1}$$	$$^{+4.9}_{-4.7}$$	0.6	0.0	0.0	0.75	1.8
$$10$$	$$ 0.86{-}0.94$$	$$ 2.64\times 10^{-1}$$	0.69	0.33	$$^{+5.2}_{-5.1}$$	$$^{+0.4}_{-0.9}$$	$$^{+3.0}_{-2.9}$$	$$^{+4.7}_{-4.5}$$	0.6	0.0	0.0	0.75	1.8
$$11$$	$$ 0.94{-}1.02$$	$$ 1.58\times 10^{-1}$$	0.82	0.32	$$^{+5.2}_{-5.1}$$	$$^{+0.3}_{-0.8}$$	$$^{+2.8}_{-2.7}$$	$$^{+4.4}_{-4.3}$$	0.6	0.0	0.0	0.75	1.8
$$12$$	$$ 1.02{-}1.12$$	$$ 8.91\times 10^{-2}$$	0.58	0.34	$$^{+5.2}_{-5.1}$$	$$^{+0.2}_{-0.6}$$	$$^{+2.4}_{-2.4}$$	$$^{+4.2}_{-4.1}$$	0.6	0.0	0.0	0.75	1.8
$$13$$	$$ 1.12{-}1.22$$	$$ 4.96\times 10^{-2}$$	0.71	0.42	$$^{+5.4}_{-5.2}$$	$$^{+0.2}_{-0.5}$$	$$^{+2.1}_{-2.0}$$	$$^{+4.0}_{-3.9}$$	0.6	0.0	0.0	0.75	1.8
$$14$$	$$ 1.22{-}1.34$$	$$ 2.76\times 10^{-2}$$	0.92	0.39	$$^{+5.6}_{-5.4}$$	$$^{+0.2}_{-0.4}$$	$$^{+1.8}_{-1.8}$$	$$^{+3.9}_{-3.7}$$	0.6	0.0	0.0	0.75	1.8
$$15$$	$$ 1.34{-}1.46$$	$$ 1.48\times 10^{-2}$$	1.2	0.46	$$^{+5.9}_{-5.7}$$	$$^{+0.2}_{-0.4}$$	$$^{+1.6}_{-1.5}$$	$$^{+3.7}_{-3.6}$$	0.6	0.0	0.0	0.75	1.8
$$16$$	$$ 1.46{-}1.60$$	$$ 7.63\times 10^{-3}$$	1.6	0.39	$$^{+6.3}_{-6.1}$$	$$^{+0.2}_{-0.4}$$	$$^{+1.3}_{-1.3}$$	$$^{+3.6}_{-3.5}$$	0.5	0.0	0.0	0.75	1.8
$$17$$	$$ 1.60{-}1.76$$	$$ 3.83\times 10^{-3}$$	2.1	0.38	$$^{+6.9}_{-6.7}$$	$$^{+0.1}_{-0.4}$$	$$^{+1.2}_{-1.2}$$	$$^{+3.5}_{-3.4}$$	0.6	0.0	0.0	0.75	1.8
$$18$$	$$ 1.76{-}1.94$$	$$ 1.82\times 10^{-3}$$	2.9	0.38	$$^{+7.7}_{-7.5}$$	$$^{+0.1}_{-0.3}$$	$$^{+1.0}_{-1.0}$$	$$^{+3.4}_{-3.3}$$	0.6	0.0	0.0	0.75	1.8
$$19$$	$$ 1.94{-}2.14$$	$$ 8.60\times 10^{-4}$$	4.0	0.37	$$^{+8.7}_{-8.4}$$	$$^{+0.0}_{-0.2}$$	$$^{+0.9}_{-0.9}$$	$$^{+3.4}_{-3.2}$$	0.6	0.0	0.0	0.75	1.8
$$20$$	$$ 2.14{-}2.36$$	$$ 3.40\times 10^{-4}$$	6.0	0.54	$$^{+9.8}_{-9.4}$$	$$^{+0.0}_{-0.1}$$	$$^{+0.9}_{-0.8}$$	$$^{+3.3}_{-3.1}$$	0.6	0.0	0.0	0.75	1.8
$$21$$	$$ 2.36{-}2.60$$	$$ 1.46\times 10^{-4}$$	9.1	0.70	$$^{+10.8}_{-10.4}$$	$$^{+0.0}_{-0.1}$$	$$^{+0.8}_{-0.8}$$	$$^{+3.2}_{-3.1}$$	0.7	0.0	0.0	0.75	1.8
$$22$$	$$ 2.60{-}2.84$$	$$ 6.16\times 10^{-5}$$	13	0.79	$$^{+11.9}_{-11.8}$$	$$^{+0.0}_{-0.1}$$	$$^{+0.8}_{-0.8}$$	$$^{+3.1}_{-3.1}$$	0.7	0.0	0.0	0.75	1.8
$$23$$	$$ 2.84{-}3.10$$	$$ 2.17\times 10^{-5}$$	22	1.1	$$^{+15.4}_{-15.5}$$	$$^{+0.0}_{-0.1}$$	$$^{+0.8}_{-0.7}$$	$$^{+3.0}_{-3.1}$$	0.8	0.0	0.0	0.75	1.8
$$24$$	$$ 3.10{-}3.90$$	$$ 4.00\times 10^{-6}$$	31	0.87	$$^{+27.9}_{-26.9}$$	$$^{+0.0}_{-0.1}$$	$$^{+0.7}_{-0.7}$$	$$^{+3.0}_{-3.1}$$	1.3	0.0	0.3	0.75	1.8

**Table 2:** Measured double-differential three-jet cross-section, $\sigma$, for $R=0.6{}$ jets and $\left| Y^{*}\right| {}<2$, along with uncertainties in the measurement. All uncertainties are given in %, where $\delta _\text {stat}^\text {data}$ ($\delta _\text {stat}^\text {MC}$) are the statistical uncertainties in the data (MC simulation). The $\gamma$ components are the uncertainty in the jet energy calibration from the in situ, the pileup, the close-by jet, and flavour components. The $u$ components show the uncertainty for the jet energy and angular resolution, the unfolding, the quality selection, and the luminosity. While all columns are uncorrelated with each other, the in situ, pileup, and flavour uncertainties shown here are the sum in quadrature of multiple uncorrelated components

$m_{jjj}$ (bin #)	$m_{jjj}$-range (TeV)	$\sigma$ (pb/GeV)	$\delta _{\text {stat}}^{\text {data}}$ (%)	$\delta _{\text {stat}}^{\text {MC}}$ (%)	$\gamma _{\text {in-situ}}$ (%)	$\gamma _{\text {pileup}}$ (%)	$\gamma _{\text {close-by}}$ (%)	$\gamma _{\text {flavour}}$ (%)	$u_{\text {JER}}$ (%)	$u_{\text {JAR}}$ (%)	$u_{\text {unfold}}$ (%)	$u_{\text {qual.}}$ (%)	$u_{\text {lumi}}$ (%)
$$1$$	$$ 0.38{-}0.42$$	20.8	2.9	0.91	$$^{+6.6}_{-6.6}$$	$$^{+0.1}_{-3.4}$$	$$^{+5.0}_{-4.6}$$	$$^{+7.0}_{-6.6}$$	2.0	0.7	0.0	0.75	1.8
$$2$$	$$ 0.42{-}0.46$$	15.0	3.1	0.81	$$^{+6.5}_{-6.5}$$	$$^{+0.1}_{-2.6}$$	$$^{+4.8}_{-4.4}$$	$$^{+6.8}_{-6.5}$$	1.9	0.6	0.0	0.75	1.8
$$3$$	$$ 0.46{-}0.50$$	10.1	2.1	0.60	$$^{+6.4}_{-6.4}$$	$$^{+0.1}_{-2.2}$$	$$^{+4.6}_{-4.3}$$	$$^{+6.7}_{-6.3}$$	1.8	0.5	0.0	0.75	1.8
$$4$$	$$ 0.50{-}0.54$$	6.44	2.4	0.59	$$^{+6.3}_{-6.2}$$	$$^{+0.1}_{-1.9}$$	$$^{+4.4}_{-4.1}$$	$$^{+6.5}_{-6.0}$$	1.7	0.4	0.0	0.75	1.8
$$5$$	$$ 0.54{-}0.60$$	3.99	2.2	0.49	$$^{+6.2}_{-5.9}$$	$$^{+0.2}_{-1.6}$$	$$^{+4.3}_{-3.9}$$	$$^{+6.2}_{-5.8}$$	1.6	0.3	0.0	0.75	1.8
$$6$$	$$ 0.60{-}0.66$$	2.20	2.1	0.53	$$^{+6.0}_{-5.7}$$	$$^{+0.4}_{-1.3}$$	$$^{+4.1}_{-3.8}$$	$$^{+5.9}_{-5.4}$$	1.5	0.2	0.0	0.75	1.8
$$7$$	$$ 0.66{-}0.72$$	1.35	2.6	0.63	$$^{+5.8}_{-5.5}$$	$$^{+0.5}_{-1.1}$$	$$^{+3.9}_{-3.7}$$	$$^{+5.5}_{-5.2}$$	1.3	0.1	0.0	0.75	1.8
$$8$$	$$ 0.72{-}0.78$$	$$ 8.27\times 10^{-1}$$	2.4	0.67	$$^{+5.6}_{-5.4}$$	$$^{+0.6}_{-1.1}$$	$$^{+3.7}_{-3.6}$$	$$^{+5.2}_{-4.9}$$	1.2	0.1	0.0	0.75	1.8
$$9$$	$$ 0.78{-}0.86$$	$$ 4.83\times 10^{-1}$$	1.4	0.57	$$^{+5.4}_{-5.3}$$	$$^{+0.6}_{-1.1}$$	$$^{+3.5}_{-3.4}$$	$$^{+4.9}_{-4.7}$$	1.0	0.0	0.0	0.75	1.8
$$10$$	$$ 0.86{-}0.94$$	$$ 2.78\times 10^{-1}$$	1.8	0.45	$$^{+5.3}_{-5.3}$$	$$^{+0.6}_{-1.1}$$	$$^{+3.2}_{-3.1}$$	$$^{+4.6}_{-4.4}$$	0.9	0.0	0.0	0.75	1.8
$$11$$	$$ 0.94{-}1.02$$	$$ 1.62\times 10^{-1}$$	1.5	0.43	$$^{+5.3}_{-5.2}$$	$$^{+0.6}_{-1.1}$$	$$^{+2.9}_{-2.9}$$	$$^{+4.4}_{-4.2}$$	0.8	0.0	0.0	0.75	1.8
$$12$$	$$ 1.02{-}1.12$$	$$ 9.31\times 10^{-2}$$	1.0	0.38	$$^{+5.3}_{-5.2}$$	$$^{+0.5}_{-1.1}$$	$$^{+2.6}_{-2.5}$$	$$^{+4.1}_{-3.9}$$	0.8	0.0	0.0	0.75	1.8
$$13$$	$$ 1.12{-}1.22$$	$$ 5.12\times 10^{-2}$$	1.4	0.44	$$^{+5.4}_{-5.2}$$	$$^{+0.3}_{-0.9}$$	$$^{+2.3}_{-2.2}$$	$$^{+3.9}_{-3.7}$$	0.8	0.0	0.0	0.75	1.8
$$14$$	$$ 1.22{-}1.34$$	$$ 2.77\times 10^{-2}$$	1.3	0.47	$$^{+5.7}_{-5.4}$$	$$^{+0.2}_{-0.7}$$	$$^{+2.0}_{-1.9}$$	$$^{+3.6}_{-3.5}$$	0.8	0.0	0.0	0.75	1.8
$$15$$	$$ 1.34{-}1.46$$	$$ 1.50\times 10^{-2}$$	1.2	0.49	$$^{+5.9}_{-5.6}$$	$$^{+0.1}_{-0.5}$$	$$^{+1.7}_{-1.7}$$	$$^{+3.4}_{-3.2}$$	0.7	0.0	0.0	0.75	1.8
$$16$$	$$ 1.46{-}1.60$$	$$ 7.63\times 10^{-3}$$	1.6	0.50	$$^{+6.4}_{-6.0}$$	$$^{+0.2}_{-0.3}$$	$$^{+1.6}_{-1.5}$$	$$^{+3.3}_{-3.1}$$	0.7	0.0	0.0	0.75	1.8
$$17$$	$$ 1.60{-}1.76$$	$$ 3.73\times 10^{-3}$$	2.0	0.44	$$^{+7.0}_{-6.6}$$	$$^{+0.2}_{-0.3}$$	$$^{+1.4}_{-1.4}$$	$$^{+3.1}_{-2.9}$$	0.7	0.0	0.0	0.75	1.8
$$18$$	$$ 1.76{-}1.94$$	$$ 1.90\times 10^{-3}$$	2.8	0.38	$$^{+7.8}_{-7.4}$$	$$^{+0.3}_{-0.2}$$	$$^{+1.3}_{-1.2}$$	$$^{+3.0}_{-2.8}$$	0.7	0.0	0.0	0.75	1.8
$$19$$	$$ 1.94{-}2.14$$	$$ 8.81\times 10^{-4}$$	3.9	0.40	$$^{+8.8}_{-8.5}$$	$$^{+0.4}_{-0.2}$$	$$^{+1.2}_{-1.1}$$	$$^{+2.9}_{-2.8}$$	0.6	0.0	0.0	0.75	1.8
$$20$$	$$ 2.14{-}2.36$$	$$ 3.50\times 10^{-4}$$	5.9	0.58	$$^{+10.0}_{-9.7}$$	$$^{+0.4}_{-0.2}$$	$$^{+1.1}_{-1.1}$$	$$^{+2.9}_{-2.7}$$	0.6	0.0	0.0	0.75	1.8
$$21$$	$$ 2.36{-}2.60$$	$$ 1.32\times 10^{-4}$$	9.4	0.70	$$^{+11.2}_{-10.7}$$	$$^{+0.4}_{-0.2}$$	$$^{+1.0}_{-1.0}$$	$$^{+2.8}_{-2.6}$$	0.6	0.0	0.0	0.75	1.8
$$22$$	$$ 2.60{-}2.84$$	$$ 6.60\times 10^{-5}$$	13	0.83	$$^{+12.6}_{-11.9}$$	$$^{+0.4}_{-0.2}$$	$$^{+1.0}_{-1.0}$$	$$^{+2.8}_{-2.6}$$	0.7	0.0	0.0	0.75	1.8
$$23$$	$$ 2.84{-}3.10$$	$$ 2.24\times 10^{-5}$$	22	1.2	$$^{+15.8}_{-14.8}$$	$$^{+0.4}_{-0.2}$$	$$^{+1.0}_{-1.0}$$	$$^{+2.8}_{-2.5}$$	0.7	0.0	0.0	0.75	1.8
$$24$$	$$ 3.10{-}3.90$$	$$ 4.95\times 10^{-6}$$	27	0.93	$$^{+26.9}_{-25.2}$$	$$^{+0.4}_{-0.2}$$	$$^{+1.0}_{-0.9}$$	$$^{+2.6}_{-2.4}$$	1.0	0.0	0.0	0.75	1.8

**Table 3:** Measured double-differential three-jet cross-section, $\sigma$, for $R=0.4{}$ jets and $2\le \left| Y^{*}\right| {}<4$, along with uncertainties in the measurement. All uncertainties are given in %, where $\delta _\text {stat}^\text {data}$ ($\delta _\text {stat}^\text {MC}$) are the statistical uncertainties in the data (MC simulation). The $\gamma$ components are the uncertainty in the jet energy calibration from the in situ, the pileup, the close-by jet, and flavour components. The $u$ components show the uncertainty for the jet energy and angular resolution, the unfolding, the quality selection, and the luminosity. While all columns are uncorrelated with each other, the in situ, pileup, and flavour uncertainties shown here are the sum in quadrature of multiple uncorrelated components

$m_{jjj}$ (bin #)	$m_{jjj}$-range (TeV)	$\sigma$ (pb/GeV)	$\delta _{\text {stat}}^{\text {data}}$ (%)	$\delta _{\text {stat}}^{\text {MC}}$ (%)	$\gamma _{\text {in-situ}}$ (%)	$\gamma _{\text {pileup}}$ (%)	$\gamma _{\text {close-by}}$ (%)	$\gamma _{\text {flavour}}$ (%)	$u_{\text {JER}}$ (%)	$u_{\text {JAR}}$ (%)	$u_{\text {unfold}}$ (%)	$u_{\text {qual.}}$ (%)	$u_{\text {lumi}}$ (%)
$$1$$	$$ 0.42{-}0.46$$	25.5	1.4	0.83	$$^{+6.6}_{-6.5}$$	$$^{+0.2}_{-4.1}$$	$$^{+2.9}_{-2.8}$$	$$^{+7.0}_{-6.2}$$	0.6	0.2	0.0	0.75	1.8
$$2$$	$$ 0.46{-}0.50$$	26.3	1.4	0.66	$$^{+6.6}_{-6.5}$$	$$^{+0.2}_{-3.2}$$	$$^{+2.8}_{-2.7}$$	$$^{+6.9}_{-6.2}$$	0.7	0.2	0.0	0.75	1.8
$$3$$	$$ 0.50{-}0.54$$	21.3	0.90	0.60	$$^{+6.7}_{-6.4}$$	$$^{+0.2}_{-2.4}$$	$$^{+2.8}_{-2.7}$$	$$^{+6.7}_{-6.1}$$	0.7	0.2	0.0	0.75	1.8
$$4$$	$$ 0.54{-}0.60$$	14.4	0.85	0.43	$$^{+6.7}_{-6.4}$$	$$^{+0.2}_{-1.8}$$	$$^{+2.8}_{-2.7}$$	$$^{+6.5}_{-5.9}$$	0.8	0.1	0.0	0.75	1.8
$$5$$	$$ 0.60{-}0.66$$	8.76	1.0	0.38	$$^{+6.6}_{-6.4}$$	$$^{+0.1}_{-1.4}$$	$$^{+2.8}_{-2.7}$$	$$^{+6.2}_{-5.8}$$	0.8	0.1	0.0	0.75	1.8
$$6$$	$$ 0.66{-}0.72$$	5.35	1.3	0.39	$$^{+6.5}_{-6.3}$$	$$^{+0.1}_{-1.2}$$	$$^{+2.9}_{-2.7}$$	$$^{+5.9}_{-5.6}$$	0.8	0.1	0.0	0.75	1.8
$$7$$	$$ 0.72{-}0.78$$	3.27	1.6	0.46	$$^{+6.4}_{-6.2}$$	$$^{+0.1}_{-1.1}$$	$$^{+2.9}_{-2.8}$$	$$^{+5.6}_{-5.3}$$	0.8	0.1	0.0	0.75	1.8
$$8$$	$$ 0.78{-}0.86$$	1.95	1.3	0.45	$$^{+6.3}_{-6.1}$$	$$^{+0.2}_{-1.1}$$	$$^{+2.9}_{-2.8}$$	$$^{+5.4}_{-5.1}$$	0.8	0.0	0.0	0.75	1.8
$$9$$	$$ 0.86{-}0.94$$	1.11	0.96	0.47	$$^{+6.1}_{-6.0}$$	$$^{+0.4}_{-1.1}$$	$$^{+2.8}_{-2.8}$$	$$^{+5.1}_{-4.9}$$	0.7	0.0	0.0	0.75	1.8
$$10$$	$$ 0.94{-}1.02$$	$$ 6.73\times 10^{-1}$$	1.1	0.44	$$^{+6.0}_{-5.9}$$	$$^{+0.5}_{-1.2}$$	$$^{+2.8}_{-2.7}$$	$$^{+4.9}_{-4.7}$$	0.7	0.0	0.0	0.75	1.8
$$11$$	$$ 1.02{-}1.12$$	$$ 3.87\times 10^{-1}$$	0.57	0.39	$$^{+5.9}_{-5.8}$$	$$^{+0.6}_{-1.1}$$	$$^{+2.6}_{-2.6}$$	$$^{+4.7}_{-4.4}$$	0.7	0.0	0.0	0.75	1.8
$$12$$	$$ 1.12{-}1.22$$	$$ 2.14\times 10^{-1}$$	0.65	0.34	$$^{+5.9}_{-5.7}$$	$$^{+0.5}_{-1.0}$$	$$^{+2.4}_{-2.4}$$	$$^{+4.5}_{-4.2}$$	0.7	0.0	0.0	0.75	1.8
$$13$$	$$ 1.22{-}1.34$$	$$ 1.20\times 10^{-1}$$	0.75	0.30	$$^{+6.0}_{-5.7}$$	$$^{+0.4}_{-0.7}$$	$$^{+2.2}_{-2.1}$$	$$^{+4.3}_{-4.0}$$	0.7	0.0	0.0	0.75	1.8
$$14$$	$$ 1.34{-}1.46$$	$$ 6.32\times 10^{-2}$$	0.59	0.33	$$^{+6.1}_{-5.9}$$	$$^{+0.3}_{-0.5}$$	$$^{+1.9}_{-1.9}$$	$$^{+4.1}_{-3.9}$$	0.7	0.0	0.0	0.75	1.8
$$15$$	$$ 1.46{-}1.60$$	$$ 3.37\times 10^{-2}$$	0.77	0.34	$$^{+6.3}_{-6.1}$$	$$^{+0.3}_{-0.4}$$	$$^{+1.7}_{-1.6}$$	$$^{+4.0}_{-3.8}$$	0.6	0.0	0.0	0.75	1.8
$$16$$	$$ 1.60{-}1.74$$	$$ 1.78\times 10^{-2}$$	0.98	0.41	$$^{+6.6}_{-6.4}$$	$$^{+0.3}_{-0.4}$$	$$^{+1.4}_{-1.4}$$	$$^{+3.8}_{-3.6}$$	0.6	0.0	0.0	0.75	1.8
$$17$$	$$ 1.74{-}1.90$$	$$ 9.00\times 10^{-3}$$	1.4	0.45	$$^{+7.0}_{-6.9}$$	$$^{+0.4}_{-0.4}$$	$$^{+1.2}_{-1.2}$$	$$^{+3.7}_{-3.5}$$	0.6	0.0	0.0	0.75	1.8
$$18$$	$$ 1.90{-}2.08$$	$$ 4.30\times 10^{-3}$$	1.9	0.43	$$^{+7.6}_{-7.5}$$	$$^{+0.3}_{-0.4}$$	$$^{+1.1}_{-1.1}$$	$$^{+3.6}_{-3.4}$$	0.6	0.0	0.0	0.75	1.8
$$19$$	$$ 2.08{-}2.26$$	$$ 2.13v 10^{-3}$$	2.6	0.42	$$^{+8.4}_{-8.2}$$	$$^{+0.3}_{-0.3}$$	$$^{+0.9}_{-1.0}$$	$$^{+3.5}_{-3.4}$$	0.7	0.0	0.0	0.75	1.8
$$20$$	$$ 2.26{-}2.48$$	$$ 9.74\times 10^{-4}$$	3.5	0.44	$$^{+9.4}_{-9.2}$$	$$^{+0.3}_{-0.2}$$	$$^{+0.9}_{-0.9}$$	$$^{+3.4}_{-3.3}$$	0.7	0.0	0.0	0.75	1.8
$$21$$	$$ 2.48{-}2.72$$	$$ 3.74\times 10^{-4}$$	5.4	0.56	$$^{+10.7}_{-10.4}$$	$$^{+0.2}_{-0.2}$$	$$^{+0.8}_{-0.8}$$	$$^{+3.3}_{-3.2}$$	0.8	0.0	0.0	0.75	1.8
$$22$$	$$ 2.72{-}2.98$$	$$ 1.33\times 10^{-4}$$	8.8	0.72	$$^{+12.1}_{-11.6}$$	$$^{+0.3}_{-0.2}$$	$$^{+0.8}_{-0.8}$$	$$^{+3.2}_{-3.2}$$	0.8	0.0	0.0	0.75	1.8
$$23$$	$$ 2.98{-}3.26$$	$$ 5.84\times 10^{-5}$$	13	0.77	$$^{+13.9}_{-13.2}$$	$$^{+0.5}_{-0.2}$$	$$^{+0.7}_{-0.7}$$	$$^{+3.2}_{-3.1}$$	0.8	0.0	0.1	0.75	1.8
$$24$$	$$ 3.26{-}3.58$$	$$ 1.52\times 10^{-5}$$	23	1.4	$$^{+17.2}_{-16.9}$$	$$^{+0.5}_{-0.2}$$	$$^{+0.7}_{-0.7}$$	$$^{+3.2}_{-3.0}$$	0.9	0.0	0.1	0.75	1.8
$$25$$	$$ 3.58{-}4.20$$	$$ 5.57\times 10^{-6}$$	29	1.2	$$^{+26.2}_{-26.6}$$	$$^{+0.7}_{-0.2}$$	$$^{+0.6}_{-0.6}$$	$$^{+2.6}_{-2.8}$$	2.1	0.0	0.3	0.75	1.8

**Table 4:** Measured double-differential three-jet cross-section, $\sigma$, for $R=0.6{}$ jets and $2\le \left| Y^{*}\right| {}<4$, along with uncertainties in the measurement. All uncertainties are given in %, where $\delta _\text {stat}^\text {data}$ ($\delta _\text {stat}^\text {MC}$) are the statistical uncertainties in the data (MC simulation). The $\gamma$ components are the uncertainty in the jet energy calibration from the in situ, the pileup, the close-by jet, and flavour components. The $u$ components show the uncertainty for the jet energy and angular resolution, the unfolding, the quality selection, and the luminosity. While all columns are uncorrelated with each other, the in situ, pileup, and flavour uncertainties shown here are the sum in quadrature of multiple uncorrelated components

$m_{jjj}$ (bin #)	$m_{jjj}$-range (TeV)	$\sigma$ (pb/GeV)	$\delta _{\text {stat}}^{\text {data}}$ (%)	$\delta _{\text {stat}}^{\text {MC}}$ (%)	$\gamma _{\text {in-situ}}$ (%)	$\gamma _{\text {pileup}}$ (%)	$\gamma _{\text {close-by}}$ (%)	$\gamma _{\text {flavour}}$ (%)	$u_{\text {JER}}$ (%)	$u_{\text {JAR}}$ (%)	$u_{\text {unfold}}$ (%)	$u_{\text {qual.}}$ (%)	$u_{\text {lumi}}$ (%)
$$1$$	$$ 0.42{-}0.46$$	36.3	2.1	0.90	$$^{+7.0}_{-7.2}$$	$$^{+0.7}_{-4.4}$$	$$^{+4.0}_{-4.0}$$	$$^{+6.9}_{-6.8}$$	1.8	0.8	0.0	0.75	1.8
$$2$$	$$ 0.46{-}0.50$$	35.9	2.0	0.76	$$^{+7.0}_{-7.1}$$	$$^{+0.5}_{-3.3}$$	$$^{+4.0}_{-3.9}$$	$$^{+6.8}_{-6.6}$$	1.9	0.8	0.0	0.75	1.8
$$3$$	$$ 0.50{-}0.54$$	29.5	2.1	0.76	$$^{+6.9}_{-7.0}$$	$$^{+0.4}_{-2.5}$$	$$^{+3.9}_{-3.8}$$	$$^{+6.7}_{-6.5}$$	1.9	0.8	0.0	0.75	1.8
$$4$$	$$ 0.54{-}0.60$$	19.7	1.8	0.59	$$^{+6.9}_{-6.9}$$	$$^{+0.4}_{-1.9}$$	$$^{+3.8}_{-3.7}$$	$$^{+6.5}_{-6.3}$$	1.8	0.7	0.0	0.75	1.8
$$5$$	$$ 0.60{-}0.66$$	11.6	1.7	0.51	$$^{+6.8}_{-6.7}$$	$$^{+0.3}_{-1.6}$$	$$^{+3.7}_{-3.6}$$	$$^{+6.2}_{-6.0}$$	1.7	0.6	0.0	0.75	1.8
$$6$$	$$ 0.66{-}0.72$$	6.99	2.0	0.40	$$^{+6.7}_{-6.5}$$	$$^{+0.3}_{-1.4}$$	$$^{+3.6}_{-3.4}$$	$$^{+6.0}_{-5.7}$$	1.6	0.5	0.0	0.75	1.8
$$7$$	$$ 0.72{-}0.78$$	4.20	2.0	0.47	$$^{+6.5}_{-6.3}$$	$$^{+0.4}_{-1.4}$$	$$^{+3.5}_{-3.3}$$	$$^{+5.8}_{-5.4}$$	1.5	0.4	0.0	0.75	1.8
$$8$$	$$ 0.78{-}0.86$$	2.55	1.7	0.42	$$^{+6.4}_{-6.1}$$	$$^{+0.5}_{-1.3}$$	$$^{+3.4}_{-3.1}$$	$$^{+5.5}_{-5.2}$$	1.4	0.3	0.0	0.75	1.8
$$9$$	$$ 0.86{-}0.94$$	1.43	2.0	0.47	$$^{+6.3}_{-6.0}$$	$$^{+0.5}_{-1.3}$$	$$^{+3.2}_{-3.0}$$	$$^{+5.3}_{-4.9}$$	1.3	0.3	0.0	0.75	1.8
$$10$$	$$ 0.94{-}1.02$$	$$ 8.60\times 10^{-1}$$	1.0	0.47	$$^{+6.1}_{-5.9}$$	$$^{+0.6}_{-1.4}$$	$$^{+3.0}_{-2.8}$$	$$^{+5.0}_{-4.7}$$	1.2	0.2	0.0	0.75	1.8
$$11$$	$$ 1.02{-}1.12$$	$$ 4.96\times 10^{-1}$$	1.2	0.41	$$^{+6.0}_{-5.8}$$	$$^{+0.6}_{-1.4}$$	$$^{+2.8}_{-2.6}$$	$$^{+4.7}_{-4.5}$$	1.1	0.2	0.0	0.75	1.8
$$12$$	$$ 1.12{-}1.22$$	$$ 2.66\times 10^{-1}$$	1.5	0.37	$$^{+5.9}_{-5.7}$$	$$^{+0.7}_{-1.4}$$	$$^{+2.5}_{-2.4}$$	$$^{+4.4}_{-4.2}$$	1.0	0.1	0.0	0.75	1.8
$$13$$	$$ 1.22{-}1.34$$	$$ 1.47\times 10^{-1}$$	0.79	0.32	$$^{+5.9}_{-5.8}$$	$$^{+0.7}_{-1.2}$$	$$^{+2.2}_{-2.2}$$	$$^{+4.1}_{-4.0}$$	0.9	0.1	0.0	0.75	1.8
$$14$$	$$ 1.34{-}1.46$$	$$ 7.89\times 10^{-2}$$	0.95	0.32	$$^{+6.0}_{-5.8}$$	$$^{+0.6}_{-1.0}$$	$$^{+2.0}_{-1.9}$$	$$^{+3.9}_{-3.8}$$	0.9	0.1	0.0	0.75	1.8
$$15$$	$$ 1.46{-}1.60$$	$$ 4.08\times 10^{-2}$$	1.1	0.34	$$^{+6.2}_{-6.0}$$	$$^{+0.4}_{-0.7}$$	$$^{+1.7}_{-1.7}$$	$$^{+3.7}_{-3.5}$$	0.9	0.0	0.0	0.75	1.8
$$16$$	$$ 1.60{-}1.74$$	$$ 2.15\times 10^{-2}$$	0.90	0.39	$$^{+6.6}_{-6.3}$$	$$^{+0.3}_{-0.5}$$	$$^{+1.5}_{-1.4}$$	$$^{+3.5}_{-3.3}$$	0.9	0.0	0.0	0.75	1.8
$$17$$	$$ 1.74{-}1.90$$	$$ 1.09\times 10^{-2}$$	1.2	0.46	$$^{+7.1}_{-6.8}$$	$$^{+0.2}_{-0.3}$$	$$^{+1.3}_{-1.3}$$	$$^{+3.3}_{-3.2}$$	0.8	0.0	0.0	0.75	1.8
$$18$$	$$ 1.90{-}2.08$$	$$ 5.29\times 10^{-3}$$	1.7	0.49	$$^{+7.7}_{-7.4}$$	$$^{+0.2}_{-0.2}$$	$$^{+1.2}_{-1.1}$$	$$^{+3.2}_{-3.0}$$	0.8	0.0	0.0	0.75	1.8
$$19$$	$$ 2.08{-}2.26$$	$$ 2.53\times 10^{-3}$$	2.4	0.53	$$^{+8.5}_{-8.1}$$	$$^{+0.2}_{-0.1}$$	$$^{+1.1}_{-1.0}$$	$$^{+3.1}_{-2.9}$$	0.9	0.0	0.0	0.75	1.8
$$20$$	$$ 2.26{-}2.48$$	$$ 1.17\times 10^{-3}$$	3.2	0.48	$$^{+9.5}_{-9.2}$$	$$^{+0.1}_{-0.1}$$	$$^{+1.0}_{-0.9}$$	$$^{+3.0}_{-2.9}$$	0.9	0.0	0.0	0.75	1.8
$$21$$	$$ 2.48{-}2.72$$	$$ 4.65\times 10^{-4}$$	5.1	0.50	$$^{+10.7}_{-10.5}$$	$$^{+0.1}_{-0.1}$$	$$^{+0.9}_{-0.9}$$	$$^{+2.9}_{-2.9}$$	0.9	0.0	0.0	0.75	1.8
$$22$$	$$ 2.72{-}2.98$$	$$ 1.58\times 10^{-4}$$	7.9	0.61	$$^{+12.2}_{-11.9}$$	$$^{+0.1}_{-0.1}$$	$$^{+0.9}_{-0.8}$$	$$^{+3.0}_{-2.9}$$	0.9	0.0	0.0	0.75	1.8
$$23$$	$$ 2.98{-}3.26$$	$$ 7.07\times 10^{-5}$$	12	0.75	$$^{+13.8}_{-13.6}$$	$$^{+0.1}_{-0.1}$$	$$^{+0.8}_{-0.8}$$	$$^{+3.0}_{-2.9}$$	0.8	0.0	0.0	0.75	1.8
$$24$$	$$ 3.26{-}3.58$$	$$ 1.42\times 10^{-5}$$	22	1.6	$$^{+17.4}_{-16.8}$$	$$^{+0.1}_{-0.1}$$	$$^{+0.8}_{-0.7}$$	$$^{+3.1}_{-3.0}$$	0.8	0.0	0.0	0.75	1.8
$$25$$	$$ 3.58{-}4.20$$	$$ 6.19\times 10^{-6}$$	28	1.2	$$^{+28.3}_{-25.0}$$	$$^{+0.1}_{-0.1}$$	$$^{+0.9}_{-0.7}$$	$$^{+3.6}_{-3.3}$$	2.8	0.0	0.0	0.75	1.8

**Table 5:** Measured double-differential three-jet cross-section, $\sigma$, for $R=0.4{}$ jets and $4\le \left| Y^{*}\right| {}<6$, along with uncertainties in the measurement. All uncertainties are given in %, where $\delta _\text {stat}^\text {data}$ ($\delta _\text {stat}^\text {MC}$) are the statistical uncertainties in the data (MC simulation). The $\gamma$ components are the uncertainty in the jet energy calibration from the in situ, the pileup, the close-by jet, and flavour components. The $u$ components show the uncertainty for the jet energy and angular resolution, the unfolding, the quality selection, and the luminosity. While all columns are uncorrelated with each other, the in situ, pileup, and flavour uncertainties shown here are the sum in quadrature of multiple uncorrelated components

$m_{jjj}$ (bin #)	$m_{jjj}$-range (TeV)	$\sigma$ (pb/GeV)	$\delta _{\text {stat}}^{\text {data}}$ (%)	$\delta _{\text {stat}}^{\text {MC}}$ (%)	$\gamma _{\text {in-situ}}$ (%)	$\gamma _{\text {pileup}}$ (%)	$\gamma _{\text {close-by}}$ (%)	$\gamma _{\text {flavour}}$ (%)	$u_{\text {JER}}$ (%)	$u_{\text {JAR}}$ (%)	$u_{\text {unfold}}$ (%)	$u_{\text {qual.}}$ (%)	$u_{\text {lumi}}$ (%)
$$1$$	$$ 0.54{-}0.60$$	15.0	1.6	1.0	$$^{+8.2}_{-7.6}$$	$$^{+0.0}_{-4.1}$$	$$^{+2.7}_{-2.5}$$	$$^{+7.2}_{-6.4}$$	0.2	0.1	0.0	0.75	1.8
$$2$$	$$ 0.60{-}0.66$$	15.0	1.6	0.81	$$^{+8.2}_{-7.8}$$	$$^{+0.0}_{-3.2}$$	$$^{+2.7}_{-2.6}$$	$$^{+6.8}_{-6.3}$$	0.4	0.1	0.0	0.75	1.8
$$3$$	$$ 0.66{-}0.72$$	12.7	1.3	0.74	$$^{+8.2}_{-8.0}$$	$$^{+0.1}_{-2.4}$$	$$^{+2.7}_{-2.6}$$	$$^{+6.5}_{-6.2}$$	0.6	0.1	0.0	0.75	1.8
$$4$$	$$ 0.72{-}0.80$$	8.89	0.98	0.62	$$^{+8.3}_{-8.2}$$	$$^{+0.2}_{-1.8}$$	$$^{+2.7}_{-2.7}$$	$$^{+6.3}_{-6.1}$$	0.7	0.1	0.0	0.75	1.8
$$5$$	$$ 0.80{-}0.88$$	5.46	1.2	0.57	$$^{+8.4}_{-8.3}$$	$$^{+0.2}_{-1.4}$$	$$^{+2.7}_{-2.7}$$	$$^{+6.1}_{-5.9}$$	0.8	0.1	0.0	0.75	1.8
$$6$$	$$ 0.88{-}0.96$$	3.28	1.5	0.57	$$^{+8.5}_{-8.3}$$	$$^{+0.2}_{-1.3}$$	$$^{+2.7}_{-2.7}$$	$$^{+5.9}_{-5.7}$$	0.8	0.1	0.0	0.75	1.8
$$7$$	$$ 0.96{-}1.06$$	1.82	1.8	0.52	$$^{+8.5}_{-8.3}$$	$$^{+0.3}_{-1.3}$$	$$^{+2.8}_{-2.7}$$	$$^{+5.7}_{-5.4}$$	0.8	0.1	0.0	0.75	1.8
$$8$$	$$ 1.06{-}1.16$$	$$ 9.98\times 10^{-1}$$	1.4	0.61	$$^{+8.6}_{-8.2}$$	$$^{+0.5}_{-1.3}$$	$$^{+2.8}_{-2.7}$$	$$^{+5.5}_{-5.2}$$	0.8	0.1	0.0	0.75	1.8
$$9$$	$$ 1.16{-}1.26$$	$$ 5.84\times 10^{-1}$$	1.1	0.65	$$^{+8.5}_{-8.2}$$	$$^{+0.6}_{-1.3}$$	$$^{+2.8}_{-2.7}$$	$$^{+5.2}_{-4.9}$$	0.8	0.1	0.0	0.75	1.8
$$10$$	$$ 1.26{-}1.38$$	$$ 3.31\times 10^{-1}$$	1.4	0.66	$$^{+8.5}_{-8.2}$$	$$^{+0.7}_{-1.3}$$	$$^{+2.8}_{-2.6}$$	$$^{+5.0}_{-4.8}$$	0.8	0.1	0.0	0.75	1.8
$$11$$	$$ 1.38{-}1.50$$	$$ 1.81\times 10^{-1}$$	1.3	0.64	$$^{+8.6}_{-8.2}$$	$$^{+0.7}_{-1.3}$$	$$^{+2.7}_{-2.6}$$	$$^{+4.8}_{-4.6}$$	0.8	0.1	0.0	0.75	1.8
$$12$$	$$ 1.50{-}1.62$$	$$ 9.89\times 10^{-2}$$	0.92	0.66	$$^{+8.7}_{-8.2}$$	$$^{+0.7}_{-1.2}$$	$$^{+2.6}_{-2.5}$$	$$^{+4.7}_{-4.5}$$	0.8	0.0	0.0	0.75	1.8
$$13$$	$$ 1.62{-}1.76$$	$$ 5.46\times 10^{-2}$$	1.1	0.60	$$^{+8.9}_{-8.3}$$	$$^{+0.6}_{-1.1}$$	$$^{+2.5}_{-2.3}$$	$$^{+4.6}_{-4.3}$$	0.9	0.0	0.0	0.75	1.8
$$14$$	$$ 1.76{-}1.90$$	$$ 2.99\times 10^{-2}$$	1.4	0.57	$$^{+9.0}_{-8.4}$$	$$^{+0.4}_{-0.9}$$	$$^{+2.3}_{-2.1}$$	$$^{+4.5}_{-4.2}$$	0.9	0.0	0.0	0.75	1.8
$$15$$	$$ 1.90{-}2.06$$	$$ 1.57\times 10^{-2}$$	1.1	0.60	$$^{+9.2}_{-8.6}$$	$$^{+0.2}_{-0.7}$$	$$^{+2.1}_{-1.9}$$	$$^{+4.3}_{-4.1}$$	0.9	0.0	0.0	0.75	1.8
$$16$$	$$ 2.06{-}2.22$$	$$ 7.92\times 10^{-3}$$	1.4	0.67	$$^{+9.4}_{-8.9}$$	$$^{+0.2}_{-0.5}$$	$$^{+1.8}_{-1.7}$$	$$^{+4.2}_{-4.0}$$	0.9	0.0	0.0	0.75	1.8
$$17$$	$$ 2.22{-}2.40$$	$$ 4.12\times 10^{-3}$$	1.8	0.76	$$^{+9.8}_{-9.3}$$	$$^{+0.2}_{-0.3}$$	$$^{+1.6}_{-1.5}$$	$$^{+4.1}_{-3.9}$$	1.0	0.0	0.0	0.75	1.8
$$18$$	$$ 2.40{-}2.58$$	$$ 1.99\times 10^{-3}$$	2.7	0.98	$$^{+10.4}_{-9.9}$$	$$^{+0.3}_{-0.2}$$	$$^{+1.4}_{-1.3}$$	$$^{+3.9}_{-3.8}$$	1.1	0.0	0.0	0.75	1.8
$$19$$	$$ 2.58{-}2.78$$	$$ 9.95\times 10^{-4}$$	3.6	1.0	$$^{+11.1}_{-10.5}$$	$$^{+0.3}_{-0.1}$$	$$^{+1.3}_{-1.2}$$	$$^{+3.9}_{-3.7}$$	1.2	0.0	0.0	0.75	1.8
$$20$$	$$ 2.78{-}2.98$$	$$ 4.54\times 10^{-4}$$	5.2	1.2	$$^{+12.1}_{-11.2}$$	$$^{+0.3}_{-0.1}$$	$$^{+1.2}_{-1.1}$$	$$^{+3.8}_{-3.6}$$	1.3	0.0	0.0	0.75	1.8
$$21$$	$$ 2.98{-}3.20$$	$$ 1.91\times 10^{-4}$$	7.7	1.5	$$^{+13.0}_{-12.0}$$	$$^{+0.3}_{-0.1}$$	$$^{+1.1}_{-1.1}$$	$$^{+3.8}_{-3.6}$$	1.4	0.0	0.0	0.75	1.8
$$22$$	$$ 3.20{-}3.42$$	$$ 7.88\times 10^{-5}$$	12	1.6	$$^{+14.0}_{-12.7}$$	$$^{+0.3}_{-0.0}$$	$$^{+1.0}_{-1.0}$$	$$^{+3.8}_{-3.5}$$	1.5	0.0	0.0	0.75	1.8
$$23$$	$$ 3.42{-}3.66$$	$$ 3.33\times 10^{-5}$$	19	1.7	$$^{+15.0}_{-13.5}$$	$$^{+0.3}_{-0.0}$$	$$^{+1.0}_{-1.0}$$	$$^{+3.9}_{-3.5}$$	1.6	0.0	0.0	0.75	1.8
$$24$$	$$ 3.66{-}4.70$$	$$ 5.24\times 10^{-6}$$	23	1.6	$$^{+21.4}_{-21.1}$$	$$^{+0.3}_{-0.0}$$	$$^{+0.8}_{-0.7}$$	$$^{+3.8}_{-3.7}$$	2.4	0.0	0.0	0.75	1.8

**Table 6:** Measured double-differential three-jet cross-section, $\sigma$, for $R=0.6{}$ jets and $4\le \left| Y^{*}\right| {}<6$, along with uncertainties in the measurement. All uncertainties are given in %, where $\delta _\text {stat}^\text {data}$ ($\delta _\text {stat}^\text {MC}$) are the statistical uncertainties in the data (MC simulation). The $\gamma$ components are the uncertainty in the jet energy calibration from the in situ, the pileup, the close-by jet, and flavour components. The $u$ components show the uncertainty for the jet energy and angular resolution, the unfolding, the quality selection, and the luminosity. While all columns are uncorrelated with each other, the in situ, pileup, and flavour uncertainties shown here are the sum in quadrature of multiple uncorrelated components

$m_{jjj}$ (bin #)	$m_{jjj}$-range (TeV)	$\sigma$ (pb/GeV)	$\delta _{\text {stat}}^{\text {data}}$ (%)	$\delta _{\text {stat}}^{\text {MC}}$ (%)	$\gamma _{\text {in-situ}}$ (%)	$\gamma _{\text {pileup}}$ (%)	$\gamma _{\text {close-by}}$ (%)	$\gamma _{\text {flavour}}$ (%)	$u_{\text {JER}}$ (%)	$u_{\text {JAR}}$ (%)	$u_{\text {unfold}}$ (%)	$u_{\text {qual.}}$ (%)	$u_{\text {lumi}}$ (%)
$$1$$	$$ 0.54{-}0.60$$	21.9	2.2	0.98	$$^{+7.9}_{-8.1}$$	$$^{+0.3}_{-5.2}$$	$$^{+3.6}_{-3.6}$$	$$^{+7.1}_{-6.6}$$	2.2	1.8	0.0	0.75	1.8
$$2$$	$$ 0.60{-}0.66$$	21.7	2.2	0.85	$$^{+8.0}_{-8.2}$$	$$^{+0.3}_{-3.7}$$	$$^{+3.6}_{-3.6}$$	$$^{+6.9}_{-6.5}$$	2.2	1.8	0.0	0.75	1.8
$$3$$	$$ 0.66{-}0.72$$	18.0	1.8	0.81	$$^{+8.2}_{-8.2}$$	$$^{+0.3}_{-2.6}$$	$$^{+3.6}_{-3.6}$$	$$^{+6.7}_{-6.4}$$	2.2	1.7	0.0	0.75	1.8
$$4$$	$$ 0.72{-}0.80$$	12.3	1.4	0.61	$$^{+8.2}_{-8.2}$$	$$^{+0.3}_{-1.8}$$	$$^{+3.6}_{-3.6}$$	$$^{+6.5}_{-6.2}$$	2.1	1.6	0.0	0.75	1.8
$$5$$	$$ 0.80{-}0.88$$	7.56	1.7	0.61	$$^{+8.3}_{-8.1}$$	$$^{+0.3}_{-1.4}$$	$$^{+3.6}_{-3.5}$$	$$^{+6.2}_{-6.0}$$	2.1	1.4	0.0	0.75	1.8
$$6$$	$$ 0.88{-}0.96$$	4.49	2.1	0.65	$$^{+8.2}_{-8.0}$$	$$^{+0.3}_{-1.3}$$	$$^{+3.5}_{-3.4}$$	$$^{+6.0}_{-5.7}$$	2.0	1.2	0.0	0.75	1.8
$$7$$	$$ 0.96{-}1.06$$	2.54	1.5	0.47	$$^{+8.2}_{-8.0}$$	$$^{+0.5}_{-1.3}$$	$$^{+3.3}_{-3.2}$$	$$^{+5.7}_{-5.5}$$	1.9	1.1	0.0	0.75	1.8
$$8$$	$$ 1.06{-}1.16$$	1.35	1.9	0.50	$$^{+8.1}_{-7.9}$$	$$^{+0.7}_{-1.5}$$	$$^{+3.2}_{-3.1}$$	$$^{+5.4}_{-5.2}$$	1.8	0.9	0.0	0.75	1.8
$$9$$	$$ 1.16{-}1.26$$	$$ 7.82\times 10^{-1}$$	2.6	0.60	$$^{+8.0}_{-7.8}$$	$$^{+0.8}_{-1.6}$$	$$^{+3.1}_{-3.0}$$	$$^{+5.2}_{-5.0}$$	1.7	0.7	0.0	0.75	1.8
$$10$$	$$ 1.26{-}1.38$$	$$ 4.48\times 10^{-1}$$	1.3	0.62	$$^{+8.0}_{-7.7}$$	$$^{+0.9}_{-1.7}$$	$$^{+3.0}_{-2.8}$$	$$^{+5.0}_{-4.7}$$	1.7	0.6	0.0	0.75	1.8
$$11$$	$$ 1.38{-}1.50$$	$$ 2.40\times 10^{-1}$$	1.6	0.67	$$^{+8.0}_{-7.6}$$	$$^{+1.0}_{-1.6}$$	$$^{+2.8}_{-2.7}$$	$$^{+4.8}_{-4.5}$$	1.6	0.5	0.0	0.75	1.8
$$12$$	$$ 1.50{-}1.62$$	$$ 1.31\times 10^{-1}$$	2.1	0.69	$$^{+8.1}_{-7.6}$$	$$^{+1.1}_{-1.5}$$	$$^{+2.6}_{-2.5}$$	$$^{+4.6}_{-4.3}$$	1.6	0.4	0.0	0.75	1.8
$$13$$	$$ 1.62{-}1.76$$	$$ 7.10\times 10^{-2}$$	1.8	0.64	$$^{+8.1}_{-7.6}$$	$$^{+1.1}_{-1.4}$$	$$^{+2.5}_{-2.3}$$	$$^{+4.5}_{-4.1}$$	1.6	0.3	0.0	0.75	1.8
$$14$$	$$ 1.76{-}1.90$$	$$ 3.92\times 10^{-2}$$	1.3	0.60	$$^{+8.2}_{-7.7}$$	$$^{+1.1}_{-1.1}$$	$$^{+2.2}_{-2.1}$$	$$^{+4.3}_{-4.0}$$	1.6	0.3	0.0	0.75	1.8
$$15$$	$$ 1.90{-}2.06$$	$$ 2.11\times 10^{-2}$$	1.7	0.52	$$^{+8.4}_{-7.9}$$	$$^{+1.0}_{-0.9}$$	$$^{+2.0}_{-1.9}$$	$$^{+4.1}_{-3.8}$$	1.6	0.3	0.0	0.75	1.8
$$16$$	$$ 2.06{-}2.22$$	$$ 1.05\times 10^{-2}$$	1.9	0.63	$$^{+8.6}_{-8.3}$$	$$^{+0.8}_{-0.7}$$	$$^{+1.8}_{-1.7}$$	$$^{+3.9}_{-3.7}$$	1.6	0.3	0.0	0.75	1.8
$$17$$	$$ 2.22{-}2.40$$	$$ 5.18\times 10^{-3}$$	1.7	0.74	$$^{+9.0}_{-8.8}$$	$$^{+0.6}_{-0.5}$$	$$^{+1.6}_{-1.6}$$	$$^{+3.7}_{-3.7}$$	1.6	0.3	0.0	0.75	1.8
$$18$$	$$ 2.40{-}2.58$$	$$ 2.62\times 10^{-3}$$	2.3	0.98	$$^{+9.5}_{-9.4}$$	$$^{+0.4}_{-0.3}$$	$$^{+1.4}_{-1.4}$$	$$^{+3.6}_{-3.6}$$	1.7	0.2	0.0	0.75	1.8
$$19$$	$$ 2.58{-}2.78$$	$$ 1.28\times 10^{-3}$$	3.2	1.1	$$^{+10.1}_{-10.3}$$	$$^{+0.3}_{-0.2}$$	$$^{+1.3}_{-1.3}$$	$$^{+3.5}_{-3.5}$$	1.9	0.2	0.0	0.75	1.8
$$20$$	$$ 2.78{-}2.98$$	$$ 5.77\times 10^{-4}$$	4.7	1.4	$$^{+10.9}_{-11.3}$$	$$^{+0.2}_{-0.2}$$	$$^{+1.2}_{-1.2}$$	$$^{+3.4}_{-3.5}$$	2.0	0.2	0.0	0.75	1.8
$$21$$	$$ 2.98{-}3.20$$	$$ 2.64\times 10^{-4}$$	6.7	1.5	$$^{+11.8}_{-12.3}$$	$$^{+0.1}_{-0.2}$$	$$^{+1.1}_{-1.1}$$	$$^{+3.4}_{-3.6}$$	2.2	0.2	0.0	0.75	1.8
$$22$$	$$ 3.20{-}3.42$$	$$ 1.16\times 10^{-4}$$	10	1.7	$$^{+12.8}_{-13.3}$$	$$^{+0.1}_{-0.1}$$	$$^{+1.1}_{-1.1}$$	$$^{+3.4}_{-3.6}$$	2.3	0.2	0.0	0.75	1.8
$$23$$	$$ 3.42{-}3.66$$	$$ 3.72\times 10^{-5}$$	17	1.9	$$^{+13.9}_{-14.2}$$	$$^{+0.1}_{-0.1}$$	$$^{+1.0}_{-1.0}$$	$$^{+3.5}_{-3.6}$$	2.5	0.2	0.0	0.75	1.8
$$24$$	$$ 3.66{-}4.70$$	$$ 6.07\times 10^{-6}$$	22	1.3	$$^{+24.1}_{-18.9}$$	$$^{+0.1}_{-0.1}$$	$$^{+0.9}_{-0.7}$$	$$^{+4.9}_{-3.6}$$	2.9	0.1	0.0	0.75	1.8

**Table 7:** Measured double-differential three-jet cross-section, $\sigma$, for $R=0.4{}$ jets and $6\le \left| Y^{*}\right| {}<8$, along with uncertainties in the measurement. All uncertainties are given in %, where $\delta _\text {stat}^\text {data}$ ($\delta _\text {stat}^\text {MC}$) are the statistical uncertainties in the data (MC simulation). The $\gamma$ components are the uncertainty in the jet energy calibration from the in situ, the pileup, the close-by jet, and flavour components. The $u$ components show the uncertainty for the jet energy and angular resolution, the unfolding, the quality selection, and the luminosity. While all columns are uncorrelated with each other, the in situ, pileup, and flavour uncertainties shown here are the sum in quadrature of multiple uncorrelated components

$m_{jjj}$ (bin #)	$m_{jjj}$-range (TeV)	$\sigma$ (pb/GeV)	$\delta _{\text {stat}}^{\text {data}}$ (%)	$\delta _{\text {stat}}^{\text {MC}}$ (%)	$\gamma _{\text {in-situ}}$ (%)	$\gamma _{\text {pileup}}$ (%)	$\gamma _{\text {close-by}}$ (%)	$\gamma _{\text {flavour}}$ (%)	$u_{\text {JER}}$ (%)	$u_{\text {JAR}}$ (%)	$u_{\text {unfold}}$ (%)	$u_{\text {qual.}}$ (%)	$u_{\text {lumi}}$ (%)
$$1$$	$$ 0.76{-}0.84$$	4.95	2.4	1.6	$$^{+9.8}_{-9.9}$$	$$^{+0.3}_{-4.1}$$	$$^{+2.5}_{-2.7}$$	$$^{+6.7}_{-6.3}$$	0.3	0.7	0.0	0.75	1.8
$$2$$	$$ 0.84{-}0.94$$	5.00	2.1	1.2	$$^{+10.2}_{-10.1}$$	$$^{+0.3}_{-3.2}$$	$$^{+2.5}_{-2.7}$$	$$^{+6.5}_{-6.1}$$	0.6	0.6	0.0	0.75	1.8
$$3$$	$$ 0.94{-}1.04$$	3.80	1.4	1.1	$$^{+10.7}_{-10.5}$$	$$^{+0.3}_{-2.4}$$	$$^{+2.6}_{-2.6}$$	$$^{+6.3}_{-6.0}$$	0.8	0.5	0.0	0.75	1.8
$$4$$	$$ 1.04{-}1.14$$	2.67	1.4	1.2	$$^{+11.4}_{-10.9}$$	$$^{+0.2}_{-1.8}$$	$$^{+2.6}_{-2.7}$$	$$^{+6.1}_{-6.0}$$	1.0	0.4	0.0	0.75	1.8
$$5$$	$$ 1.14{-}1.26$$	1.74	1.7	1.1	$$^{+12.2}_{-11.4}$$	$$^{+0.2}_{-1.5}$$	$$^{+2.7}_{-2.7}$$	$$^{+6.0}_{-5.8}$$	1.1	0.3	0.0	0.75	1.8
$$6$$	$$ 1.26{-}1.38$$	$$ 9.30\times 10^{-1}$$	2.2	1.1	$$^{+12.8}_{-11.8}$$	$$^{+0.1}_{-1.5}$$	$$^{+2.7}_{-2.7}$$	$$^{+5.8}_{-5.5}$$	1.2	0.3	0.0	0.75	1.8
$$7$$	$$ 1.38{-}1.52$$	$$ 5.52\times 10^{-1}$$	2.7	1.1	$$^{+13.4}_{-12.2}$$	$$^{+0.4}_{-1.7}$$	$$^{+2.8}_{-2.7}$$	$$^{+5.6}_{-5.2}$$	1.3	0.2	0.0	0.75	1.8
$$8$$	$$ 1.52{-}1.66$$	$$ 2.88\times 10^{-1}$$	4.0	1.2	$$^{+13.8}_{-12.6}$$	$$^{+0.6}_{-1.8}$$	$$^{+2.8}_{-2.7}$$	$$^{+5.4}_{-5.0}$$	1.3	0.2	0.0	0.75	1.8
$$9$$	$$ 1.66{-}1.80$$	$$ 1.49\times 10^{-1}$$	2.1	1.3	$$^{+14.2}_{-13.0}$$	$$^{+0.8}_{-1.9}$$	$$^{+2.8}_{-2.8}$$	$$^{+5.2}_{-4.9}$$	1.3	0.2	0.0	0.75	1.8
$$10$$	$$ 1.80{-}1.94$$	$$ 7.94\times 10^{-2}$$	2.5	1.5	$$^{+14.6}_{-13.3}$$	$$^{+1.0}_{-1.8}$$	$$^{+2.8}_{-2.8}$$	$$^{+5.1}_{-4.7}$$	1.2	0.1	0.0	0.75	1.8
$$11$$	$$ 1.94{-}2.10$$	$$ 4.19\times 10^{-2}$$	3.0	1.5	$$^{+15.0}_{-13.6}$$	$$^{+1.1}_{-1.7}$$	$$^{+2.8}_{-2.8}$$	$$^{+5.0}_{-4.6}$$	1.3	0.1	0.0	0.75	1.8
$$12$$	$$ 2.10{-}2.26$$	$$ 2.20\times 10^{-2}$$	3.8	1.7	$$^{+15.6}_{-14.0}$$	$$^{+1.2}_{-1.5}$$	$$^{+2.8}_{-2.8}$$	$$^{+4.9}_{-4.5}$$	1.3	0.1	0.0	0.75	1.8
$$13$$	$$ 2.26{-}2.42$$	$$ 1.21\times 10^{-2}$$	2.4	1.8	$$^{+16.0}_{-14.3}$$	$$^{+1.2}_{-1.3}$$	$$^{+2.8}_{-2.7}$$	$$^{+4.9}_{-4.5}$$	1.4	0.0	0.0	0.75	1.8
$$14$$	$$ 2.42{-}2.58$$	$$ 5.86\times 10^{-3}$$	2.8	2.4	$$^{+16.5}_{-14.8}$$	$$^{+1.1}_{-1.1}$$	$$^{+2.7}_{-2.6}$$	$$^{+4.9}_{-4.5}$$	1.6	0.0	0.0	0.75	1.8
$$15$$	$$ 2.58{-}2.76$$	$$ 3.24\times 10^{-3}$$	3.8	2.1	$$^{+16.9}_{-15.2}$$	$$^{+1.0}_{-0.9}$$	$$^{+2.6}_{-2.5}$$	$$^{+4.8}_{-4.6}$$	1.7	0.0	0.0	0.75	1.8
$$16$$	$$ 2.76{-}2.94$$	$$ 1.53\times 10^{-3}$$	4.8	1.5	$$^{+17.3}_{-15.7}$$	$$^{+0.8}_{-0.7}$$	$$^{+2.5}_{-2.4}$$	$$^{+4.7}_{-4.7}$$	1.7	0.0	0.0	0.75	1.8
$$17$$	$$ 2.94{-}3.12$$	$$ 7.02\times 10^{-4}$$	4.5	2.2	$$^{+17.8}_{-16.2}$$	$$^{+0.6}_{-0.5}$$	$$^{+2.4}_{-2.3}$$	$$^{+4.7}_{-4.7}$$	1.8	0.0	0.0	0.75	1.8
$$18$$	$$ 3.12{-}3.44$$	$$ 2.67\times 10^{-4}$$	5.6	2.1	$$^{+18.5}_{-16.9}$$	$$^{+0.3}_{-0.4}$$	$$^{+2.3}_{-2.2}$$	$$^{+4.6}_{-4.7}$$	2.0	0.0	0.0	0.75	1.8
$$19$$	$$ 3.44{-}3.90$$	$$ 6.67\times 10^{-5}$$	10	2.8	$$^{+19.8}_{-18.2}$$	$$^{+0.0}_{-0.2}$$	$$^{+2.1}_{-2.0}$$	$$^{+4.4}_{-4.5}$$	2.1	0.0	0.0	0.75	1.8
$$20$$	$$ 3.90{-}4.66$$	$$ 4.17\times 10^{-6}$$	30	5.0	$$^{+24.1}_{-24.4}$$	$$^{+0.0}_{-0.5}$$	$$^{+1.2}_{-0.7}$$	$$^{+3.2}_{-3.0}$$	2.8	0.0	0.0	0.75	1.8

**Table 8:** Measured double-differential three-jet cross-section, $\sigma$, for $R=0.6{}$ jets and $6\le \left| Y^{*}\right| {}<8$, along with uncertainties in the measurement. All uncertainties are given in %, where $\delta _\text {stat}^\text {data}$ ($\delta _\text {stat}^\text {MC}$) are the statistical uncertainties in the data (MC simulation). The $\gamma$ components are the uncertainty in the jet energy calibration from the in situ, the pileup, the close-by jet, and flavour components. The $u$ components show the uncertainty for the jet energy and angular resolution, the unfolding, the quality selection, and the luminosity. While all columns are uncorrelated with each other, the in situ, pileup, and flavour uncertainties shown here are the sum in quadrature of multiple uncorrelated components

$m_{jjj}$ (bin #)	$m_{jjj}$-range (TeV)	$\sigma$ (pb/GeV)	$\delta _{\text {stat}}^{\text {data}}$ (%)	$\delta _{\text {stat}}^{\text {MC}}$ (%)	$\gamma _{\text {in-situ}}$ (%)	$\gamma _{\text {pileup}}$ (%)	$\gamma _{\text {close-by}}$ (%)	$\gamma _{\text {flavour}}$ (%)	$u_{\text {JER}}$ (%)	$u_{\text {JAR}}$ (%)	$u_{\text {unfold}}$ (%)	$u_{\text {qual.}}$ (%)	$u_{\text {lumi}}$ (%)
$$1$$	$$ 0.76{-}0.84$$	6.96	3.3	1.4	$$^{+8.9}_{-8.8}$$	$$^{+0.4}_{-5.0}$$	$$^{+3.7}_{-3.6}$$	$$^{+6.5}_{-6.0}$$	2.7	3.6	0.0	0.75	1.8
$$2$$	$$ 0.84{-}0.94$$	7.23	3.0	1.2	$$^{+9.6}_{-9.3}$$	$$^{+0.4}_{-3.8}$$	$$^{+3.8}_{-3.5}$$	$$^{+6.5}_{-5.9}$$	2.9	3.6	0.0	0.75	1.8
$$3$$	$$ 0.94{-}1.04$$	5.74	1.8	1.0	$$^{+10.3}_{-9.8}$$	$$^{+0.4}_{-2.8}$$	$$^{+3.8}_{-3.5}$$	$$^{+6.5}_{-5.8}$$	2.9	3.5	0.0	0.75	1.8
$$4$$	$$ 1.04{-}1.14$$	4.09	2.1	1.2	$$^{+11.1}_{-10.3}$$	$$^{+0.4}_{-2.2}$$	$$^{+3.8}_{-3.5}$$	$$^{+6.5}_{-5.7}$$	2.9	3.4	0.0	0.75	1.8
$$5$$	$$ 1.14{-}1.26$$	2.50	2.5	1.2	$$^{+11.7}_{-10.8}$$	$$^{+0.4}_{-2.0}$$	$$^{+3.8}_{-3.5}$$	$$^{+6.3}_{-5.7}$$	3.0	3.2	0.0	0.75	1.8
$$6$$	$$ 1.26{-}1.38$$	1.34	3.2	1.2	$$^{+12.3}_{-11.2}$$	$$^{+0.6}_{-1.8}$$	$$^{+3.8}_{-3.5}$$	$$^{+6.1}_{-5.6}$$	3.0	3.0	0.0	0.75	1.8
$$7$$	$$ 1.38{-}1.52$$	$$ 7.28\times 10^{-1}$$	3.2	1.1	$$^{+12.6}_{-11.6}$$	$$^{+1.0}_{-1.7}$$	$$^{+3.7}_{-3.5}$$	$$^{+5.9}_{-5.5}$$	2.9	2.7	0.0	0.75	1.8
$$8$$	$$ 1.52{-}1.66$$	$$ 3.81\times 10^{-1}$$	3.2	1.2	$$^{+12.7}_{-11.9}$$	$$^{+1.2}_{-1.5}$$	$$^{+3.6}_{-3.4}$$	$$^{+5.6}_{-5.4}$$	2.8	2.3	0.0	0.75	1.8
$$9$$	$$ 1.66{-}1.80$$	$$ 2.19\times 10^{-1}$$	4.2	1.0	$$^{+12.8}_{-12.2}$$	$$^{+1.4}_{-1.4}$$	$$^{+3.4}_{-3.4}$$	$$^{+5.3}_{-5.3}$$	2.7	2.0	0.0	0.75	1.8
$$10$$	$$ 1.80{-}1.94$$	$$ 1.10\times 10^{-1}$$	4.6	1.3	$$^{+12.8}_{-12.4}$$	$$^{+1.6}_{-1.4}$$	$$^{+3.2}_{-3.4}$$	$$^{+5.0}_{-5.3}$$	2.7	1.6	0.0	0.75	1.8
$$11$$	$$ 1.94{-}2.10$$	$$ 6.00\times 10^{-2}$$	2.8	1.4	$$^{+13.0}_{-12.5}$$	$$^{+1.7}_{-1.5}$$	$$^{+3.0}_{-3.4}$$	$$^{+4.8}_{-5.2}$$	2.7	1.4	0.0	0.75	1.8
$$12$$	$$ 2.10{-}2.26$$	$$ 3.15\times 10^{-2}$$	3.6	1.7	$$^{+13.2}_{-12.6}$$	$$^{+1.8}_{-1.8}$$	$$^{+2.9}_{-3.3}$$	$$^{+4.7}_{-5.1}$$	2.8	1.1	0.0	0.75	1.8
$$13$$	$$ 2.26{-}2.42$$	$$ 1.74\times 10^{-2}$$	4.9	2.0	$$^{+13.5}_{-12.8}$$	$$^{+1.9}_{-2.0}$$	$$^{+2.8}_{-3.1}$$	$$^{+4.7}_{-5.0}$$	3.0	0.9	0.0	0.75	1.8
$$14$$	$$ 2.42{-}2.58$$	$$ 8.55\times 10^{-3}$$	4.5	2.2	$$^{+13.9}_{-13.0}$$	$$^{+2.1}_{-2.1}$$	$$^{+2.7}_{-3.0}$$	$$^{+4.7}_{-4.9}$$	3.1	0.8	0.0	0.75	1.8
$$15$$	$$ 2.58{-}2.76$$	$$ 4.40\times 10^{-3}$$	3.5	1.9	$$^{+14.3}_{-13.3}$$	$$^{+2.3}_{-2.2}$$	$$^{+2.6}_{-2.8}$$	$$^{+4.7}_{-4.8}$$	3.3	0.8	0.0	0.75	1.8
$$16$$	$$ 2.76{-}2.94$$	$$ 2.24\times 10^{-3}$$	4.8	1.9	$$^{+14.7}_{-13.6}$$	$$^{+2.5}_{-2.1}$$	$$^{+2.5}_{-2.7}$$	$$^{+4.7}_{-4.8}$$	3.4	0.7	0.0	0.75	1.8
$$17$$	$$ 2.94{-}3.12$$	$$ 1.09\times 10^{-3}$$	6.8	1.9	$$^{+15.0}_{-13.9}$$	$$^{+2.6}_{-2.0}$$	$$^{+2.4}_{-2.5}$$	$$^{+4.8}_{-4.7}$$	3.6	0.7	0.0	0.75	1.8
$$18$$	$$ 3.12{-}3.44$$	$$ 4.01\times 10^{-4}$$	8.8	1.9	$$^{+15.6}_{-14.5}$$	$$^{+2.8}_{-1.9}$$	$$^{+2.2}_{-2.3}$$	$$^{+4.7}_{-4.6}$$	3.9	0.7	0.0	0.75	1.8
$$19$$	$$ 3.44{-}3.90$$	$$ 7.76\times 10^{-5}$$	12	2.7	$$^{+16.7}_{-15.7}$$	$$^{+3.0}_{-1.7}$$	$$^{+2.0}_{-2.1}$$	$$^{+4.7}_{-4.3}$$	4.6	0.6	0.0	0.75	1.8
$$20$$	$$ 3.90{-}4.66$$	$$ 1.17\times 10^{-5}$$	19	4.3	$$^{+24.1}_{-21.6}$$	$$^{+3.3}_{-1.4}$$	$$^{+1.3}_{-1.1}$$	$$^{+5.6}_{-3.1}$$	9.7	0.6	0.0	0.75	1.8

**Table 9:** Measured double-differential three-jet cross-section, $\sigma$, for $R=0.4{}$ jets and $8\le \left| Y^{*}\right| {}<10$, along with uncertainties in the measurement. All uncertainties are given in %, where $\delta _\text {stat}^\text {data}$ ($\delta _\text {stat}^\text {MC}$) are the statistical uncertainties in the data (MC simulation). The $\gamma$ components are the uncertainty in the jet energy calibration from the in situ, the pileup, the close-by jet, and flavour components. The $u$ components show the uncertainty for the jet energy and angular resolution, the unfolding, the quality selection, and the luminosity. While all columns are uncorrelated with each other, the in situ, pileup, and flavour uncertainties shown here are the sum in quadrature of multiple uncorrelated components

$m_{jjj}$ (bin #)	$m_{jjj}$-range (TeV)	$\sigma$ (pb/GeV)	$\delta _{\text {stat}}^{\text {data}}$ (%)	$\delta _{\text {stat}}^{\text {MC}}$ (%)	$\gamma _{\text {in-situ}}$ (%)	$\gamma _{\text {pileup}}$ (%)	$\gamma _{\text {close-by}}$ (%)	$\gamma _{\text {flavour}}$ (%)	$u_{\text {JER}}$ (%)	$u_{\text {JAR}}$ (%)	$u_{\text {unfold}}$ (%)	$u_{\text {qual.}}$ (%)	$u_{\text {lumi}}$ (%)
$$1$$	$$ 1.18{-}1.30$$	$$ 8.88\times 10^{-1}$$	3.3	2.8	$$^{+14.4}_{-13.3}$$	$$^{+0.2}_{-2.7}$$	$$^{+2.2}_{-2.1}$$	$$^{+5.8}_{-5.2}$$	1.0	0.8	0.0	0.75	1.8
$$2$$	$$ 1.30{-}1.44$$	$$ 8.13\times 10^{-1}$$	2.6	2.3	$$^{+14.6}_{-14.4}$$	$$^{+0.1}_{-2.3}$$	$$^{+2.3}_{-2.3}$$	$$^{+5.5}_{-5.5}$$	1.0	0.8	0.0	0.75	1.8
$$3$$	$$ 1.44{-}1.58$$	$$ 5.67\times 10^{-1}$$	2.9	2.4	$$^{+15.4}_{-15.6}$$	$$^{+0.1}_{-2.0}$$	$$^{+2.4}_{-2.5}$$	$$^{+5.4}_{-5.7}$$	1.0	0.8	0.0	0.75	1.8
$$4$$	$$ 1.58{-}1.74$$	$$ 3.67\times 10^{-1}$$	3.4	2.6	$$^{+16.7}_{-16.8}$$	$$^{+0.2}_{-1.8}$$	$$^{+2.5}_{-2.6}$$	$$^{+5.3}_{-5.6}$$	1.0	0.7	0.0	0.75	1.8
$$5$$	$$ 1.74{-}1.92$$	$$ 2.04\times 10^{-1}$$	4.2	2.8	$$^{+18.5}_{-17.9}$$	$$^{+0.4}_{-1.7}$$	$$^{+2.6}_{-2.6}$$	$$^{+5.3}_{-5.4}$$	1.0	0.6	0.0	0.75	1.8
$$6$$	$$ 1.92{-}2.12$$	$$ 1.04\times 10^{-1}$$	5.5	2.5	$$^{+20.6}_{-19.1}$$	$$^{+0.5}_{-1.7}$$	$$^{+2.7}_{-2.7}$$	$$^{+5.3}_{-5.1}$$	1.0	0.4	0.0	0.75	1.8
$$7$$	$$ 2.12{-}2.32$$	$$ 4.48\times 10^{-2}$$	8.0	3.8	$$^{+22.6}_{-20.2}$$	$$^{+0.4}_{-1.7}$$	$$^{+2.8}_{-2.8}$$	$$^{+5.4}_{-4.9}$$	1.1	0.3	0.0	0.75	1.8
$$8$$	$$ 2.32{-}2.72$$	$$ 1.67\times 10^{-2}$$	8.7	1.9	$$^{+25.6}_{-21.4}$$	$$^{+0.1}_{-1.7}$$	$$^{+3.0}_{-3.0}$$	$$^{+5.4}_{-4.4}$$	1.6	0.2	0.0	0.75	1.8
$$9$$	$$ 2.72{-}3.14$$	$$ 3.52\times 10^{-3}$$	7.9	3.3	$$^{+30.2}_{-23.6}$$	$$^{+0.2}_{-1.7}$$	$$^{+3.4}_{-3.2}$$	$$^{+5.1}_{-3.6}$$	2.7	0.1	0.0	0.75	1.8
$$10$$	$$ 3.14{-}3.58$$	$$ 6.24\times 10^{-4}$$	18	6.3	$$^{+34.7}_{-27.2}$$	$$^{+0.2}_{-1.8}$$	$$^{+3.6}_{-3.5}$$	$$^{+4.8}_{-3.2}$$	3.7	0.1	0.0	0.75	1.8
$$11$$	$$ 3.58{-}4.18$$	$$ 1.03\times 10^{-4}$$	32	14	$$^{+39.9}_{-32.1}$$	$$^{+0.2}_{-1.8}$$	$$^{+3.9}_{-3.8}$$	$$^{+4.6}_{-3.6}$$	4.4	0.1	0.1	0.75	1.8
$$12$$	$$ 4.18{-}5.50$$	$$ 3.03\times 10^{-6}$$	40	14	$$^{+58.5}_{-42.4}$$	$$^{+0.2}_{-1.9}$$	$$^{+5.4}_{-4.1}$$	$$^{+4.3}_{-8.2}$$	5.0	0.1	0.7	0.75	1.8

**Table 10:** Measured double-differential three-jet cross-section, $\sigma$, for $R=0.6{}$ jets and $8\le \left| Y^{*}\right| {}<10$, along with uncertainties in the measurement. All uncertainties are given in %, where $\delta _\text {stat}^\text {data}$ ($\delta _\text {stat}^\text {MC}$) are the statistical uncertainties in the data (MC simulation). The $\gamma$ components are the uncertainty in the jet energy calibration from the in situ, the pileup, the close-by jet, and flavour components. The $u$ components show the uncertainty for the jet energy and angular resolution, the unfolding, the quality selection, and the luminosity. While all columns are uncorrelated with each other, the in situ, pileup, and flavour uncertainties shown here are the sum in quadrature of multiple uncorrelated components

$m_{jjj}$ (bin #)	$m_{jjj}$-range (TeV)	$\sigma$ (pb/GeV)	$\delta _{\text {stat}}^{\text {data}}$ (%)	$\delta _{\text {stat}}^{\text {MC}}$ (%)	$\gamma _{\text {in-situ}}$ (%)	$\gamma _{\text {pileup}}$ (%)	$\gamma _{\text {close-by}}$ (%)	$\gamma _{\text {flavour}}$ (%)	$u_{\text {JER}}$ (%)	$u_{\text {JAR}}$ (%)	$u_{\text {unfold}}$ (%)	$u_{\text {qual.}}$ (%)	$u_{\text {lumi}}$ (%)
$$1$$	$$ 1.18{-}1.30$$	1.46	3.8	2.3	$$^{+13.6}_{-13.5}$$	$$^{+0.5}_{-5.0}$$	$$^{+4.1}_{-3.7}$$	$$^{+6.0}_{-5.9}$$	3.2	6.2	0.0	0.75	1.8
$$2$$	$$ 1.30{-}1.44$$	1.21	3.4	2.1	$$^{+14.3}_{-13.7}$$	$$^{+0.5}_{-3.8}$$	$$^{+4.2}_{-3.7}$$	$$^{+6.0}_{-5.8}$$	3.6	6.5	0.0	0.75	1.8
$$3$$	$$ 1.44{-}1.58$$	$$ 8.88\times 10^{-1}$$	3.9	2.3	$$^{+15.0}_{-14.0}$$	$$^{+0.6}_{-2.9}$$	$$^{+4.1}_{-3.8}$$	$$^{+5.8}_{-5.6}$$	4.0	6.8	0.0	0.75	1.8
$$4$$	$$ 1.58{-}1.74$$	$$ 5.94\times 10^{-1}$$	4.5	2.4	$$^{+15.9}_{-14.6}$$	$$^{+0.8}_{-2.2}$$	$$^{+4.0}_{-3.8}$$	$$^{+5.6}_{-5.4}$$	4.2	6.8	0.0	0.75	1.8
$$5$$	$$ 1.74{-}1.92$$	$$ 3.44\times 10^{-1}$$	5.5	2.4	$$^{+17.3}_{-15.4}$$	$$^{+1.0}_{-1.8}$$	$$^{+4.0}_{-3.9}$$	$$^{+5.6}_{-5.4}$$	4.3	6.7	0.0	0.75	1.8
$$6$$	$$ 1.92{-}2.12$$	$$ 1.63\times 10^{-1}$$	7.2	3.0	$$^{+19.0}_{-16.3}$$	$$^{+1.0}_{-1.6}$$	$$^{+4.1}_{-3.9}$$	$$^{+5.7}_{-5.4}$$	4.3	6.4	0.0	0.75	1.8
$$7$$	$$ 2.12{-}2.32$$	$$ 6.64\times 10^{-2}$$	6.5	2.9	$$^{+20.7}_{-17.1}$$	$$^{+0.7}_{-1.5}$$	$$^{+4.1}_{-3.9}$$	$$^{+5.9}_{-5.4}$$	4.5	6.2	0.0	0.75	1.8
$$8$$	$$ 2.32{-}2.72$$	$$ 2.59\times 10^{-2}$$	8.1	1.7	$$^{+22.6}_{-18.1}$$	$$^{+0.4}_{-1.4}$$	$$^{+3.8}_{-3.9}$$	$$^{+5.9}_{-5.4}$$	4.9	5.6	0.0	0.75	1.8
$$9$$	$$ 2.72{-}3.14$$	$$ 4.95\times 10^{-3}$$	16	3.3	$$^{+24.8}_{-19.7}$$	$$^{+0.1}_{-1.3}$$	$$^{+3.7}_{-4.1}$$	$$^{+5.8}_{-5.9}$$	5.7	4.3	0.0	0.75	1.8
$$10$$	$$ 3.14{-}3.58$$	$$ 1.12\times 10^{-3}$$	14	5.7	$$^{+27.7}_{-22.0}$$	$$^{+0.0}_{-0.8}$$	$$^{+3.7}_{-4.3}$$	$$^{+5.7}_{-6.1}$$	6.6	3.2	0.0	0.75	1.8
$$11$$	$$ 3.58{-}4.18$$	$$ 1.44\times 10^{-4}$$	33	9.7	$$^{+30.8}_{-25.0}$$	$$^{+0.0}_{-0.2}$$	$$^{+3.4}_{-4.0}$$	$$^{+5.7}_{-5.5}$$	7.3	2.5	0.1	0.75	1.8
$$12$$	$$ 4.18{-}5.50$$	$$ 5.14\times 10^{-6}$$	43	9.8	$$^{+36.0}_{-34.1}$$	$$^{+1.2}_{-0.2}$$	$$^{+1.4}_{-2.8}$$	$$^{+8.6}_{-2.8}$$	6.5	1.9	0.8	0.75	1.8
